# Synthesis of Chiral 1,4,2-Oxazaphosphepines

**DOI:** 10.3390/molecules200813794

**Published:** 2015-07-29

**Authors:** Oscar Salgado-Escobar, Leticia Chavelas-Hernández, Blanca E. Domínguez-Mendoza, Irma Linzaga-Elizalde, Mario Ordoñez

**Affiliations:** Centro de Investigaciones Químicas, Universidad Autónoma del Estado de Morelos, Av. Universidad 1001, 62209 Cuernavaca, Morelos, Mexico; E-Mails: ose@uaem.mx (O.S.-E.); letychh@gmail.com (L.C.-H.); bed@uaem.mx (B.E.D.-M.)

**Keywords:** 1,4,2-oxazaphosphepines, 1,3-benzoxazines, chiral *o*-hydroxybenzylamines, aminophenols

## Abstract

Synthesis and structural characterization of 1,4,2-oxazaphosphepines is described. The 1,4,2-oxazaphosphepines were obtained from reaction of chiral 1,3-benzoxazines with dichlorophenylphosphine or triethyl phosphite. The configuration of some of these compounds was stablished by X-ray analysis.

## 1. Introduction

**Figure 1:**
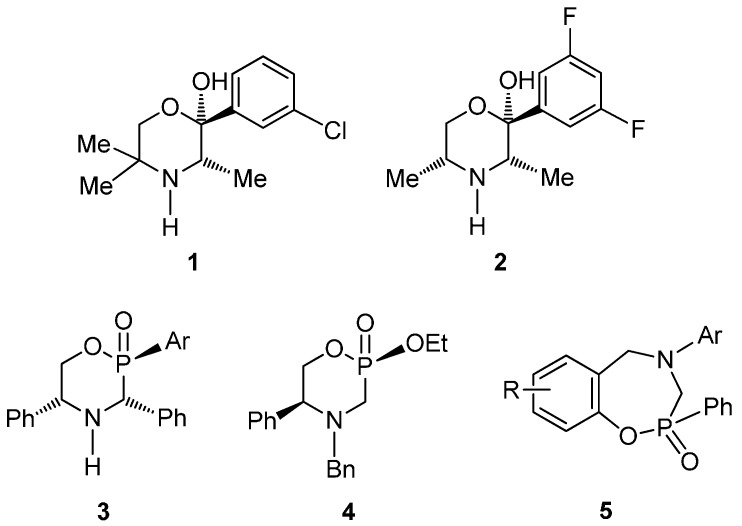
2-Arylmorpholinols, α-aminophosphonic and α-aminophosphinic derivatives.

The 2-arylmorpholinol **1** and **2** possess a strong specific affinity toward the noradrenergic system with application in the treatment of depression and attention deficit hyperactivity disorder (ADHD). On the other hand, the α-aminophosphonic and α-aminophosphinic acids are currently attracting interest in organic and medicinal chemistry, as well as in agriculture, due to their important biological and pharmacological properties, and have been used as key synthetic intermediates for the preparation of more complex compounds [1,2,3,4,5,6]. The great importance of this type of compounds has allowed organic chemists to report numerous procedures regarding their racemic or stereoselective synthesis [7,8,9,10,11,12]. The phosphorus heterocycles type **3** can be considered as analogues of 2-arylmorpholinol **1**, and may be useful as intermediates in the synthesis of α-aminophosphinic acids [13,14]. We described [15] in previous publications the synthesis of enantiopure (2*S*,5*S*)-4-benzyl-2-ethoxy-2-oxo-5-phenyl-1,4,2-oxazaphosphinane **4** from (*S*)-phenylglycinol [16]; however, to the best of our knowledge, the synthesis of 1,4,2-oxazaphosphepine 2-oxides type **5** has been less explored [17] (Figure 1). These benzo derivatives could be considered as a restructured ring system of **3** with possible applications in medicinal chemistry and organic synthesis.

As a part of our ongoing efforts in the discovery and synthesis of new phosphorus heterocycles conformational constraints [18,19], we report herein the preparation and conformational study of several [1,2,4] oxazaphosphepine 2-oxides.

## 2. Results and Discussion

**Scheme 1:**
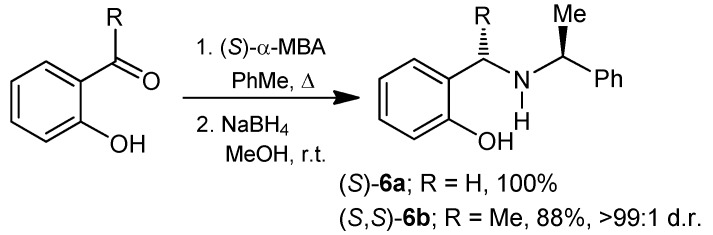
Synthesis of the compounds **6a** and **6b**.

For the synthesis of 1,4,2-oxazaphosphepine 2-oxides, initially we carried out the preparation of chiral *o*-hydroxybenzylamines **6**. Following the procedure described in the literature, the reaction of *o*-salycilaldehyde and *o*-hydroxyacetophenone with (*S*)-α-methylbenzylamine in toluene at reflux gave the corresponding imines, which without additional purification were reacted with NaBH_4_ in methanol at room temperature, obtaining the *o*-hydroxybenzylamines (*S*)-**6a** [20] and (*S*,*S*)-**6b** [21] in excellent yield and >99:1 diastereoisomeric ratio (Scheme 1).

**Scheme 2:**
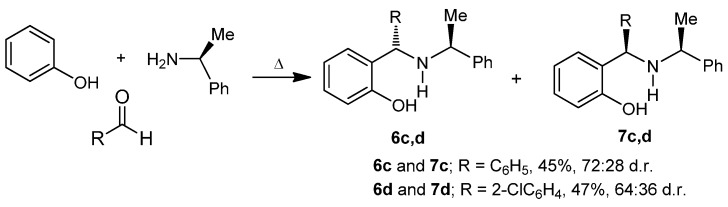
Synthesis of the compounds **6c**, **7c**, **6d** and **7d**.

On the other hand, the “one-pot” three-component reaction of phenol with aryl aldehydes and (*S*)-α-methylbenzylamine under heating and solvent-free conditions afforded the *o*-hydroxybenzylamines **6c**,**d** and **7c**,**d**. In all cases, the diastereoisomeric ratio was similar to those obtained in the nucleophilic addition of other reagents onto imines bearing (*S*)-α-methylbenzylamine [22]. The “one-pot” three-component reaction of phenol with benzaldehyde and (*S*)-α-methylbenzylamine, gave the *o*-hydroxybenzylamines (*S*,*S*)-**6c** and (*R*,*S*)-**7c** in 45% yield and 72:28 diastereoisomeric ratio, with predominance of the (*S*,*S*)-**6c** diastereoisomer. The reaction of phenol with 2-chlorobenzaldehyde and (*S*)-α-methylbenzylamine, produced the *o*-hydroxybenzylamines (*R*,*S*)-**6d** and (*S*,*S*)-**7d** in 47% yield and 64:36 diastereoisomeric ratio, now with a predominance of the (*R*,*S*)-**6d** diastereoisomer, derived of the priority change of the substituents on the new stereogenic center, and not by the nucleophilic attack on the intermediate imine (Scheme 2). The pure diastereoisomers (*S*,*S*)-**6c**, (*R*,*S*)-**7c**, (*R*,*S*)-**6d** and (*S*,*S*)-**7d** were obtained after purification and separation by column chromatography.

**Figure 2:**
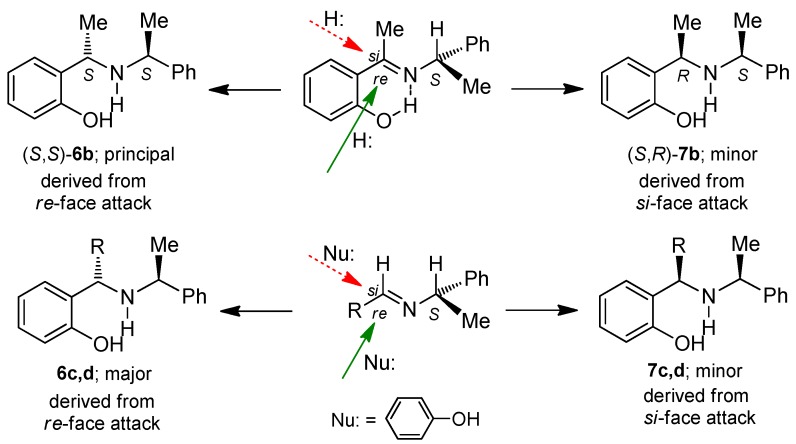
Proposed mechanism for nucleophilic attack of NaBH_4_ and phenol onto imines.

The diastereoselectivity in the synthesis of *o*-hydroxybenzylamine (*S*,*S*)-**6b** considering that in the Schiff bases the C-H bond of the chiral amine in the most stable conformation is eclipsed with the N-C-H fragment, as would be expected from the 1,3-allylic strain model [23], and the conformations with C-Ph and C-Me eclipsed with N-C-H were appreciably higher in energy, and the nucleophilic attack of hydride or the phenol on the imines should take place at the *re* face (less hindered side) to afford the (*S*,*S*)-diastereoisomers as the principal product (Figure 2).

**Scheme 3:**
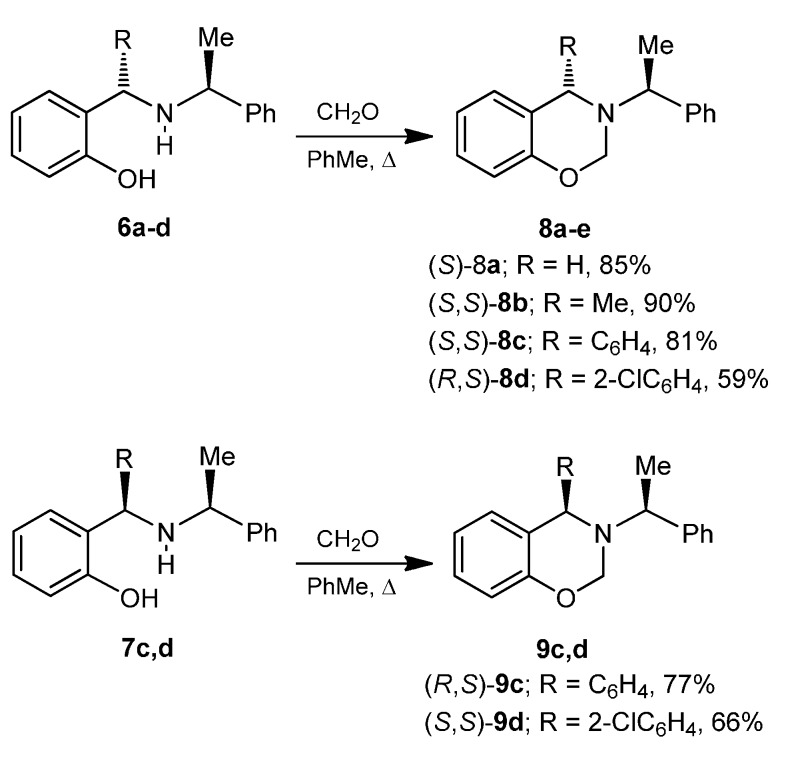
Synthesis of the compounds **8a**–**d** and **9c**,**d**.

Once the *o*-aminophenols **6a**–**d** and **7c**,**d** derivatives were synthesized, the next step was the reaction with formaldehyde in order to obtain the 1,3-benzoxazines **8** and **9**. Following a similar procedure to that described in the literature [24], initially, the reaction of *o*-aminophenol (*S*)-**6a** with formaldehyde in dichloromethane at reflux, afforded the 1,3-benzoxazine (*S*)-**8a** in 85% yield, whereas the reaction of *o*-aminophenol (*S*,*S*)-**6b** under identical conditions, produced the 1,3-benzoxazines (*S*,*S*)-**8b** in 90% yield. In a similar way, the *o*-aminophenols **6c**,**d** and **7c**,**d** were reacted with formaldehyde, obtaining the 1,3-benzoxazines **8c**,**d** and **9c**,**d** in 59% to 81% yield (Scheme 3).

**Figure 3:**
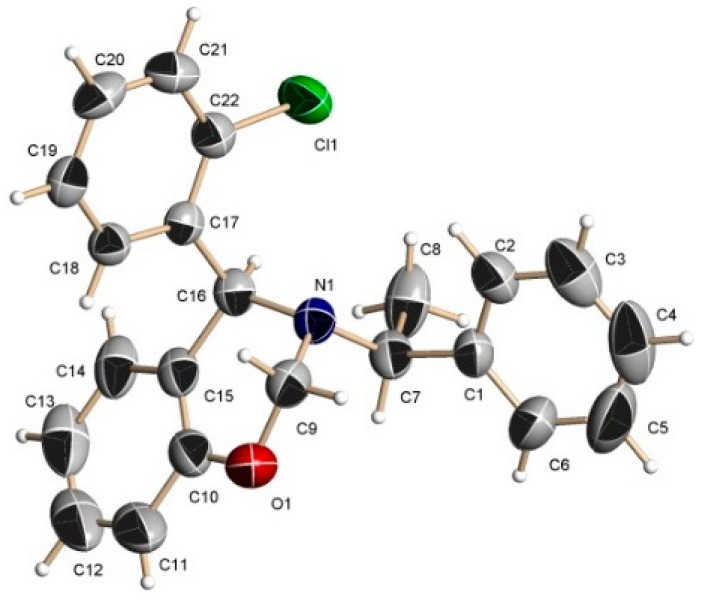
X-ray structure for 1,3-benzoxazine (*S*,*S*)-**9d**.

The absolute configuration of the stereogenic center at C16 of the 1,3-benzoxazines (*S*,*S*)-**8b** and (*S*,*S*)-**8c** was determined by comparison with the enantiomers (*R*,*R*) previously reported in the literature [25], whereas the absolute configuration of the stereogenic center at C16 of the 1,3-benzoxazine 9d was determined as (*S*,*S*) by single crystal X-ray analysis for the minor diastereoisomer [26], which show that the 2-chlorophenyl substituent has an *anti*-disposition to the (α)-methylbenzyl fragment (Figure 3). On these bases, we assumed that the stereochemistry for the major 1,3-benzoxazine is (*R*,*S*).

**Scheme 4:**
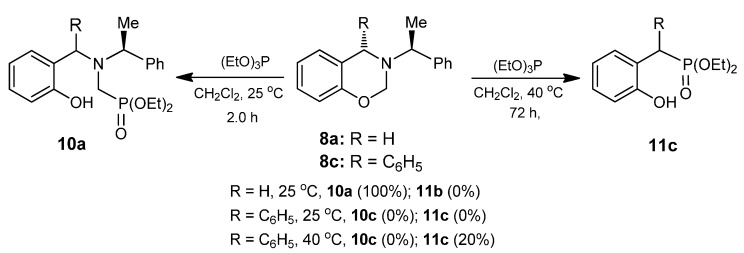
1,3-Benzoxazines reaction with triethyl phosphite.

With the 1,3-benzoxazines **8a**–**d** in hand, the next step was carried out for the reaction with phosphorus nucleophilic reagents. Initially, the 1,3-benzoxazine (*S*)-**8a** was reacted with triethyl phosphite in dichloromethane at room temperature for 2 h, obtaining the α-aminophosphonate (*S*)-**10a** in quantitative yield. On the other hand, the reaction of 1,3-benzoxazine (*S*,*S*)-**8c** with triethyl phosphite in dichloromethane at room temperature did not give the desired product, and when the reaction was carried out at 40 °C for 72 h, the phosphonate *rac*-**11c** was obtained in 20% yield (Scheme 4).

**Scheme 5:**
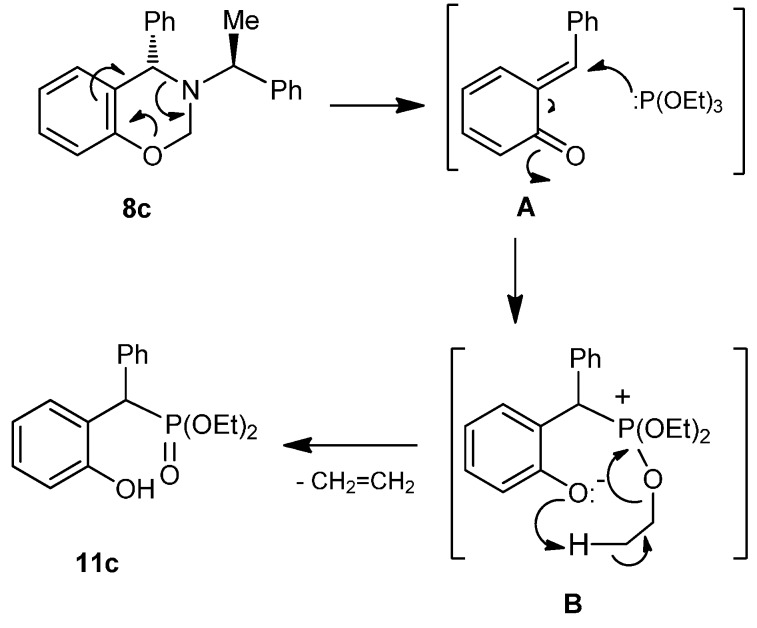
Proposed reaction mechanism for the formation of phosphonate **11c**.

The formation of **11c** can be explained through the formation of enone intermediate **A**, which by nucleophilic reaction with triethyl phosphite gives rise to the formation of intermediate **B**. Finally, the phenolate is protonated generating ethylene and diethylphenyl phosphonate like Michaelis-Arbuzov reaction (Scheme 5) [27,28].

On the other hand, the reaction of the 1,3-benzoxazine (*S*)-**8a** with triethyl phosphite in the presence of BF_3_·OEt_2_ (0.2 eq) in dichloromethane at room temperature, gave the α-aminophosphonate (*S*)-**10a** and the 1,4,2-oxazaphosphepine **12a** in 32 and 15% yield, respectively (Table 1, entry 1). The reaction of 1,3-benzoxazine (*S*,*S*)-**8b** under identical conditions gave the 1,4,2-oxazaphosphepines **13b** and **14b** in 7 and 15% yield, respectively (Table 1, entry 2). In a similar way, the reaction of (*S*,*S*)-**8c** afforded the 1,4,2-oxazaphosphepines **13c** and **14c** in 11 and 16% yield, respectively (Table 1, entry 3). However, the reaction of (*R*,*S*)-**8d** gave the 1,4,2-oxazaphosphepine **13d** in only 6% yield (Table 1, entry 4). When other Lewis acids such as SnCl_4_ and TiCl_4_ were used as catalyst, the reaction did not proceed or very low yields were obtained. Additionally, after several attempts it was not possible to increase the yields.

**Table 1:** Reaction of **8a**–**d** with (EtO)_3_P catalyzed with BF_3_·OEt_2_. 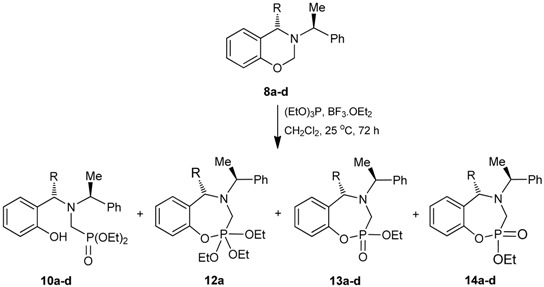

Entry	R	10; Yield (%)	12; Yield (%)	13; Yield (%)	14; Yield (%)
1	**a**: H	32	15	--	--
2	**b**: Me	--	--	7	15
3	**c**: C_6_H_5_	--	--	11	16
4	**d**: 2-ClC_6_H_4_	--	--	6	--

**Figure 4:**
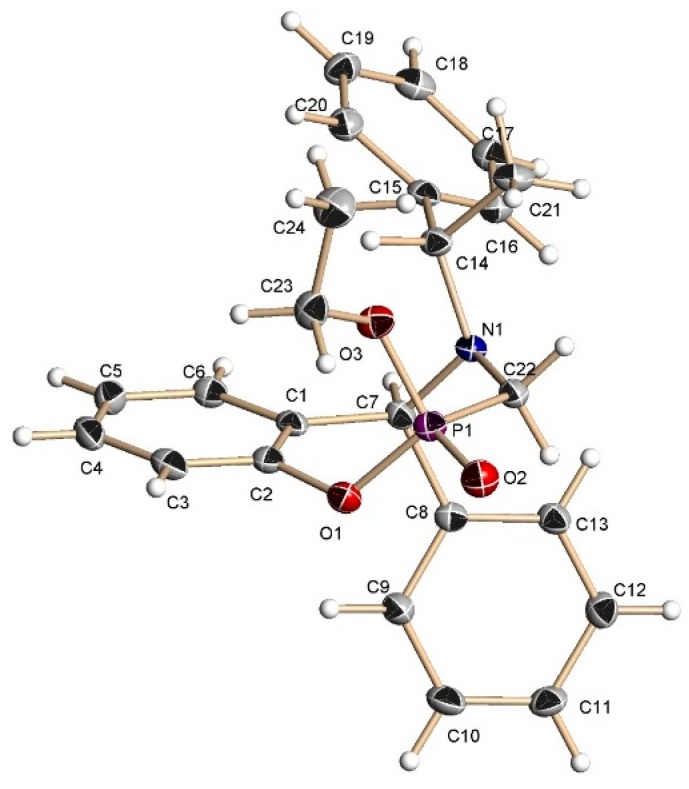
X-ray crystallographic structure of (2*R*,5*S*,1ʹ*S*)-**13c**.

^1^H-, ^13^C-NMR and X-ray analysis [29] for the compound **13c** allowed assigning the configuration as (2*R*,5*S*,1ʹ*S*). Additionally this seven-membered ring has a chair-conformation with the phenyl and the ethoxy groups in *trans*-diaxial disposition (Figure 4).

**Scheme 6:**
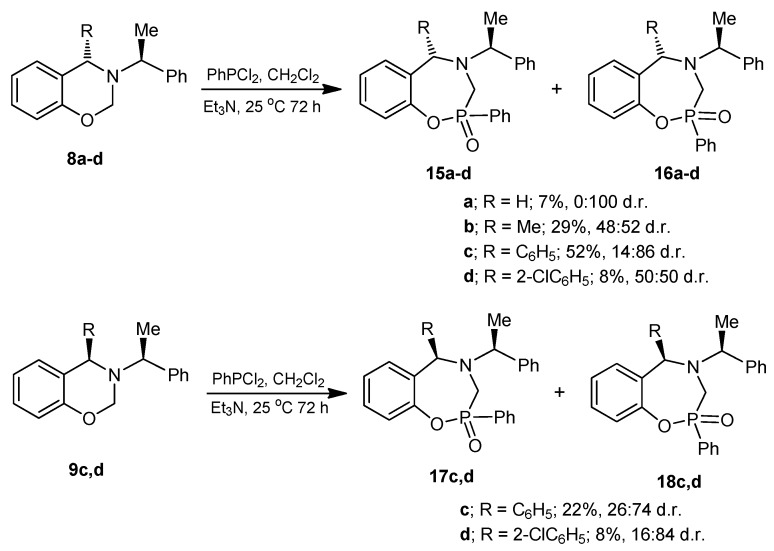
Synthesis of 1,4,2-oxazaphosphepines.

On the other hand, the reaction of chiral 1,3-benzoxazines **8a**–**d** with dichlorophenylphosphine as phosphorus source and triethylamine in dichlorometane at room temperature afforded the diastereoisomeric mixture of 1,4,2-oxazaphosphepines **15a**–**d** and **16a**–**d** in 50:50 to 0:100 diastereoisomeric ratio. In a similar way, the reaction of **9c**,**d** gave the 1,4,2-oxazaphosphepines **17c**,**d** and **18c**,**d** in 26:74 and 16:84 diastereoisomeric ratios, respectively. Most of the compounds were obtained as diastereoisomeric pairs, due to the formation of a new chiral center by the insertion of phosphorus atom (Scheme 6). The compound **15b** could be observed by ^1^H-NMR after purification, but this compound could not be fully characterized. Compounds **17d** and **18d** could not be separated by chromatographic procedure, however, good diastereoselectivity was obtained.

**Figure 5:**
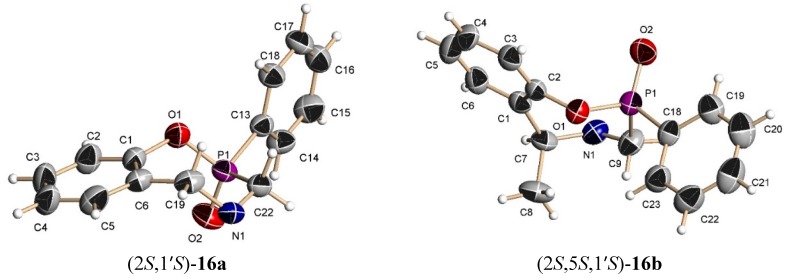

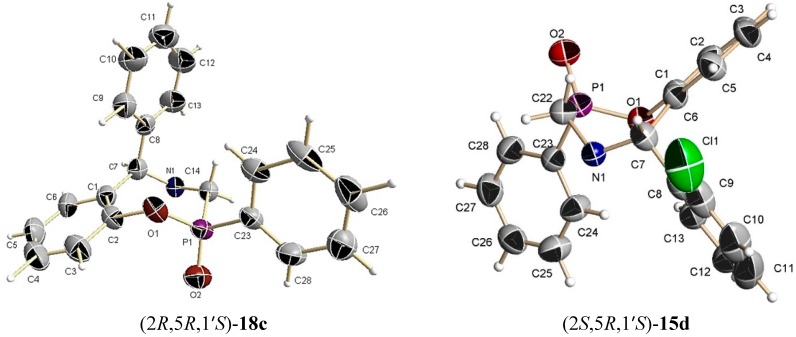
Chair conformations for **16a**, **16b**, and **18c**. Boat conformation for **15d** [34]. The methylbenzyl group was removed to allow for a better appreciation of the conformations.

The X-rays analysis of the compounds **16a**, **16b**, **15d** and **18c** allowed the assignment of the configuration [30,31,32,33], and it was found that the chair conformation is the most stable (Figure 5). The compounds **16a**, **16b** and **18c** exhibit an axial distribution for the P=O moiety. Additionally a *syn*-diaxial distribution was observed between P=O and methylbenzyl fragment. For the compound **15d**, the X-ray structure showed a boat conformation with a 33.7° O-P-C-N angle.

## 3. Experimental Section

### 3.1. General Comments

Reagents were obtained from commercial suppliers and were used without further purification. Melting points were determined in a Fischer Johns apparatus and are uncorrected. NMR spectra were recorded on Varian System instrument (400 MHz for ^1^H and 100 MHz for ^13^C) and Varian Gemini 200 MHz (200 MHz for ^1^H and 50 MHz for ^13^C). The spectra were obtained in CDCl_3_ solution using TMS as internal reference. High resolution CI^+^ and FAB^+^ mass experiments were done in a JEOL HRMStation JHRMS-700. X-ray diffraction studies were performed on a Bruker-APEX diffractometer with a CCD area detector at 100 K (λ_Mo Kα_ = 0.71073 Å, monochromator: graphite). Specific rotations were measured in a Perkin-Elmer 341 polarimeter at room temperature and λ = 589 nm. The purification of compounds was carried out by column chromatography utilizing (silica gel, 230–400 and 70–230) and chromatotron (silica gel Merck 60 PF254 and gypsum) and neutral alumina. The dichloromethane was refluxed on phosphorous pentoxide. Spectroscopic data for **6a** [20] were identical to those reported in the literature. ^1^H- and ^13^C-NMR data for the compound **8a** are identical with those described in the literature for the (*R*) enantiomer [24].

### 3.2. Preparation of Aminophenols

#### 3.2.1. Preparation of 2-{(1*S*)-1-{[(1*S*)-1-Phenyethyl]amino}ethyl}phenol (**6b**)

A mixture of 2-hydroxyacetophenone 1.0 g, 1.37 mL (7.3 mmol), (*S*)-α-methylbenzylamine 0.89 g, 0.93 mL (7.3 mmol) and toluene (25 mL), was heated for 1 h under azeotropic removal of water. The solvent was evaporated under reduced pressure; the crude product was dissolved in methanol (21 mL) and treated with cerium trichloride heptahydrate 1.36 g (3.7 mmol). The solution was cooled at −78 °C, and sodium borohydride 0.55 g (1.5 mmol) was added. The reaction mixture was allowed the room temperature and stirred for 16 h. The solvent was removed under vacuum, the crude product was dissolved in dichloromethane (175 mL), treated with a saturated solution of ammonium chloride (35 mL), and extracted with dichloromethane (3 × 30 mL). The organic layers were dried over anhydrous Na_2_SO_4_, filtered and evaporated under reduced pressure, obtaining (1.72 g, 97%) as a mixture of two diastereoisomers (90:10 d.r.). The mixture was dissolved in ethyl ether (50 mL), washed with 1.5 M hydrochloric acid (5 mL), 1.0 M sodium hydroxide (7 mL), and extracted with ethyl acetate (3 × 30 mL). The organic phase was dried over anhydrous Na_2_SO_4_, filtered and evaporated under reduced pressure, to give the (*S*,*S*)-diastereoisomer (**6b**) [21] (1.55 g, 88%) as a colorless oil. [α]_D_ = −70.9° (*c* = 0.0127, CHCl_3_). ^1^H-NMR (CDCl_3_, 200 MHz): δ 1.34 (d, *J* = 7.0 Hz, 3H), 1.40 (d, *J* = 6.7 Hz, 3H), 3.64 (q, *J* = 6.7 Hz, 1H), 3.70 (q, *J* = 6.7 Hz, 1H), 6.75–7.38 (m, 9H). ^13^C-NMR (CDCl_3_, 50 MHz): δ 23.0, 23.6, 55.5, 56.3, 116.9, 119.3, 126.4, 127.6, 128.4, 128.8, 143.5, 157.6.

#### 3.2.2. Preparation of Aminophenols (**6c**) and (**7c**)

A mixture of benzaldehyde 3.0 g, 2.8 mL (28.3 mmol), phenol 3.2 g (33.9 mmol) and (*S*)-α-methylbenzylamine 3.4 g, 3.6 mL (28.3 mmol), was heated at 60–70 °C for 48 h. The reaction mixture was percolated on a column chromatography, eluting with hexane:EtOAc (98:2), obtaining (**6c**) and (**7c**) (3.85 g, 45%) as a diastereoisomeric mixture 72:28 d.r., which was separated by column chromatography eluting with hexane:EtOAc (99:1), to give both aminophenols (**6c**) as viscous oil (2.8 g, 32%) and (**7c**) as viscous oil (1.1 g, 13%).

*2-[(S)-Phenyl-{[(1S)-1-phenylethyl]amino}methyl]phenol* (**6c**). [α]_D_ = +132.8° (c = 0.0179, CHCl_3_). ^1^H-NMR (CDCl_3_, 200 MHz): δ 1.47 (d, *J* = 6.8 Hz, 3H), 3.85 (q, *J* = 6.8 Hz, 1H), 4.70 (s, 1H), 6.77–7.45 (m, 14H). ^13^C-NMR (CDCl_3_, 50 MHz): δ 23.0, 56.3, 64.9, 117.3, 119.7, 126.8, 127.7, 128.1, 129.1, 129.6, 142.1, 142.4, 157.8.

*2-[(R)-Phenyl-{[(1S)-1-phenylethyl]amino}methyl]phenol* (**7c**). [α]_D_ = −102.1° (*c* = 0.010, CHCl_3_). ^1^H-NMR (CDCl_3_, 200 MHz): δ 1.42 (d, *J* = 6.6 Hz, 3H), 3.71 (q, *J* = 6.6 Hz, 1H), 4.83 (s, 1H), 6.46–7.41 (m, 14 H). ^13^C-NMR (CDCl_3_, 50 MHz): δ 23.2, 55.2, 63.2, 117.0, 119.1, 125.9, 127.3, 128.0, 128.1, 128.3, 129.0, 129.1, 129.2, 140.2, 142.3, 157.8.

#### 3.2.3. Preparation of Aminophenols (**6d**) and (**7d**)

A mixture of phenol 3.0 g (31.9 mmol), *o*-chlorobenzaldehyde 4.48 g (31.9 mmol) and (*S*)-α-methylbenzylamine 3.86 g, 4.05 mL (31.9 mmol), was heated at 60–70 °C for 24 h. After this time, the reaction mixture was purified by column chromatography eluting with hexane:EtOAc (98:2), to give (3.4 g, 47%) as a diastereoisomeric mixture 64:36 d.r., which was separated by column chromatography using a mixture of hexane:EtOAc (99:1) as eluent, obtaining the aminophenols (**6d**) as viscous oil (2.2 g, 30%) and (**7d**) as viscous oil (1.2 g, 17%).

*2-[(R)-2-Chlorophenyl-{[(1S)-1-phenylethyl]amino}methyl]phenol* (**6d**). [α]_D_ = +125.95° (*c* = 0.010, CHCl_3_). ^1^H-NMR (CDCl_3_, 400 MHz): δ 1.49 (d, *J* = 6.8 Hz, 3H), 3.85 (q, *J* = 6.7 Hz, 1H), 5.23 (s, 1H), 6.73–7.41 (m, 14H). ^13^C-NMR (CDCl_3_, 100 MHz): δ 22.2, 56.6, 61.3, 117.4, 119.8, 123.5, 127.2, 127.7, 128.1, 128.8, 129.3, 129.4, 130.0, 130.3, 131.1, 133.4, 137.0, 138.5, 142.5, 158.6. HRMS (CI^+^): *m*/*z* calculated for C_21_H_20_ClNO [M + H] 337.1233; found for [M + H]^+^, *m*/*z* 338.1325.

*2-[(S)-2-Chlorophenyl-{[(1S)-1-phenylethyl]amino}methyl]phenol* (**7d**). [α]_D_ = −63.81° (*c* = 0.0118, CHCl_3_). ^1^H-NMR (CDCl_3_, 200 MHz): δ 1.46 (d, *J* = 6.6 Hz, 3H), 3.75 (q, *J* = 6.6 Hz, 1H), 5.34 (s, 1H), 6.48–7.46 (m, 14H). ^13^C-NMR (CDCl_3_, 50 MHz): δ 22.8, 55.8, 61.0, 117.1, 119.3, 124.1, 127.3, 127.7, 128.0, 128.6, 129.0, 129.2, 129.4, 130.6, 130.8, 134.1, 137.1, 142.4, 158.0. HRMS (CI^+^): *m*/*z* calculated for C_21_H_20_ClNO [M + H] 337.1233; found for [M + H]^+^, *m*/*z* 338.1325.

### 3.3. Preparation of 1,3-Benzoxazines

#### 3.3.1. (4*S*)-4-Methyl-3-[(1ʹ*S*)-1-phenylethyl]-3,4-dihydro-2*H*-1,3-benzoxazine (**8b**)

A mixture of (**6b**) 1.5 g (5.9 mmol), formaldehyde 0.23 g, 0.6 mL (7.7 mmol) and dichloromethane (25 mL), was heated for 1 h under azeotropic removal of water. The solvent was evaporated under reduced pressure and the crude product was purified by column chromatography on neutral alumina, using hexane as eluent, obtaining the compound (**8b**) (1.4 g, 90%) as viscous oil. [α]_D_ = +39.18° (*c* = 0.012, CHCl_3_). ^1^H-NMR (CDCl_3_, 200 MHz): δ 1.36 (d, *J* = 7.0 Hz, 3H), 1.44 (d, *J* = 6.6 Hz, 3H), 3.60 (q, *J* = 6.6 Hz, 1H), 3.88 (q, *J* = 6.6 Hz, 1H), 5.00 (AB system, *J* = 11.0 Hz, 1H), 5.15 (AB system, *J* = 11.0 Hz, 1H), 6.78–7.32 (m, 9H). ^13^C-NMR (CDCl_3_, 50 MHz): δ 22.6, 24.5, 52.4, 59.3, 74.5, 116.7, 120.6, 125.7, 127.3, 127.5, 128.7, 128.8, 145.9, 154.6. HRMS (CI^+^): *m*/*z* calculated for C_17_H_19_NO [M + H] 253.1467; found for [M + H]^+^, *m*/*z* 254.1474.

#### 3.3.2. (4*S*)-4-Phenyl-3-[(1ʹ*S*)-1-phenylethyl]-3,4-dihydro-2*H*-1,3-benzoxazine (**8c**)

A mixture of (**6c**) 0.5 g (1.6 mmol), formaldehyde 60 mg, 0.16 mL (2.1 mmol) and dichloromethane (15 mL), was heated for 1 h under azeotropic removal of water. The solvent was evaporated under reduced pressure and the crude product was purified by recrystallization from cold methanol, to give the compound (**8c**) (420 mg, 81%) as a white solid, mp = 98–100 °C. [α]_D_ = +37.3° (*c* = 0.010, CHCl_3_). ^1^H-NMR (CDCl_3_, 200 MHz): δ 1.51 (d, *J* = 6.6 Hz, 3H), 3.96 (q, *J* = 6.6 Hz, 1H), 4.70 (s, 1H), 4.80 (AB system, *J* = 11.0 Hz, 1H), 5.05 (ABX system, *J* = 10.8, 2.0 Hz, 1H), 6.77–7.45 (m, 14H). ^13^C-NMR (CDCl_3_, 50 MHz): δ 21.6, 59.0, 59.4, 74.5, 116.6, 120.3, 120.4, 127.2, 127.6, 127.9, 128.1, 128.3, 128.5, 128.8, 129.0, 130.4, 143.8, 145.3, 154.6. HRMS (CI^+^): *m*/*z* calculated for C_22_H_21_NO [M + H] 315.1623; found for [M + H]^+^, *m*/*z* 316.1694.

#### 3.3.3. (4*R*)-4-(2-Chlorophenyl)-3-[(1ʹ*S*)-1-phenylethyl]-3,4-dihydro-2*H*-1,3-benzoxazine (**8d**)

A mixture of (**6d**) 1.7 g (4.9 mmol), formaldehyde 180 mg, 0.5 mL, (6.3 mmol) and dichloromethane (25 mL), was heated for 1 h under azeotropic removal of water. The solvent was evaporated under reduced pressure and the crude product was purified by column chromatography using a mixture of hexane:EtOAc (99:1) as eluent, obtaining the compound (**8d**) (1.2 g, 59%) as a colorless highly viscous liquid. [α]_D_ = +66.65° (*c* = 0.0108, CHCl_3_). ^1^H-NMR (CDCl_3_, 200 MHz): δ 1.53 (d, *J* = 6.6 Hz, 3H), 4.34 (q, *J* = 6.7 Hz, 1H), 4.71 (AB system, *J* = 11.0 Hz, 1H), 4.78 (AB system, *J* = 11.0 Hz, 1H), 5.30 (s, 1H), 6.77–7.40 (m, 14 H). ^13^C-NMR (CDCl_3_, 50 MHz): δ 18.4, 58.2, 60.3, 74.4, 116.8, 120.6, 122.0, 126.3, 127.5, 127.8, 128.2, 128.4, 128.6, 128.8, 128.9, 129.2, 130.0, 132.0, 134.6, 141.3, 142.6, 155.4. HRMS (CI^+^): *m*/*z* calculated for C_22_H_20_ClNO [M + H] 349.1233; found for [M + H]^+^, *m*/*z* 350.1321.

#### 3.3.4. (4*R*)-4-Phenyl-3-[(1ʹ*S*)-1-phenylethyl]-3,4-dihydro-2*H*-1,3-benzoxazine (**9c**)

A mixture of (**7c**) 0.5 g (1.6 mmol), formaldehyde 60 mg, 0.16 mL, (2.1 mmol) in dichloromethane (15 mL), was heated for 1 h under azeotropic removal of water. The solvent was evaporated under reduced pressure and the crude product was purified by recrystallization from cold methanol obtaining (**9c**) (0.42 g, 77%) as a white solid, mp = 79–81 °C. [α]_D_ = −68.3° (*c* = 0.010, CHCl_3_). ^1^H-NMR (CDCl_3_, 400 MHz): δ 1.57 (d, *J* = 6.0 Hz, 3H), 4.11 (q, *J* = 6.4 Hz, 1H), 4.37 (ABX system, *J* = 10.4, 2.4 Hz, 1H), 4.57 (AB system, *J* = 10.8 Hz, 1H), 5.24 (s, 1H), 6.88–7.43 (m, 14H). ^13^C-NMR (CDCl_3_, 100 MHz): δ 21.5, 57.1, 57.9, 76.1, 116.7, 119.9, 120.4, 127.0, 127.3, 127.7, 128.0, 128.3, 128.4, 128.9, 129.7, 143.7, 144.0, 154.8.

#### 3.3.5. (4*S*)-4-(2-Chlorophenyl)-3-[(1ʹ*S*)-1-phenylethyl]-3,4-dihydro-2*H*-1,3-benzoxazine (**9d**)

A mixture of (**7d**) 0.63 g (1.8 mmol), formaldehyde 70 mg, 0.19 mL, (2.3 mmol) and dichloromethane (25 mL) was heated for 1 h under azeotropic removal of water. The solvent was evaporated under reduced pressure and crude was purified by column chromatography using hexane:EtOAc (99:1) as eluent, obtaining the compound (**9d**) (340 mg, 66%) as a white solid, mp = 100–104 °C. [α]_D_ = −118.26° (*c* = 0.0108, CHCl_3_). ^1^H-NMR (CDCl_3_, 200 MHz): δ 1.63 (d, *J* = 6.6 Hz, 3H), 4.10 (q, *J* = 6.7 Hz, 1H), 4.34 (ABX system, *J* = 10.6, 1.4 Hz, 1H), 4.64 (AB system, *J* = 10.8 Hz) 5.66 (s, 1H), 6.80–7.47 (m, 14H). ^13^C-NMR (CDCl_3_, 50 MHz): δ 21.3, 56.1, 58.7, 75.6, 116.6, 120.0, 120.5, 126.3, 127.4, 127.7, 128.5, 128.8, 129.2, 130.3, 132.5, 134.6, 140.9, 144.5, 154.9. HRMS (CI^+^): *m*/*z* calculated for C_22_H_20_ClNO [M + H] 349.1233; found for [M + H]^+^, *m*/*z* 350.1349.

### 3.4. Reaction of 1,3-Benzoxazines with Triethyl Phosphite

#### 3.4.1. Synthesis of (*S*)-Diethyl-{[(2-hydroxybenzyl)(1-phenylethyl)amino]methyl}phosphonate (*S*)-**10a**

A mixture of benzoxazine (**8a**) 0.5 g (2.1 mmol), triethyl phosphite 0.34 g, 0.35 mL, (2.1 mmol) and dry dichloromethane (10 mL), was reacted under nitrogen atmosphere at room temperature for 2 h. The solvent was evaporated under reduced pressure. The compound was characterized without purification. The compound (*S*)-**10a**) (0.78 g, 100%) as a colorless oil. [α]_D_ = −33.10° (*c* = 0.010, CHCl_3_). ^1^H-NMR (CDCl_3_, 400 MHz): δ 1.27 (t, *J* = 6.8 Hz, 3H), 1.42 (d, *J* = 7.2 Hz, 1H), 2.67 (ABX system, *J* = 15.6, 12.4 Hz, 1H), 2.94 (ABX system, *J* = 15.6, 11.6 Hz, 1H), 3.89 (AB system, *J* = 14.0 Hz, 1H), 3.95 (AB system, *J* = 14.0 Hz, 1H), 4.00–4.07 (m, 4H), 4.19 (q, *J* = 7.2 Hz, 1H), 6.78–7.37 (m, 9H). ^13^C-NMR (CDCl_3_, 100 MHz): δ 13.6, 16.5 (d, *J*_C/P_ = 5.9 Hz), 44.2 (d, *J*_C/P_ = 163.9 Hz), 55.0 (d, *J*_C/P_ = 4.4 Hz), 57.4 (d, *J*_C/P_ = 11.7 Hz), 62.3 (d, *J*_C/P_ = 5.9 Hz), 62.4 (d, *J* = 5.9 Hz), 116.5, 119.4, 122.0, 127.8, 128.4, 128.5, 128.6, 129.2, 129.7, 139.7, 157.7. ^31^P-NMR (CDCl_3_, 80.95 MHz): δ 26.78. HRMS (CI^+^): *m*/*z* calculated for C_20_H_28_NO_4_P [M + H] 377.1756; found for [M + H]^+^, *m*/*z* 378.1819.

#### 3.4.2. Synthesis of Diethyl-[(2-hydroxyphenyl)(phenyl)methyl]phosphonate (**11c**)

A mixture of benzoxazine (**8c**) 200 mg (0.6 mmol) and triethyl phosphite 100 mg, 0.10 mL, (0.6 mmol) in dry dichloromethane (5 mL) was reacted under nitrogen atmosphere at reflux for 72 h. The solvent was evaporated under reduced pressure. The mixture was purified by column chromatography using hexane:EtOAc (80:20). The compound (**11c**) (40 mg, 20%) was obtained as a white solid, mp = 156–159 °C. [α]_D_ = 0° (*c* = 0.010, CHCl_3_). ^1^H-NMR (CDCl_3_, 400 MHz): δ 1.12 (t, *J* = 7.0 Hz, 3H), 1.15 (t, *J* = 6.8 Hz, 3H), 3.86–4.08 (m, 4H), 4.72 (AB system, *J*_H/P_ = 26.6 Hz, 1H), 7.00–7.52 (m, 9H), 8.89 (br, 1H). ^13^C-NMR (CDCl_3_, 100 MHz): δ 16.3, 47.0 (d, *J*_C/P_ = 136.15 Hz), 63.5 (d, *J*_C/P_ = 7.0 Hz), 64.0 (d, *J*_C/P_ = 7.4 Hz), 118.1, 119.1, 121.0, 127.3, 127.5, 128.6, 128.8, 129.1, 129.8 (d, *J*_C/P_ = 8.1 Hz), 131.0 (d, *J*_C/P_ = 7.7 Hz), 136.5 (d, *J*_C/P_ = 4.35 Hz), 155.0 (d, *J*_C/P_ = 5.85 Hz). ^31^P-NMR (CDCl_3_, 80.95 MHz): δ 28.43. HRMS (CI^+^): *m*/*z* calculated for C_17_H_21_O_4_P [M + H] 320.1177; found for [M + H]^+^, *m*/*z* 321.1033.

### 3.5. General Procedure for the Preparation of 1,4,2-Oxazaphosphepines (**12**), (**13**) and (**14**)

Under anhydrous conditions, the corresponding benzoxazine in dry dichloromethane was treated with boron trifluoride etherate and triethyl phosphite. The reaction mixture was stirred at room temperature for 72 h. The solvent was evaporated under reduced pressure, and the crude was dissolved in ethyl acetate, and treated with a saturated solution of ammonium chloride and stirred for 15 min. The organic phase was extracted with ethyl acetate, and the organic extracts were dried over anhydrous Na_2_SO_4_, and evaporated under reduced pressure. The crude product was purified by column chromatography.

#### 3.5.1. (*S*)-2,2,2-Triethoxy-4-(1-phenylethyl)-2,3,4,5-tetrahydro-1,4,2λ^5^-benzoxazaphosphepine (**12a**)

A mixture of benzoxazine (**8a**) 0.75 g (3.1 mmol) boron trifluoride etherate 80 mg, 0.08 mL (0.6 mmol) and triethyl phosphite 0.52 g, 0.53 mL, (3.1 mmol) in dry dichloromethane (10 mL), was reacted at room temperature for 72 h. The solvent was eliminated and the crude product was purified by column chromatography using hexane: *i*-PrOH (98:2) as eluent, obtaining the compound (**12a**) (155 mg, 15%), as colorless. The compound (**10a**) was also obtained (377 mg, 32%). [α]_D_ = −28.4° (*c* = 0.011, CHCl_3_). ^1^H-NMR (CDCl_3_, 400 MHz): δ 1.24 (t, *J* = 6.8 Hz, 3H), 1.25 (t, *J* = 6.8 Hz, 3H), 1.39 (t, *J* = 6.8 Hz, 3H), 1.41 (d, *J* = 7.2 Hz, 3H), 2.80 (ABX system, *J*_H/P_ = 15.4, 12.6 Hz, 1H), 2.97 (ABX system, *J*_H/P_ = 15.6, 8.4 Hz, 1H), 3.71 (AB system, *J* = 14.8 Hz, 1H), 3.91 (AB system, *J* = 14.8 Hz, 1H), 3.96 (q, *J* = 7.2 Hz, 2H), 4.00 (q, *J* =6.8 Hz, 2H), 4.01 (q, *J* = 7.0 Hz, 2H), 4.19 (q, *J* = 6.8 Hz, 1H) 6.80–7.56 (m, 9H). ^13^C-NMR (CDCl_3_, 100 MHz): δ 14.1, 15.1, 16.60, 16.66, 45.5 (d, *J*_C/P_ = 162.5 Hz), 48.6 (d, *J*_C/P_ = 7.3 Hz), 58.3 (d, *J*_C/P_ = 10.2 Hz), 61.7 (d, *J*_C/P_ = 7.3 Hz), 61.8 (d, *J* = 7.3 Hz), 63.7, 111.3, 120.5, 126.9, 127.8, 128.1, 128.4, 130.7, 142.6, 157.2. ^31^P-NMR (CDCl_3_, 80.95 MHz): δ 10.24. HRMS (CI^+^): *m*/*z* calculated for C_22_H_32_NO_4_P [M + H] 405.2069; found for [M + H]^+^, *m*/*z* 406.2128.

#### 3.5.2. Synthesis of 1,4,2-Oxazaphosphepine 2-oxide (**13b**) and (**14b**)

A mixture of benzoxazine (**8b**) 1.0 g (3.9 mmol), boron trifluoride etherate 110 mg, 0.09 mL, (0.8 mmol) and triethyl phosphite 0.65 g, 0.67 mL, (3.9 mmol) in dry dichloromethane (20 mL), was reacted at room temperature for 72 h. The solvent was eliminated and the crude product was purified by column chromatography using hexane:EtOAc (80:20) as eluent, obtaining the compounds (**13b**) (96 mg, 7% and (**14b**) (204 mg, 15%), both as colorless oil.

*(2R,S)-2-Ethoxy-(5S)-5-methyl-4-[(1ʹS)-1-phenylethyl]-2,3,4,5-tetrahydro-1,4,2-benzoxazaphosphepine 2-oxide* (**13b**). [α]_D_ = −2.2° (*c* = 0.013, CHCl_3_). ^1^H-NMR (CDCl_3_, 400 MHz): δ 1.41 (d, *J* = 6.8 Hz, 3H), 1.43 (d, *J* = 6.8 Hz, 3H), 1.43 (t, *J* = 6.8 Hz, 3H), 3.67 (ABX system, *J*_H/P_ = 16.4, 5.8 Hz, 1H), 3.74 (ABX system, *J*_H/P_ = 16.4, 6.0 Hz, 1H), 3.79 (dq, *J* = 7.2, 5.3 Hz, 2H), 4.31 (q, *J* = 7.2 Hz, 1H), 4.33 (q, *J* = 7.2 Hz, 1H), 6.56–7.35 (m, 9H). ^13^C-NMR (CDCl_3_, 100 MHz): δ 16.4, 16.5, 18.7, 22.7, 41.3 (d, *J*_C/P_ = 125.9 Hz), 59.0, 60.1, 62.1 (d, *J*_C/P_ = 8.8 Hz), 122.5, 122.6, 124.9, 126.9, 127.1, 128.4, 129.3, 131.3, 134.1, 145.6, 147.9. ^31^P-NMR (CDCl_3_, 80.95 MHz): δ 15.52. HRMS (CI^+^): *m*/*z* calculated for C_19_H_24_NO_3_P [M + H] 345.1494; found for [M + H]^+^, *m*/*z* 346.1557.

*(2R,S)-2-Ethoxy-(5S)-5-methyl-4-[(1ʹS)-1-phenylethyl]-2,3,4,5-tetrahydro-1,4,2-benzoxazaphosphepine 2-oxide,* (**14b**). [α]_D_ = +5.40° (*c* = 0.010, CHCl_3_). ^1^H-NMR (CDCl_3_, 400 MHz): δ 1.23 (d, *J* = 7.0 Hz, 3H), 1.32 (d, *J* = 6.8 Hz, 3H), 1.44 (t, *J* = 7.2 Hz, 3H), 3.60 (dq, *J* = 6.8, 6.8 Hz, 1H), 3.75 (q, 7.2 Hz, 1H), 3.82 (ABX system, *J*_H/P_ = 16.8, 1.6 Hz, 1H), 3.85 (ABX system, *J*_H/P_ = 16.8, 3.6 Hz, 1H), 4.05–4.15 (m, 2H), 4.29–4.39 (m, 2H), 6.57–7.35 (m, 9H). ^13^C-NMR (CDCl_3_, 100 MHz): δ 16.3, 16.4, 18.4, 22.7, 41.3 (d, *J*_C/P_ = 123.0 Hz), 58.6, 60.0, 61.5 (d, *J*_C/P_ = 8.8 Hz), 121.9, 122.0, 124.7, 127.0, 127.1, 128.5, 129.0, 131.5, 134.1, 145.5, 148.4. ^31^P-NMR (CDCl_3_, 80.95 MHz): δ 18.75. HRMS (CI^+^): *m*/*z* calculated for C_19_H_24_NO_3_P [M + H] 345.1494; found for [M + H]^+^, *m*/*z* 346.1553.

#### 3.5.3. Synthesis of 1,4,2-Oxazaphosphepine 2-oxide (**13c**) and (**14c**)

A mixture of benzoxazine (**8c**) 0.68 g (2.2 mmol), boron trifluoride etherate 60 mg, 0.05 mL, (0.4 mmol) and triethyl phosphite 0.36 g, 0.37 mL, (2.2 mmol) in dry dichloromethane (5 mL) was reacted at room temperature for 72 h. The solvent was eliminated and the crude product was purified by column chromatography using hexane:EtOAc (80:20) as eluent, obtaining the less polar compound (**13c**) (100 mg, 11%) as yellow oil, and the more polar compound (**14c**) (138 mg, 16%) as a white solid mp = 164–170 ºC. The compound (**14c**) was recrystallized from dichloromethane–hexane to give a crystal for X-ray studies.

*(2S)-2-Ethoxy-(5S)-5-phenyl-4-[(1ʹS)-1-phenylethyl]-2,3,4,5-tetrahydro-1,4,2-benzoxazaphosphepine 2-oxide* (**13c**). [α]_D_ = +74.90° (*c* = 0.010, CHCl_3_). ^1^H-NMR (CDCl_3_, 200 MHz): δ 1.28 (t, *J* = 7.0 Hz, 3H), 1.51 (d, *J* = 6.6 Hz, 3H), 3.32 (ABX system, *J*_H/P_ = 16.2, 8.4 Hz, 1H), 3.57 (ABX system, *J*_H/P_ = 16.2, 3.8 Hz, 1H), 3.99–4.23 (m, 3H), 4.96 (s, 1H), 6.67–7.71 (m, 14H). ^13^C-NMR (CDCl_3_, 50 MHz): δ 16.5 (d, *J*_C/P_ = 5.85 Hz), 21.9, 40.2 (d, *J*_C/P_ = 128.15 Hz), 59.9, 62.4 (d, *J*_C/P_ = 7.3 Hz), 67.8, 123.1, 123.2, 125.5, 127.4, 127.5, 128.1, 128.5, 128.8, 130.3, 130.8, 133.2, 139.7, 145.5. ^31^P-NMR (CDCl_3_, 80.95 MHz): δ 10.97. HRMS (CI^+^): *m*/*z* calculated for C_24_H_26_NO_3_P [M + H] 407.1650; found for [M + H]^+^, *m*/*z* 408.1710.

*(2R)-2-Ethoxy-(5S)-5-phenyl-4-[(1ʹS)-1-phenylethyl]-2,3,4,5-tetrahydro-1,4,2-benzoxazaphosphepine 2-oxide* (**14c**). [α]_D_ = +79.62° (*c* = 0.010, CHCl_3_). ^1^H-NMR (CDCl_3_, 200 MHz): δ 1.25 (t, *J* = 7.0 Hz, 3H), 1.42 (d, *J* = 6.8 Hz, 3H), 3.54 (ABX system, *J*_H/P_ = 16.8, 6.8 Hz, 1H), 3.70 (ABX system, *J*_H/P_ = 16.0, 5.2 Hz, 1H), 3.80 (q, *J* = 6.8 Hz, 1H), 4.03–4.45 (m, 2H), 4.92 (s, 1H), 6.66–7.39 (m, 14H). ^13^C-NMR (CDCl_3_, 50 MHz): δ 16.5 (d, *J*_C/P_ = 5.95 Hz), 22.6, 41.7 (d, *J*_C/P_ = 123.3 Hz), 59.9, 61.6 (d, *J*_C/P_ = 7.95 Hz), 67.2, 122.3, 122.4, 125.3, 127.3, 127.5, 127.6, 128.0, 128.7, 128.9, 130.0, 130.6, 130.7, 138.7, 145.4, 149.1 (d, *J*_C/P_ = 7.2 Hz). ^31^P-NMR (CDCl_3_, 80.95 MHz): δ 13.52. HRMS (CI^+^): *m*/*z* calculated for C_24_H_26_NO_3_P [M + H] 407.1650; found for [M + H]^+^, *m*/*z* 408.1710.

#### 3.5.4. Synthesis of (2*R*,*S*)-2-Ethoxy-(5*R*)-5-(2-chlrophenyl)-4-[(1ʹ*S*)-1-phenylethyl]-2,3,4,5-tetrahydro-1,4,2-benzoxazaphosphepine 2-oxide (**13d**)

A mixture of benzoxazine (**8d**) 0.56 g (1.6 mmol), boron trifluoride etherate 40 mg, 0.04 mL (0.3 mmol) and triethyl phosphite 0.26 g, 0.27 mL, (1.6 mmol) in dry dichloromethane (10 mL) was reacted at room temperature for 72 h. The solvent was eliminated and the crude product was purified by column chromatography using hexane:EtOAc (80:20) as eluent, obtaining the compound (**13d**) (47 mg, 6%). [α]_D_ = +163.41° (*c* = 0.010, CHCl_3_). ^1^H-NMR (CDCl_3_, 400 MHz): δ 1.28 (t, *J* = 7.0 Hz, 3H), 1.48 (d, *J* = 6.8 Hz, 3H), 3.11 (AB system, *J* = 16.4 Hz, 1H), 3.20 (AB system, *J* = 15.6 Hz, 1H), 3.89 (dq, *J* = 6.6, 3.2 Hz, 1H), 4.22 (m, 2H), 5.66 (s, 1H), 7.07–7.44 (m, 13H). ^13^C-NMR (CDCl_3_, 100 MHz): δ 13.8, 16.5, 16.6, 41.3 (d, *J*_C/P_ = 153.75 Hz), 59.3 (d, *J*_C/P_ = 11.7 Hz), 62.0, 65.8, 105.2, 123.0, 123.1, 125.9, 127.7, 127.8, 128.2, 128.3, 128.9, 129.6, 129.7, 131.3, 131.8, 132.1, 140.0, 141.8. ^31^P-NMR (CDCl_3_, 80.95 MHz): δ 21.21. HRMS (CI^+^): *m*/*z* calculated for C_24_H_25_ClNO_3_P [M + H] 441.1261; found for [M + H]^+^, *m*/*z* 442.1361.

### 3.6. General Procedure for the Preparation of 1,4,2-Oxazaphosphepines (**15**), (**16**), (**17**) and (**18**)

Under anhydrous conditions, the corresponding benzoxazine dissolved in dry dichloromethane was treated with dichlorophenylphosphine followed by the slow addition of triethylamine, and the reaction mixture was stirred at room temperature for 72 h. After this time, the solvent was evaporated under reduced pressure, and the residue was treated with a minimum amount of water and extracted with ethyl acetate. The combined organic layers were dried over anhydrous Na_2_SO_4_, evaporated under reduced pressure, and the crude product was purified by column chromatography.

#### 3.6.1. Synthesis of 1,4,2-Oxazaphosphepine 2-oxide (**16a**)

The benzoxazine (**8a**) 1.0 g (4.4 mmol) was reacted at room temperature with dichlorophenylphosphine 0.78 g, 0.6 mL, (4.4 mmol) and triethylamine 0.89 g, 1.22 mL, (8.8 mmol) in dichloromethane (25 mL). The solvent was evaporated under reduced pressure and the crude was purified by column chromatography using a mixture of hexane:EtOAc (8:2), obtaining the compound (**16a**) (110 mg, 7%) as a white solid, mp = 204–206 °C.

*(2S)-2-Phenyl-4-[(1*ʹ*S)-1-phenylethyl]-2,3,4,5-tetrahydro-1,4,2-benzoxazaphosphepine 2-oxide* (**16a**). [α]_D_ = +22.48° (*c* = 0.010, CHCl_3_). ^1^H-NMR (CDCl_3_, 400 MHz): δ 1.46 (d, *J* = 6.4 Hz, 3H), 3.51 (AB system, *J* = 15.2 Hz, 1H), 3.75 (ABX system, *J* = 15.2, 3.2 Hz, 1H), 3.81 (AB system, *J* = 14.4 Hz, 1H), 3.99 (dq, *J* = 7.0, 4.0 Hz, 1H), 4.09 (ABX system, *J* = 14.8, 1.6 Hz, 1H), 6.75–8.02 (m, 14H). ^13^C-NMR (CDCl_3_, 100 MHz): δ 21.7, 53.4 (d, *J*_C/P_ = 89.5 Hz), 55.3, 61.3, 122.4 (d, *J*_C/P_ = 3.0 Hz), 125.1, 127.4, 127.6, 128.6, 128.7, 128.8, 129.7, 130.7, 131.4, 131.5, 131.7, 133.0, 144.3, 150.1 (d, *J*_C/P_ = 6.1 Hz). ^31^P-NMR (CDCl_3_, 80.95 MHz): δ 34.87. HRMS (CI^+^): *m*/*z* calculated for C_22_H_22_NO_2_P [M + H] 363.1388; found for [M + H]^+^, *m*/*z* 364.1454.

#### 3.6.2. Synthesis of 1,4,2-Oxazaphosphepine 2-oxide (**15b**) and (**16b**)

The benzoxazine (**8b**) 0.85 g (3.4 mmol) was reacted at room temperature with dichlorophenylphosphine 0.6 g, 0.46 mL, (3.4 mmol) and triethylamine 0.68 g, 0.94 mL, (6.8 mmol) in dichloromethane (25 mL). The solvent was evaporated under reduced pressure and the crude was analyzed by ^31^P-NMR, observing the two diastereoisomers with a 48:52 ratio, which was purified by column chromatography using a mixture of hexane:EtOAc (8:2), obtaining the compound (**16b**) (190 mg, 15%) as a white solid mp = 168–172 °C. The compound (**16b**) was recrystallized from dichloromethane-hexane to give a crystal for X-ray studies. The diastereoisomer (**15b**) was obtained as unstable colorless oil (14 %) and only the ^1^H-NMR spectrum was obtained.

*(2R)-2-Phenyl-(5S)-5-methyl-4-[(1ʹS)-1-phenylethyl]-2,3,4,5-tetrahydro-1,4,2-benzoxazaphosphepine 2-oxide* (**15b**). [α]_D_ = +36.17° (*c* = 0.010, CHCl_3_). ^1^H-NMR (CDCl_3_, 200 MHz): δ 1.48 (d, *J* = 6.8 Hz, 3H), 1.51 (d, *J* = 7.6 Hz, 3H), 3.58 (ABX system, *J* = 15.4, 10.6 Hz, 1H), 3.67 (ABX system, *J* = 15.8, 7.8 Hz, 1H), 4.22 (q, *J* = 6.8 Hz, 1H), 4.36 (q, *J* = 7.2 Hz, 1H), 6.90–7.84 (m, 14H).

*(2S)-2-Phenyl-(5S)-5-methyl-4-[(1ʹS)-1-phenylethyl]-2,3,4,5-tetrahydro-1,4,2-benzoxazaphosphepine 2-oxide* (**16b**). [α]_D_ = +71.55° (*c* = 0.010, CHCl_3_). ^1^H-NMR (CDCl_3_, 200 MHz): δ 1.48 (d, *J* = 6.6 Hz, 3H), 1.58 (d, *J* = 7.0 Hz, 3H), 3.56 (AB system, *J* = 16 Hz, 1H), 3.67 (AB system, *J* = 16.4 Hz, 1H), 3.91 (q, *J* = 6.9 Hz, 1H), 4.04 (q, *J* = 6.5 Hz, 1H), 6.8–7.94 (m, 14H). ^13^C-NMR (CDCl_3_, 50 MHz): δ 20.5, 20.6, 45.1 (d, *J*_C/P_ = 87.9 Hz), 60.2, 60.6, 123.6, 125.3, 127.0, 127.3, 128.4, 128.5, 128.8, 129.4, 131.1, 131.5 (d, *J*_C/P_ = 9.1 Hz), 132.8, 135.2, 144.9, 148.7 (d, *J*_C/P_ = 7.6 Hz). ^31^P-NMR (CDCl_3_, 161.8 MHz): δ 37.23. HRMS (CI^+^): *m*/*z* calculated for C_23_H_24_NO_2_P [M + H] 377.1545; found for [M + H]^+^, *m*/*z* 378.1612.

#### 3.6.3. Synthesis of 1,4,2-Oxazaphosphepine 2-oxide (**15c**) and (**16c**)

The benzoxazine (**8c**) 0.30 g (0.9 mmol) was reacted at room temperature with dichlorophenylphosphine 0.17 g, 0.13 mL, (0.9 mmol) and triethylamine 0.19 g, 0.26 mL, (1.9 mmol) in dichloromethane (5 mL). The solvent was evaporated under reduced pressure and the crude product was purified by column chromatography using a mixture of hexane:EtOAc (80:20) as eleuent, obtaining the compounds (**15c**) as a colorless high viscosity oil (7%) and (**16c**) as a white solid (45%) mp = 166–172 °C.

*(2R,S)-2-Phenyl-(5S)-5-phenyl-4-[(1ʹS)-1-phenylethyl]-2,3,4,5-tetrahydro-1,4,2-benzoxazaphosphepine 2-oxide* (**15c**). [α]_D_ = +88.4° (*c* = 0.010, CHCl_3_). ^1^H-NMR (CDCl_3_, 200 MHz): δ 1.29 (d, *J* = 6.8 Hz, 3H), 3.35 (ABX system, *J* = 16.4, 14.6 Hz, 1H), 3.49 (ABX system, *J* = 16.4, 6.4 Hz, 1H), 4.09 (dq, *J* = 6.8, 6.4 Hz, 1H), 5.29 (s, 1H), 6.70–7.59 (m, 19H). ^13^C-NMR (CDCl_3_, 50 MHz): δ 15.9, 29.8, 45.3 (d, *J*_C/P_ = 95.2), 60.0 (d, *J* = 7.3 Hz), 69.1, 123.6, 123.7, 125.8, 127.6, 127.7, 127.9, 128.5, 128.6, 128.7, 128.8, 129.4, 131.4, 131.5, 132.0, 132.8, 132.9, 142.4 (d, *J*_C/P_ = 99.5 Hz). ^31^P-NMR (CDCl_3_, 161.8 MHz): δ 36.74. HRMS (CI^+^): *m*/*z* calculated for C_23_H_24_NO_2_P [M + H] 439.1701; found for [M + H]^+^, *m*/*z* 440.1774.

*(2R,S)-2-Phenyl-(5S)-5-phenyl-4-[(1ʹS)-1-phenylethyl]-2,3,4,5-tetrahydro-1,4,2-benzoxazaphosphepine 2-oxide* (**16c**). (0.19 g, 45%) [α]_D_ = +146.78° (*c* = 0.010, CHCl_3_). ^1^H-NMR (CDCl_3_, 200 MHz): δ 1.56 (d, *J* = 6.4 Hz, 3H), 3.40 (AB system, *J* = 16.4 Hz, 1H), 3.58 (AB system, *J* = 16.4 Hz, 1H), 4.14 (q, *J* = 6.0 Hz, 1H), 5.05 (s, 1H), 7.12–7.57 (m, 19H). ^13^C-NMR (CDCl_3_, 50 MHz): δ 21.3, 20.6, 45.9 (d, *J*_C/P_ = 84.9 Hz), 60.3, 68.5, 123.9, 125.6, 127.4, 127.5, 127.6, 128.2, 128.6, 128.7, 130.2, 131.3, 131.4, 132.7, 132.9, 149.22. ^31^P-NMR (CDCl_3_, 161.8 MHz): δ 32.57. HRMS (CI^+^): *m*/*z* calculated for C_23_H_24_NO_2_P [M + H] 439.1701; found for [M + H]^+^, *m*/*z* 440.1783.

#### 3.6.4. Synthesis of 1,4,2-Oxazaphosphepine 2-oxide (**15d**) and (**16d**)

The benzoxazine (**8d**) 1.0 g (2.9 mmol) was reated at room temperature with dichlorophenylphosphine 0.5 g, 0.39 mL, (2.9 mmol) and triethylamine 0.57 g, 0.80 mL, (5.7 mmol) in dichloromethane (15 mL). The solvent was evaporated under reduced pressure and the crude product was purified by column chromatography using a mixture of hexane:EtOAc (80:20) as eluent, obtaining the compounds (**15d**) (50 mg, 4%) as an orange solid, mp = 65–68 °C and (**16d**) (50 mg, 4%) as a white solid mp = 220–224 °C with a diastereoisomeric ratio 50:50.

*(2R)-2-Phenyl-(5R)-5-(2-chlorophenyl)-4-[(1ʹS)-1-phenylethyl]-2,3,4,5-tetrahydro-1,4,2-benzoxazaphosphepine 2-oxide* (**15d**). [α]_D_ = +116.74° (*c* = 0.0036, CHCl_3_). ^1^H-NMR (CDCl_3_, 200 MHz): δ 1.44 (d, *J* = 7.0 Hz, 3H), 3.15 (ABX system, *J* = 15.2, 15.2 Hz, 1H), 3.43 (ABX system, *J* = 16.1, 9.9 Hz, 1H), 4.02 (q, *J* = 6.6 Hz, 1H), 5.90 (s, 1H), 6.70–8.54 (m, 18H). ^13^C-NMR (CDCl_3_, 50 MHz): δ 12.6, 43.7 (d, *J*_C/P_ = 104.9 Hz), 59.5, 65.5, 124.2, 126.0, 127.7, 128.0, 128.3, 128.5, 128.9, 129.3, 131.7, 131.9, 132.7, 141.2, 148.3. ^31^P-NMR (CDCl_3_, 81 MHz): δ 38.60. HRMS (CI^+^): *m*/*z* calculated for C_28_H_25_ClNO_2_P [M + H] 473.1311; found for [M + H]^+^, *m*/*z* 474.1437.

*(2S)-2-Phenyl-(5R)-5-(2-chlorophenyl)-4-[(1ʹS)-1-phenylethyl]-2,3,4,5-tetrahydro-1,4,2-benzoxazaphosphepine 2-oxide* (**16d**). [α]_D_ = +223.83° (*c* = 0.0072, CHCl_3_). ^1^H-NMR (CDCl_3_, 400 MHz): δ 1.54 (d, *J* = 6.8 Hz, 3H), 3.37 (ABX system, *J* = 16.0, 6.4 Hz, 1H), 3.42 (ABX system, *J* = 16.0, 8.4 Hz, 1H), 3.93 (bs, 1H), 5.85 (s, 1H), 6.94–8.11 (m, 18H). ^13^C-NMR (CDCl_3_, 50 MHz): δ 11.4, 44.0 (d, *J*_C/P_ = 107.9 Hz), 58.8, 66.9, 124.7, 126.3, 127.3, 127.7, 127.9, 128.1, 128.3, 128.5, 129.0, 129.8, 130.0, 130.7, 131.2, 131.4, 132.1, 132.4, 133.3, 140.9, 147.2. ^31^P-NMR (CDCl_3_, 81 MHz): δ 42.36. HRMS (CI^+^): *m*/*z* calculated for C_28_H_25_ClNO_2_P [M + H] 473.1311; found for [M + H]^+^, *m*/*z* 474.1390.

#### 3.6.5. Synthesis of 1,4,2-Oxazaphosphepine 2-oxide (**17c**) and (**18c**)

The benzoxazine (**9c**) 0.25 g (0.8 mmol) was reacted at room temperature with dichlorophenylphosphine 0.14 g, 0.10 mL, (0.8 mmol) and triethylamine 0.16 g, 0.22 mL, (1.6 mmol) in dichloromethane (5 mL). The solvent was evaporated under reduced pressure and the crude product was purified by column chromatography using a mixture of hexane:EtOAc (80:20) as eluent, obtaining the compounds (**17c**) (18 mg, 5%) as a white solid mp = 60–65 °C, and (**18c**) which is unstable in solution, (60 mg, 17%) as a white solid mp = 193–195 °C.

*(2S)-2-Phenyl-(5R)-5-phenyl-4-[(1ʹS)-1-phenylethyl]-2,3,4,5-tetrahydro-1,4,2-benzoxazaphosphepine 2-oxide* (**17c**). ^1^H-NMR (CDCl_3_, 200 MHz): δ 1.30 (d, *J* = 6.6 Hz, 3H), 3.36 (ABX system, *J* = 16.0, 12.2 Hz, 1H), 3.52 (ABX system, *J* = 16.1, 5.1 Hz, 1H), 4.11 (dq, *J* = 7.0, 2.2 Hz, 1H), 5.30 (s, 1H), 6.91–7.63 (m, 19H). ^13^C-NMR (CDCl_3_, 50 MHz): δ 15.9, 45.3 (d, *J*_C/P_ = 95.5 Hz), 59.9, 69.1, 123.5, 125.7, 127.6, 127.8, 128.4, 128.6, 128.6, 129.3, 131.3, 131.5, 132.0, 132.7, 141.8, 142.7. ^31^P-NMR (CDCl_3_, 80.95 MHz): δ 36.7. HRMS (CI^+^): *m*/*z* calculated for C_23_H_24_NO_2_P [M] 439.1701; found for [M + H]^+^, *m*/*z* 439.1772.

*(2R)-2-Phenyl-(5R)-5-phenyl-4-[(1ʹS)-1-phenylethyl]-2,3,4,5-tetrahydro-1,4,2-benzoxazaphosphepine 2-oxide* (**18c**). [α]_D_ = −95.35° (*c* = 0.010, CHCl_3_). ^1^H-NMR (CDCl_3_, 400 MHz): δ 1.20 (d, *J* = 6.6 Hz, 3H), 3.04 (ABX system, *J* = 16.1, 4.7 Hz, 1H), 3.48 (ABX system, *J* = 16.1, 3.7 Hz, 1H), 4.16 (q, *J* = 7.0 Hz, 1H), 5.35 (s, 1H), 7.14–7.86 (m, 19H). ^13^C-NMR (CDCl_3_, 50 MHz): δ 21.3, 46.1 (d, *J*_C/P_ = 98.7 Hz), 60.3, 69.7, 124.3, 126.1, 127.7, 127.8, 128.2, 128.5, 128.6, 128.9, 130.2, 131.9, 132.0, 132.1, 133.0, 142.3, 148.0. ^31^P-NMR (CDCl_3_, 161.8 MHz): δ 37.84. HRMS (CI^+^): *m*/*z* calculated for C_23_H_24_NO_2_P [M] 439.1701; found for [M + H]^+^, *m*/*z* 439.1639.

#### 3.6.6. Synthesis of 1,4,2-Oxazaphosphepine 2-oxide (**17d**) and (**18d**)

The benzoxazine (**9d**) 300 mg (0.9 mmol) was reacted at room temperature with dichlorophenylphosphine 150 mg, 0.11 mL, (0.85 mmol) and triethylamine 170 mg, 0.24 mL, (1.7 mmol) in dichloromethane (10 mL). The solvent was evaporated under reduced pressure and the crude product was purified by column chromatography using a mixture of hexane:EtOAc (80:20) as eluent, obtaining (430 mg, 9%) of diastereoisomeric mixture as high viscosity oil, the two compounds were identified by ^1^H- and ^31^P-NMR with a 16:84 diastereoisomeric ratio, which the separation was not possible.

*(2R,S)-2-Phenyl-(5S)-5-(2-chlorophenyl)-4-[(1ʹS)-1-phenylethyl]-2,3,4,5-tetrahydro-1,4,2-benzoxazaphosphepine 2-oxide* (**17d**) *and* (**18d**). The asterisk denotes the minor diastereoisomer.^1^H-NMR (CDCl_3_, 200 MHz): δ 1.06* (d, *J* = 7.0 Hz, 3H), 1.49 (d, *J* = 7.0 Hz, 3H), 2.99* (ABX system, *J* = 16.2, 4.2 Hz, 1H), 3.25 (ABX system, *J* = 16.3, 5.8 Hz, 1H), 3.42* (ABX system, *J* = 16.4, 5.4 Hz, 1H), 3.56 (ABX system, *J* = 16.5, 4.4 Hz, 1H), 3.96* (q, *J* = 6.9 Hz, 1H), 3.97 (q, *J* = 6.9 Hz, 1H), 5.76* (s, 1H), 5.85 (s, 1H), 6.72–8.45 (m, 36H). ^31^P-NMR (CDCl_3_, 81 MHz): δ 40.35*, 41.51. HRMS (FAB^+^): *m*/*z* calculated for C_28_H_25_ClNO_2_P [M + H] 473.1311; found for [M + H]^+^, *m*/*z* 474.1389.

## 4. Conclusions

In conclusion, we have developed a method for the diastereoisomeric synthesis of 1,4,2-oxazaphosphephines by nucleophilic addition of dichlorophenylphosphine or trimethyl phosphite to chiral 1,3-benzoxazines, which were easily prepared from chiral *o*-aminophenols. The X-ray analysis shows that these heterocycles adopt a chair and boat conformation. Additionally, these compounds represent an opportunity for more detailed studies and applications.

## Supplementary Materials

Supplementary materials can be accessed at: http://www.mdpi.com/1420-3049/20/08/13794/s1.
