# Supplementary material for: Synthesis of Chiral 1,4,2-Oxazaphosphepines
Source: Molecules. 2015 Jul 29;20(8):13794–813. doi: 10.3390/molecules200813794 (PMC6331794; doi:10.3390/molecules200813794)
Supplement: Supplementary file 1 [file molecules-20-13794-s001.pdf]

Supplementary Material

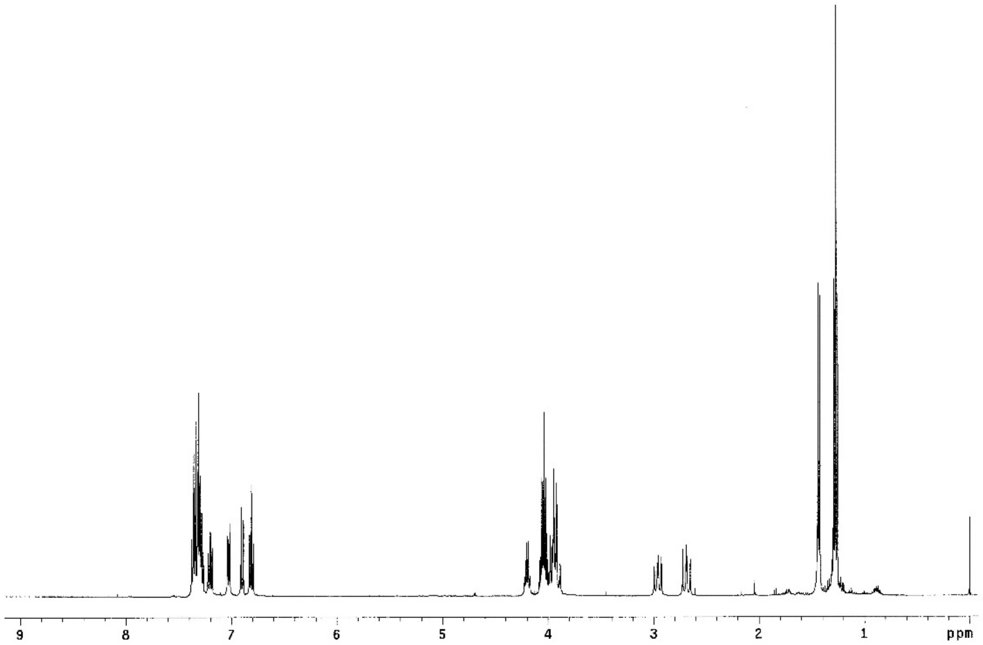

(S)-Diethyl-{[(2-hydroxybenzyl)(1-phenylethyl)amino]methyl}phosphonate (**10a**). ^{1}H-NMR ($CDCl_3$, 400 MHz): δ 1.27 (t, *J* = 6.8 Hz, 3H), 1.42 (d, *J* = 7.2 Hz, 1H), 2.67 (ABX system, *J* = 15.6, 12.4 Hz, 1H), 2.94 (ABX system, *J* = 15.6, 11.6 Hz, 1H), 3.89 (AB system, *J* = 14.0 Hz, 1H), 3.95 (AB system, *J* = 14.0 Hz, 1H), 4.00–4.07 (m, 4H), 4.19 (q, *J* = 7.2 Hz, 1H), 6.78–7.37 (m, 9H).

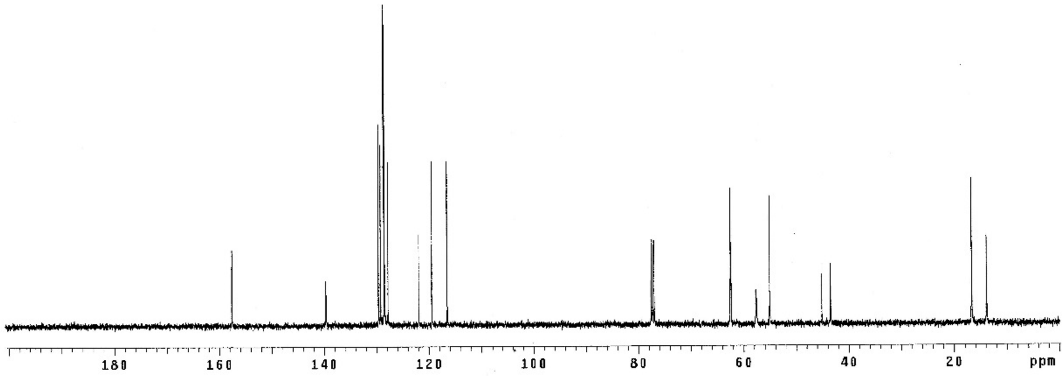

(S)-Diethyl-{[(2-hydroxybenzyl)(1-phenylethyl)amino]methyl}phosphonate (**10a**). ^{13}C-NMR ($CDCl_3$, 100 MHz): δ 13.6, 16.5 (d, $J_{C/P}$ = 5.9 Hz), 44.2 (d, $J_{C/P}$ = 163.9 Hz), 55.0 (d, $J_{C/P}$ = 4.4 Hz), 57.4 (d, $J_{C/P}$ = 11.7 Hz), 62.3 (d, $J_{C/P}$ = 5.9 Hz), 62.4 (d, *J* = 5.9 Hz), 116.5, 119.4, 122.0, 127.8, 128.4, 128.5, 128.6, 129.2, 129.7, 139.7, 157.7.

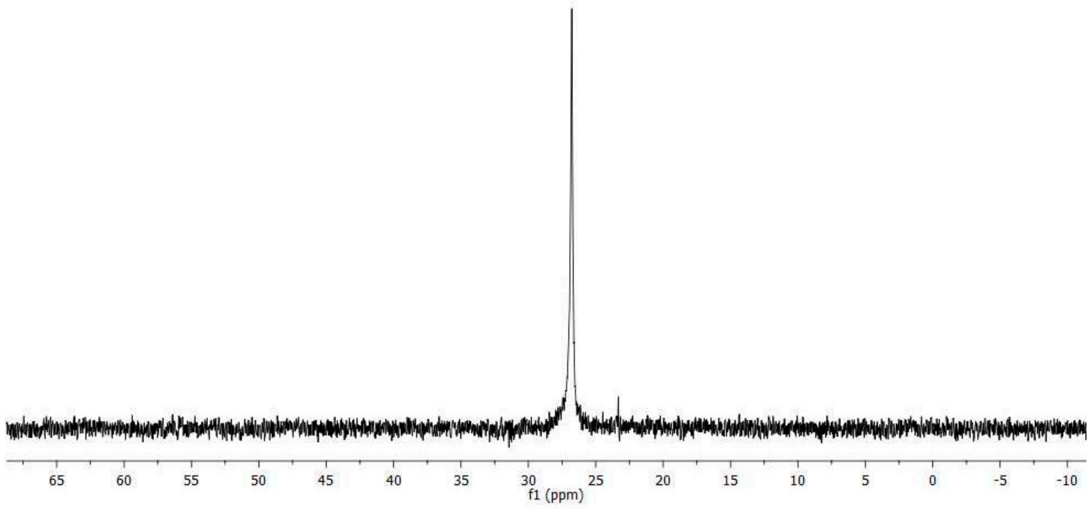

(S)-Diethyl-{[(2-hydroxybenzyl)(1-phenylethyl)amino]methyl}phosphonate (**10a**). ^{31}P-NMR ($CDCl_3$, 80.95 MHz): δ 26.78.

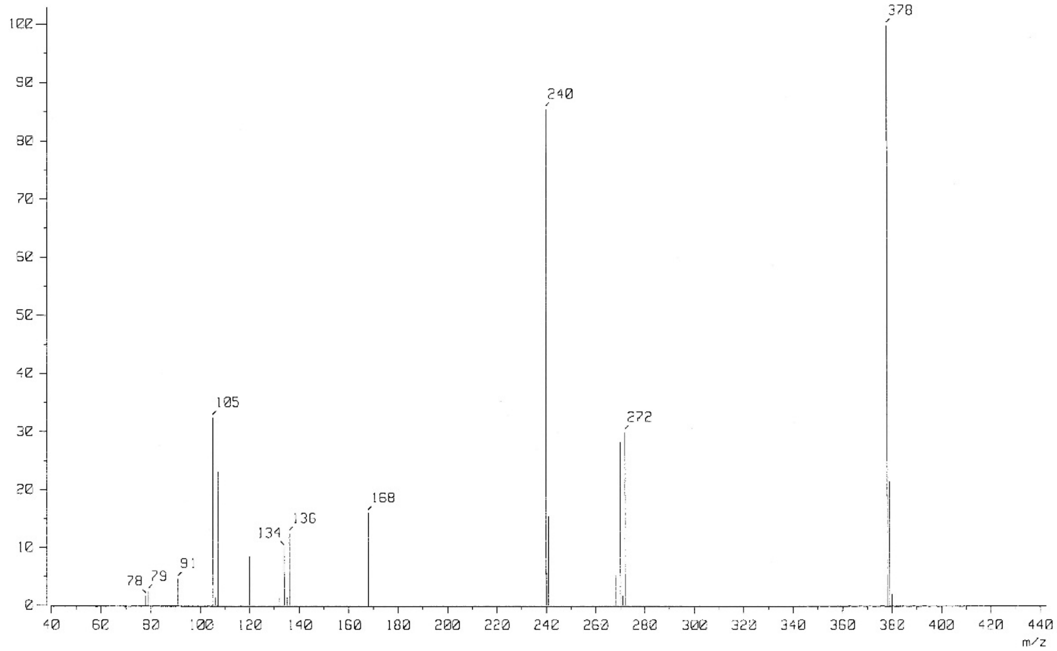

(S)-Diethyl-{[(2-hydroxybenzyl)(1-phenylethyl)amino]methyl}phosphonate (**10a**). HRMS (CI^+): found for $[M + H]^+$, *m/z* 378.1819.

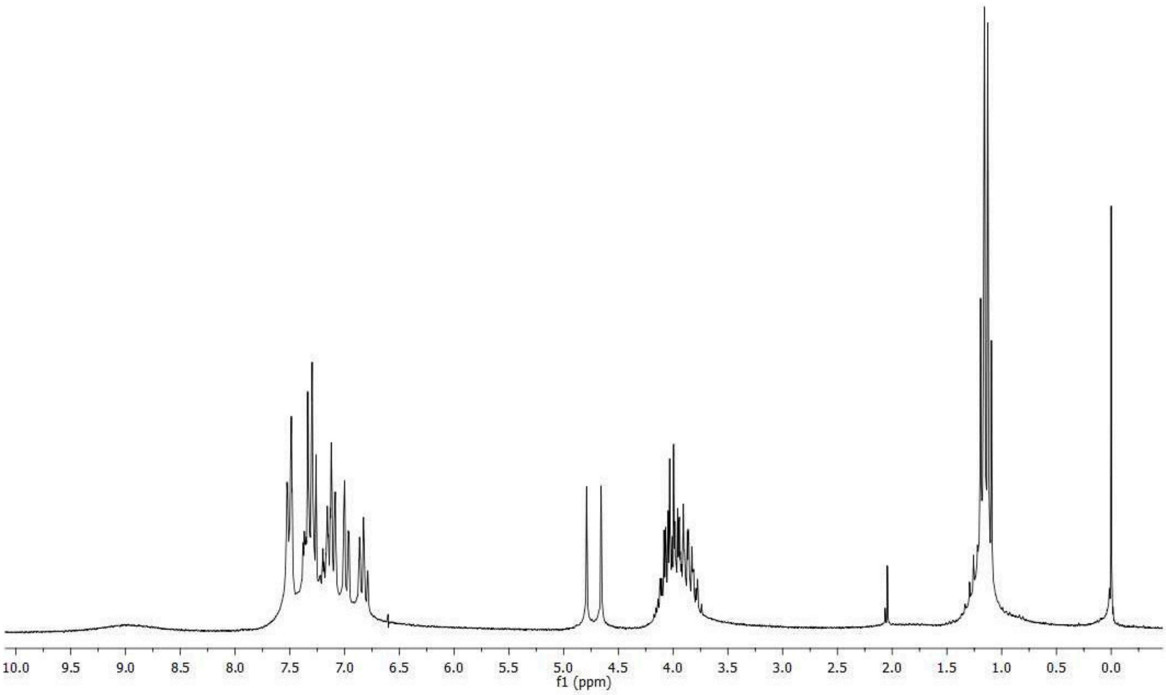

Diethyl-[(2-hydroxyphenyl)(phenyl)methyl]phosphonate (**11c**). ^{1}H-NMR ($CDCl_3$, 400 MHz): δ 1.12 (t, J = 7.0 Hz, 3H), 1.15 (t, J = 6.8 Hz, 3H), 3.86–4.08 (m, 4H), 4.72 (AB system, $J_{H/P}$ = 26.6 Hz, 1H), 7.00–7.52 (m, 9H), 8.89 (br, 1H).

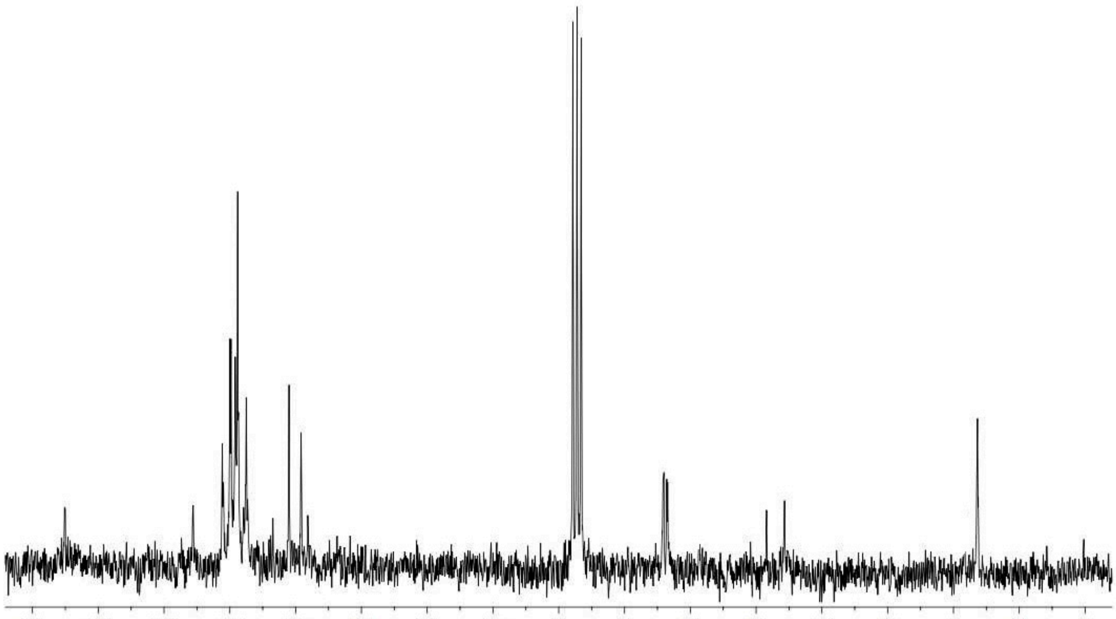

Diethyl-[(2-hydroxyphenyl)(phenyl)methyl]phosphonate (**11c**). ^{13}C-NMR ($CDCl_3$, 100 MHz): δ 16.3, 47.0 (d, $J_{C/P}$ = 136.15 Hz), 63.5 (d, $J_{C/P}$ = 7.0 Hz), 64.0 (d, $J_{C/P}$ = 7.4 Hz), 118.1, 119.1, 121.0, 127.3, 127.5, 128.6, 128.8, 129.1, 129.8 (d, $J_{C/P}$ = 8.1 Hz), 131.0 (d, $J_{C/P}$ = 7.7 Hz), 136.5 (d, $J_{C/P}$ = 4.35 Hz), 155.0 (d, $J_{C/P}$ = 5.85 Hz).

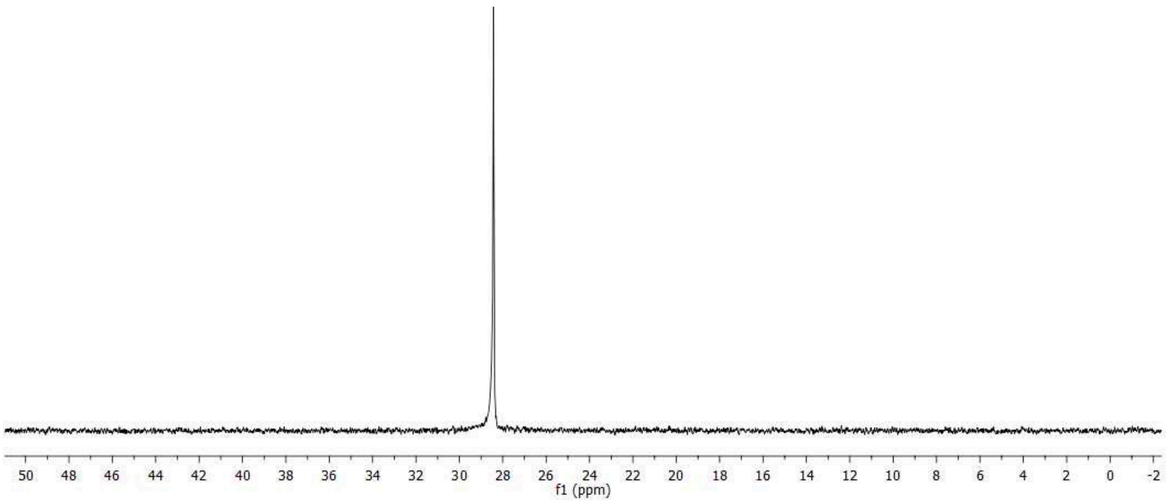

Diethyl-[(2-hydroxyphenyl)(phenyl)methyl]phosphonate (**11c**). ^{31}P-NMR ($CDCl_3$, 80.95 MHz): δ 28.43.

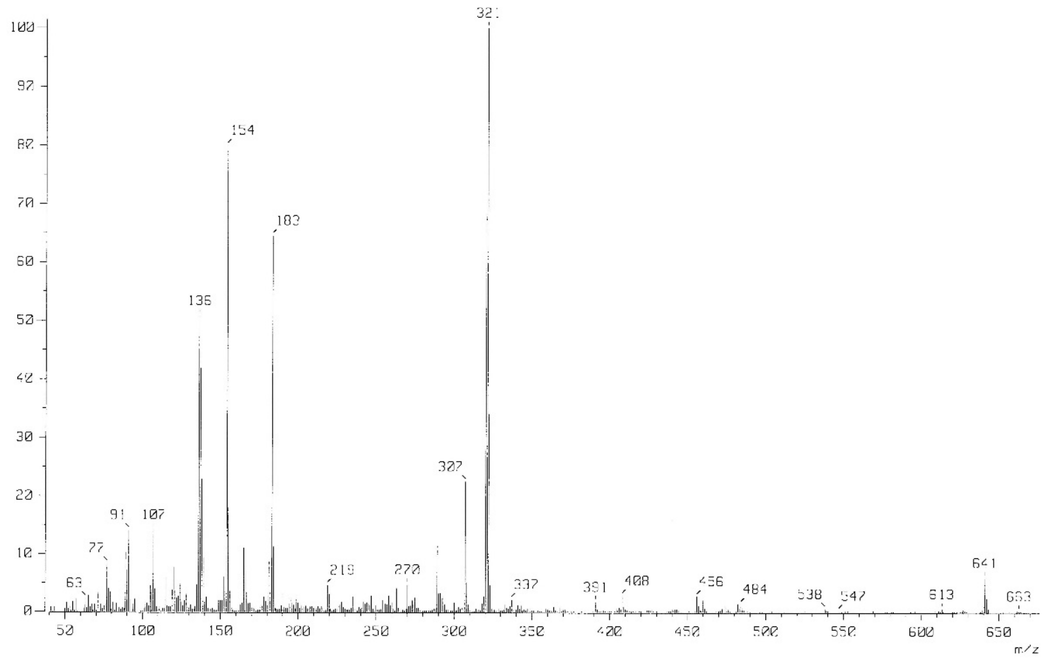

Diethyl-[(2-hydroxyphenyl)(phenyl)methyl]phosphonate (**11c**). HRMS (CI^+): found for $[M + H]^+$, *m/z* 321.1033.

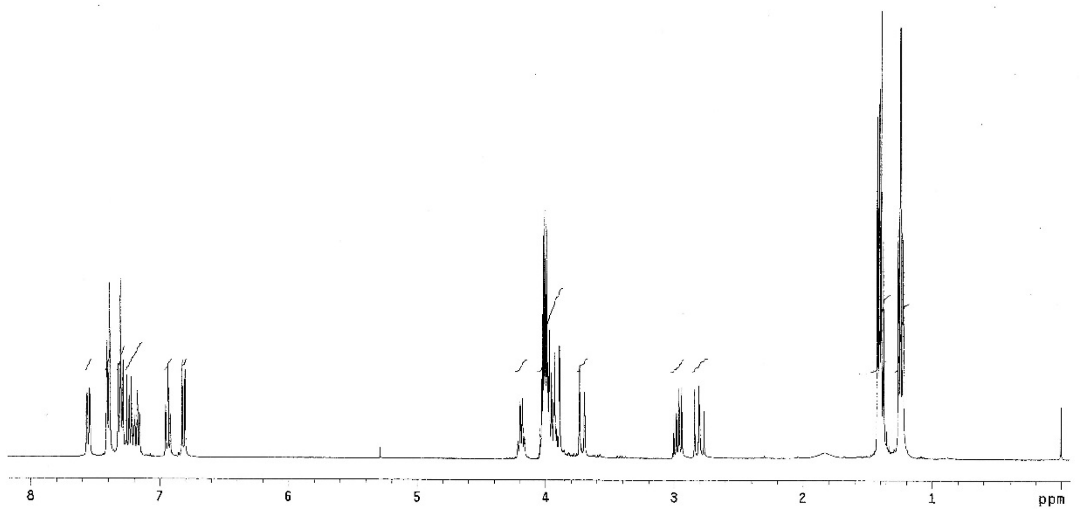

(S)-2,2,2-Triethoxy-4-(1-phenylethyl)-2,3,4,5-tetrahydro-1,4,2λ^5-benzoxazaphosphepine (**12a**). ^{1}H-NMR ($CDCl_3$, 400 MHz): δ 1.24 (t, J = 6.8 Hz, 3H), 1.25 (t, J = 6.8 Hz, 3H), 1.39 (t, J = 6.8 Hz, 3H), 1.41 (d, J = 7.2 Hz, 3H), 2.80 (ABX system, $J_{H/P}$ = 15.4, 12.6 Hz, 1H), 2.97 (ABX system, $J_{H/P}$ = 15.6, 8.4 Hz, 1H), 3.71 (AB system, J = 14.8 Hz, 1H), 3.91 (AB system, J = 14.8 Hz, 1H), 3.96 (q, J = 7.2 Hz, 2H), 4.00 (q, J =6.8 Hz, 2H), 4.01(q, J = 7.0 Hz, 2H), 4.19 (q, J = 6.8 Hz, 1H) 6.80–7.56 (m, 9H).

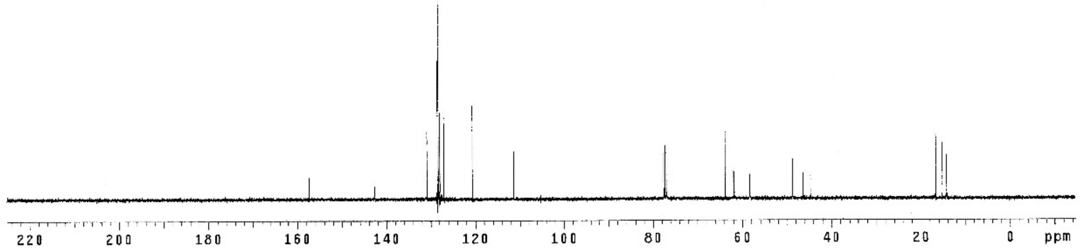

(S)-2,2,2-Triethoxy-4-(1-phenylethyl)-2,3,4,5-tetrahydro-1,4,2λ^5-benzoxazaphosphepine (**12a**). ^{13}C-NMR ($CDCl_3$, 100 MHz): δ 14.1, 15.1, 16.60, 16.66, 45.5 (d, $J_{C/P}$ = 162.5 Hz), 48.6 (d, $J_{C/P}$ = 7.3 Hz), 58.3 (d, $J_{C/P}$ = 10.2 Hz), 61.7 (d, $J_{C/P}$ = 7.3 Hz), 61.8 (d, J = 7.3 Hz), 63.7, 111.3, 120.5, 126.9, 127.8, 128.1, 128.4, 130.7, 142.6, 157.2.

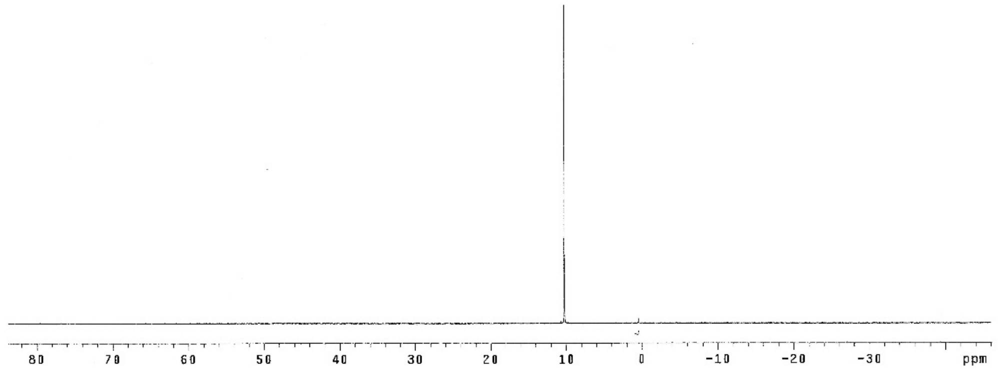

(S)-2,2,2-Triethoxy-4-(1-phenylethyl)-2,3,4,5-tetrahydro-1,4,2λ⁵-benzoxazaphosphepine (**12a**). ^{31}P-NMR ($CDCl_3$, 80.95 MHz): δ 10.24.

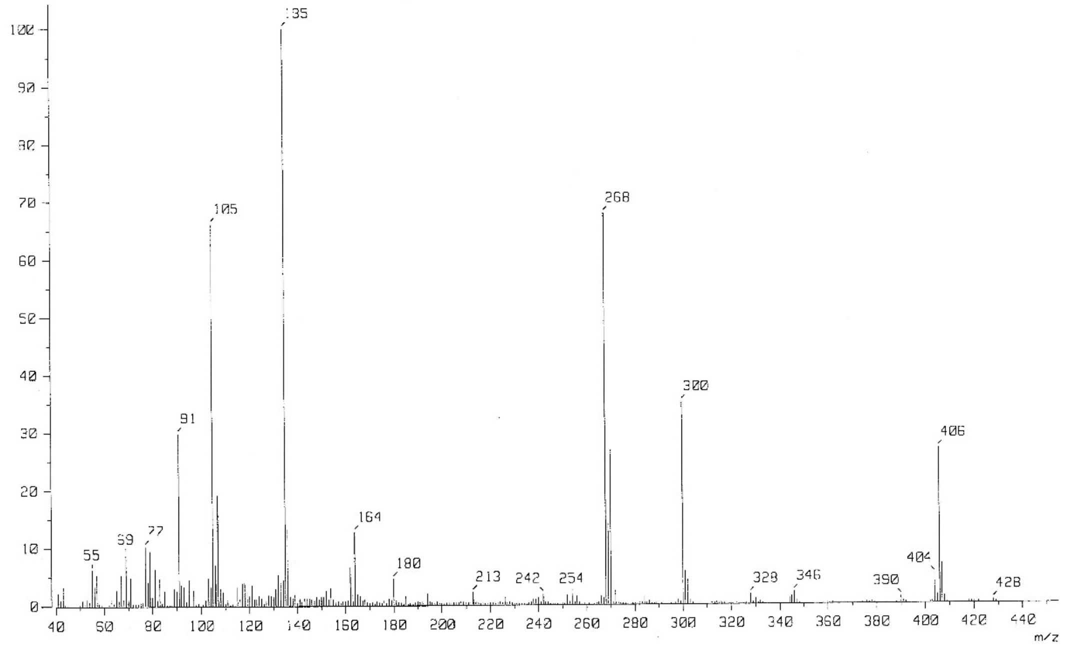

(S)-2,2,2-Triethoxy-4-(1-phenylethyl)-2,3,4,5-tetrahydro-1,4,2λ⁵-benzoxazaphosphepine (**12a**). HRMS (CI^+): found for $[M + H]^+$, *m/z* 406.2128.

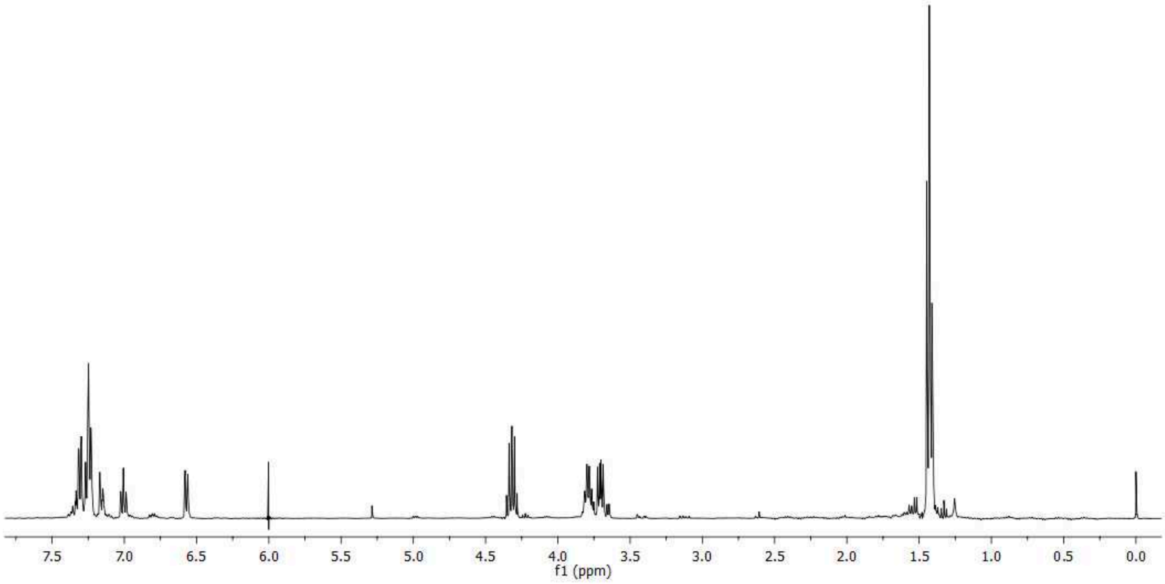

(2R,S)-2-Ethoxy-(5S)-5-methyl-4-[(1'S)-1-phenylethyl]-2,3,4,5-tetrahydro-1,4,2-benzoxazaphosphepine 2-oxide (**13b**). ^{1}H-NMR ($CDCl_3$, 400 MHz): δ 1.41 (d, J = 6.8 Hz, 3H), 1.43 (d, J = 6.8 Hz, 3H), 1.43 (t, J = 6.8 Hz, 3H), 3.67 (ABX system, $J_{H/P}$ = 16.4, 5.8 Hz, 1H), 3.74 (ABX system, $J_{H/P}$ = 16.4, 6.0 Hz, 1H), 3.79 (dq, J = 7.2, 5.3 Hz, 2H), 4.31 (q, J = 7.2 Hz, 1H), 4.33 (q, J = 7.2 Hz, 1H), 6.56–7.35 (m, 9H).

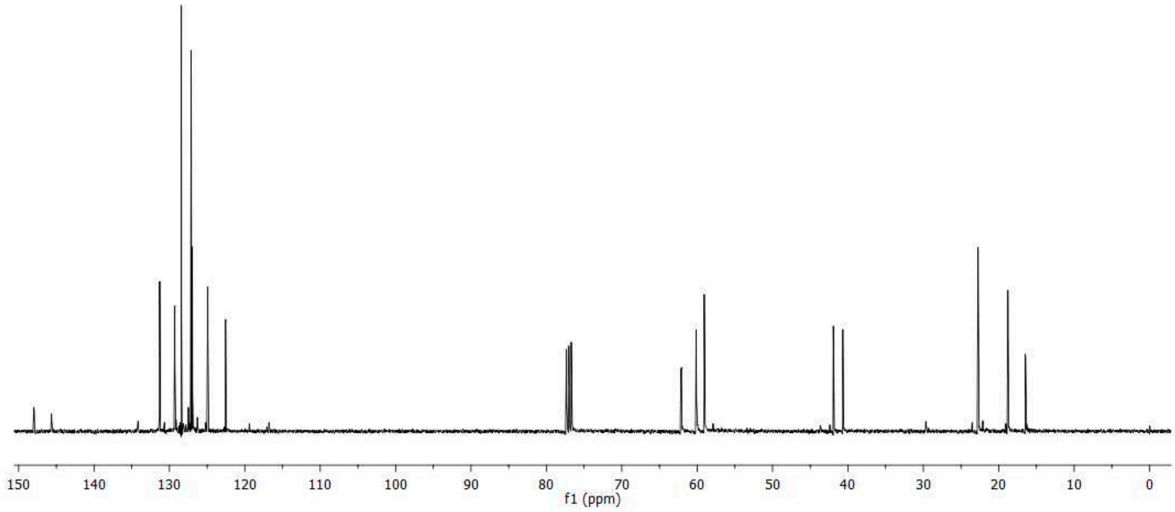

(2R,S)-2-Ethoxy-(5S)-5-methyl-4-[(1'S)-1-phenylethyl]-2,3,4,5-tetrahydro-1,4,2-benzoxazaphosphepine 2-oxide (**13b**). ^{13}C-NMR ($CDCl_3$, 100 MHz): δ 16.4, 16.5, 18.7, 22.7, 41.3 (d, $J_{C/P}$ = 125.9 Hz), 59.0, 60.1, 62.1 (d, $J_{C/P}$ = 8.8 Hz), 122.5, 122.6, 124.9, 126.9, 127.1, 128.4, 129.3, 131.3, 134.1, 145.6, 147.9.

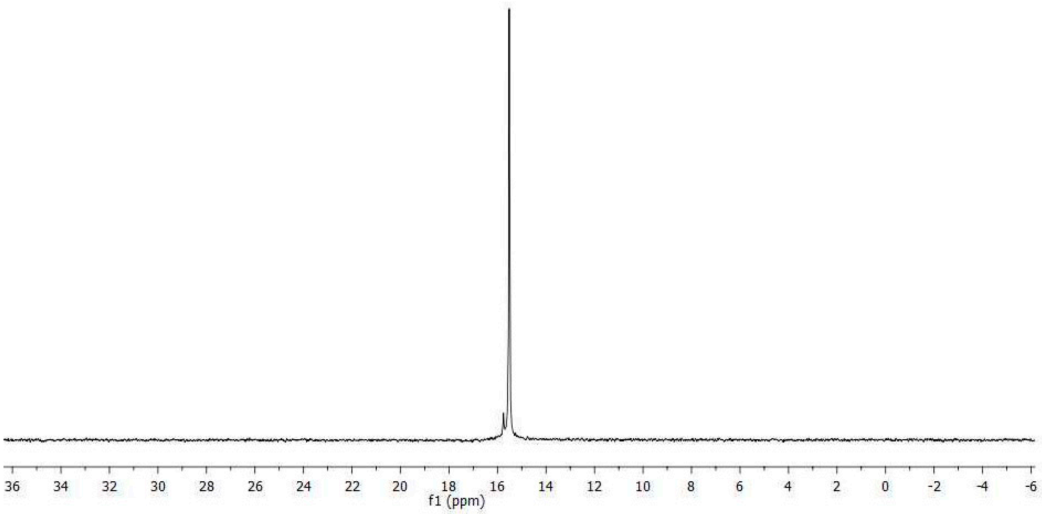

(2R,S)-2-Ethoxy-(5S)-5-methyl-4-[(1'S)-1-phenylethyl]-2,3,4,5-tetrahydro-1,4,2-benzoxazaphosphepine 2-oxide (**13b**). ^{31}P-NMR ($CDCl_3$, 80.95 MHz): δ 15.52.

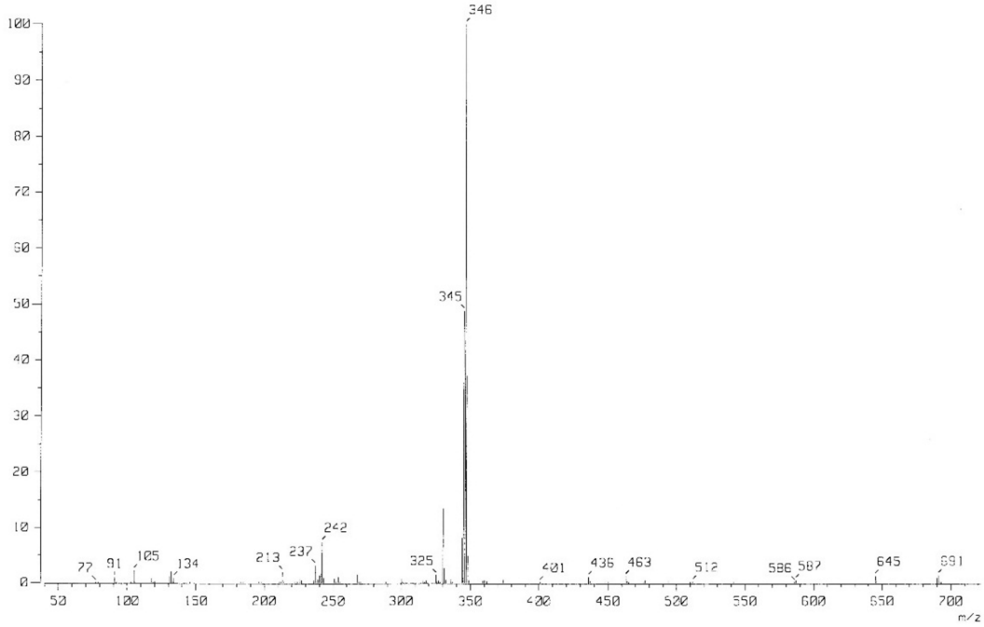

(2R,S)-2-Ethoxy-(5S)-5-methyl-4-[(1'S)-1-phenylethyl]-2,3,4,5-tetrahydro-1,4,2-benzoxazaphosphepine 2-oxide (**13b**). HRMS (CI^+): found for $[M + H]^+$, *m/z* 346.1557.

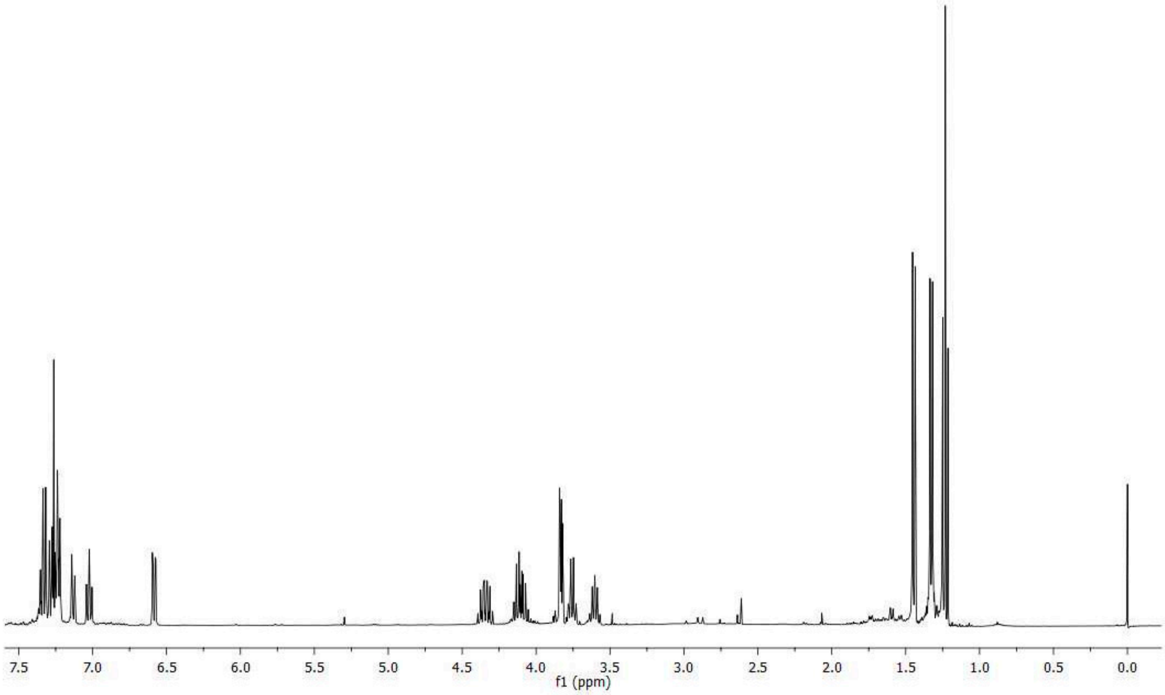

(2R,S)-2-Ethoxy-(5S)-5-methyl-4-[(1'S)-1-phenylethyl]-2,3,4,5-tetrahydro-1,4,2-benzoxazaphosphepine 2-oxide, (**14b**). ^{1}H-NMR ($CDCl_3$, 400 MHz): δ 1.23 (d, J = 7.0 Hz, 3H), 1.32 (d, J = 6.8 Hz, 3H), 1.44 (t, J = 7.2 Hz, 3H), 3.60 (dq, J = 6.8, 6.8 Hz, 1H), 3.75 (q, 7.2 Hz, 1H), 3.82 (ABX system, $J_{H/P}$ = 16.8, 1.6 Hz, 1H), 3.85 (ABX system, $J_{H/P}$ = 16.8, 3.6 Hz, 1H), 4.05–4.15 (m, 2H), 4.29–4.39 (m, 2H), 6.57–7.35 (m, 9H).

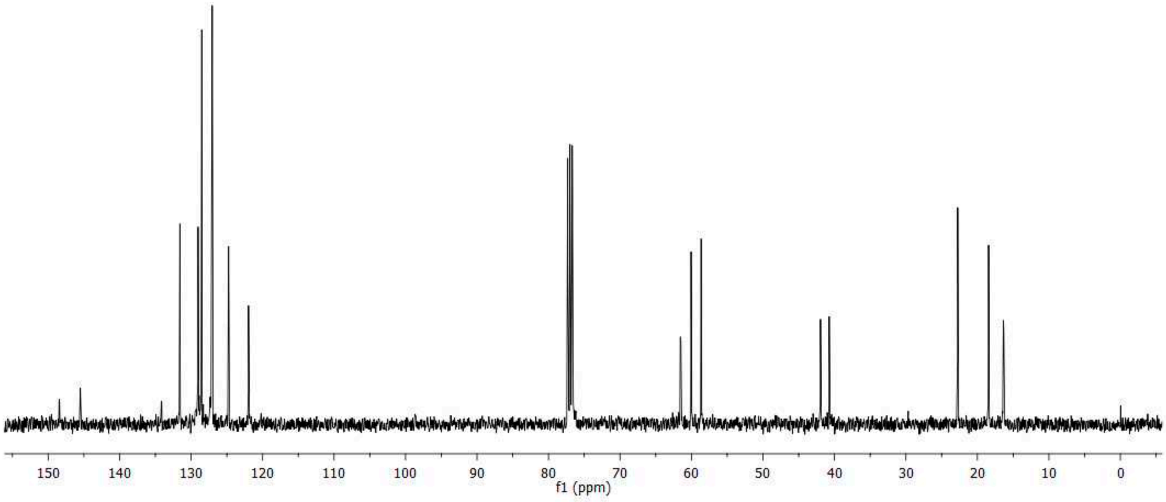

(2R,S)-2-Ethoxy-(5S)-5-methyl-4-[(1'S)-1-phenylethyl]-2,3,4,5-tetrahydro-1,4,2-benzoxazaphosphepine 2-oxide, (**14b**). ^{13}C-NMR ($CDCl_3$, 100 MHz): δ 16.3, 16.4, 18.4, 22.7, 41.3 (d, $J_{C/P}$ = 123.0 Hz), 58.6, 60.0, 61.5 (d, $J_{C/P}$ = 8.8 Hz), 121.9, 122.0, 124.7, 127.0, 127.1, 128.5, 129.0, 131.5, 134.1, 145.5, 148.4.

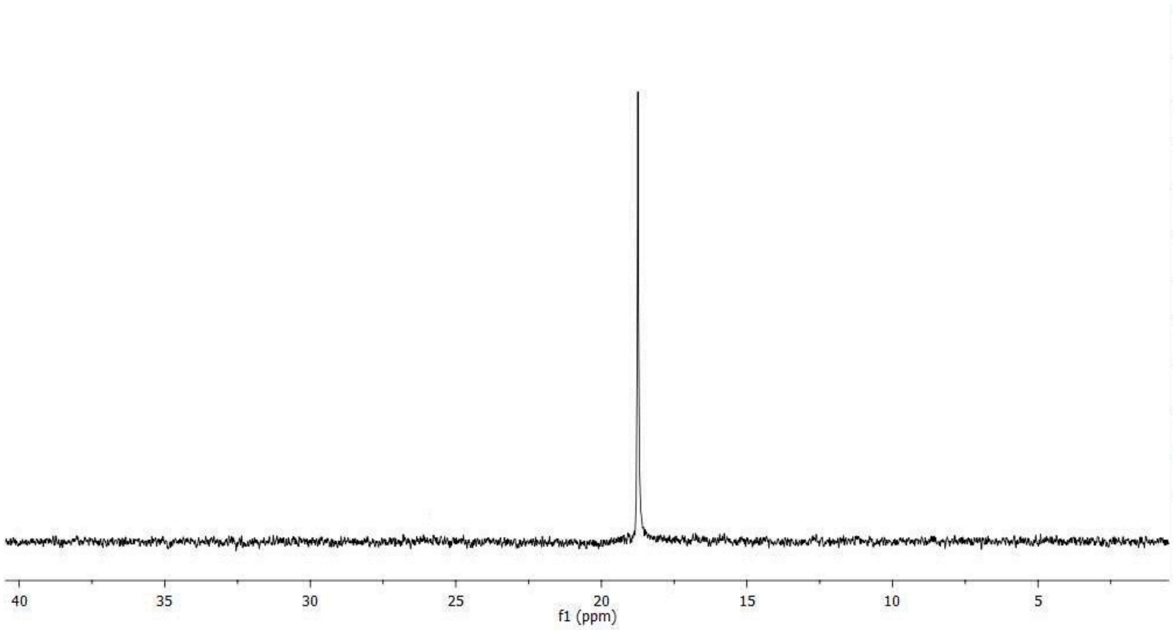

(2R,S)-2-Ethoxy-(5S)-5-methyl-4-[(1'S)-1-phenylethyl]-2,3,4,5-tetrahydro-1,4,2-benzoxazaphosphepine 2-oxide, (**14b**). ^{31}P-NMR ($CDCl_3$, 80.95 MHz): δ 18.75.

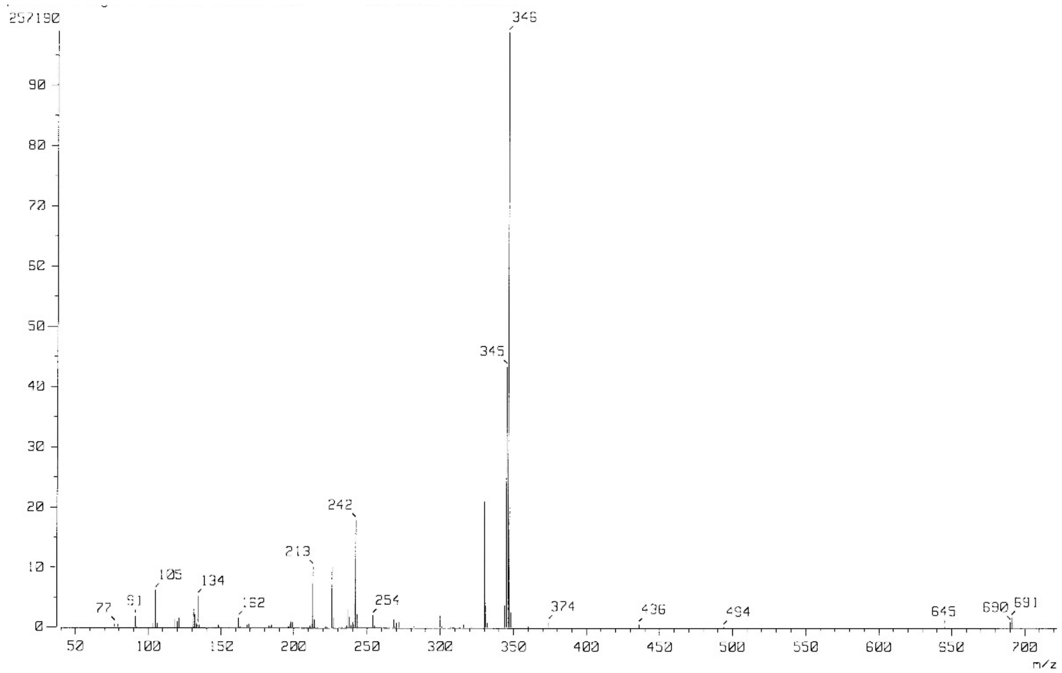

(2R,S)-2-Ethoxy-(5S)-5-methyl-4-[(1'S)-1-phenylethyl]-2,3,4,5-tetrahydro-1,4,2-benzoxazaphosphepine 2-oxide (**14b**). HRMS (CI$^+$): found for [M + H]$^+$, *m/z* 346.1553.

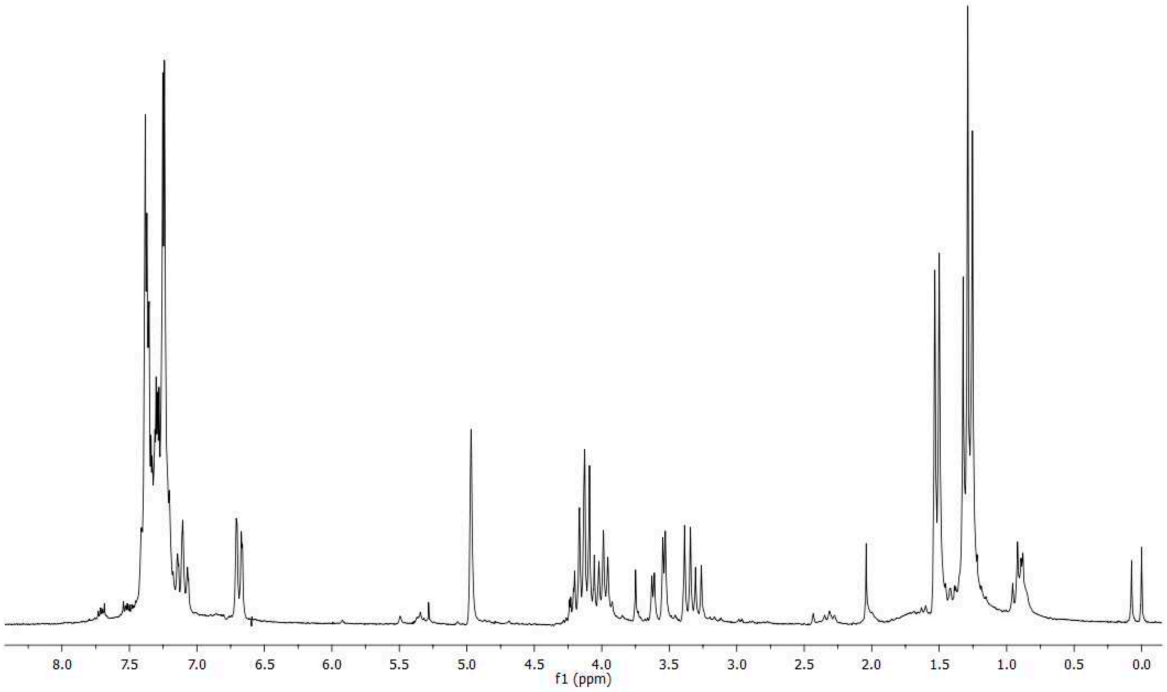

(2S)-2-Ethoxy-(5S)-5-phenyl-4-[(1'S)-1-phenylethyl]-2,3,4,5-tetrahydro-1,4,2-benzoxazaphosphepine 2-oxide (**13c**). ^{1}H-NMR ($CDCl_3$, 200 MHz): δ 1.28 (t, J = 7.0 Hz, 3H), 1.51 (d, J = 6.6 Hz, 3H), 3.32 (ABX system, $J_{H/P}$ = 16.2, 8.4 Hz, 1H), 3.57 (ABX system, $J_{H/P}$ = 16.2, 3.8 Hz, 1H), 3.99–4.23 (m, 3H), 4.96 (s, 1H), 6.67–7.71 (m, 14H).

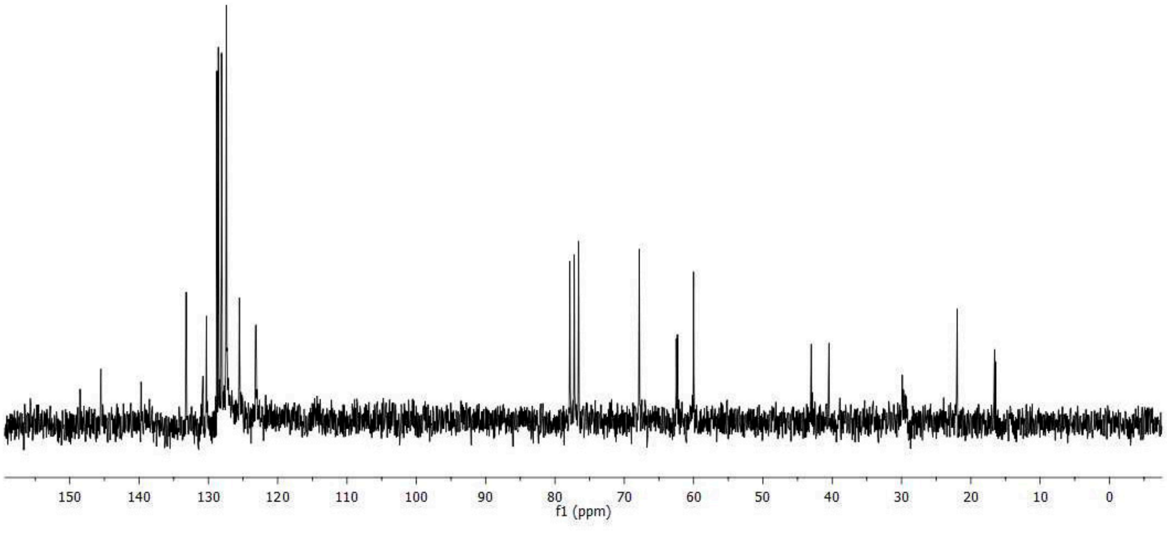

(2S)-2-Ethoxy-(5S)-5-phenyl-4-[(1'S)-1-phenylethyl]-2,3,4,5-tetrahydro-1,4,2-benzoxazaphosphepine 2-oxide (**13c**). ^{13}C-NMR ($CDCl_3$, 50 MHz): δ 16.5 (d, $J_{C/P}$ = 5.85 Hz), 21.9, 40.2 (d, $J_{C/P}$ = 128.15 Hz), 59.9, 62.4 (d, $J_{C/P}$ = 7.3 Hz), 67.8, 123.1, 123.2, 125.5, 127.4, 127.5, 128.1, 128.5, 128.8, 130.3, 130.8, 133.2, 139.7, 145.5.

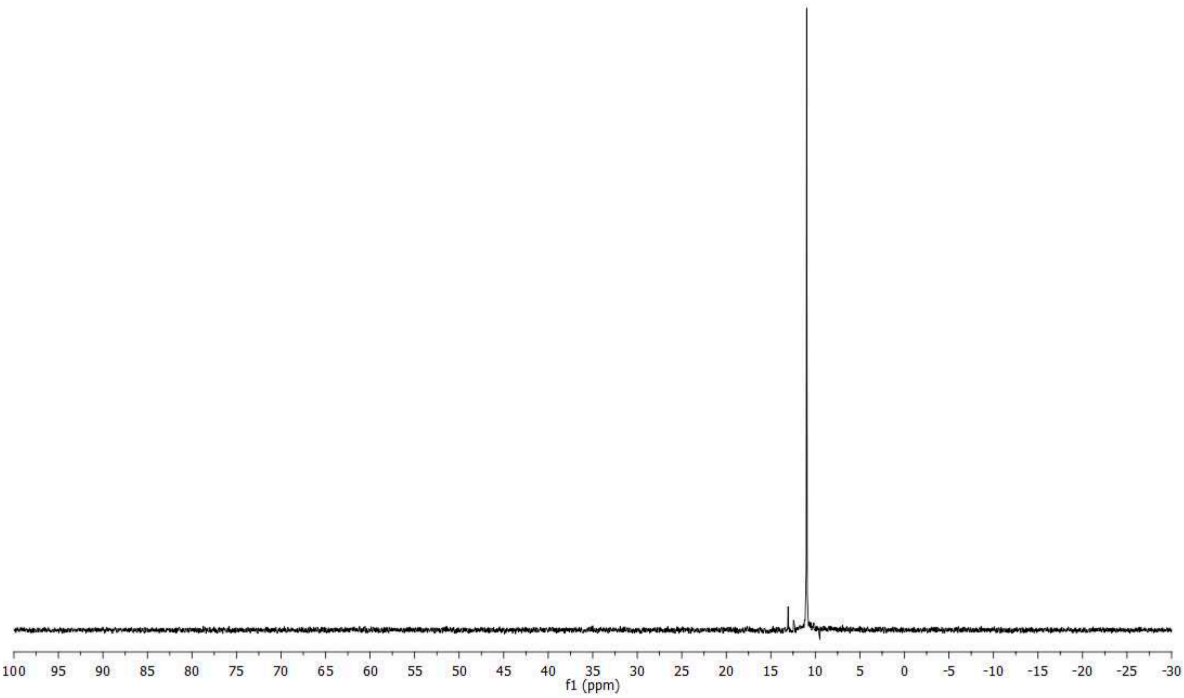

(2S)-2-Ethoxy-(5S)-5-phenyl-4-[(1'S)-1-phenylethyl]-2,3,4,5-tetrahydro-1,4,2-benzoxazaphosphepine 2-oxide (**13c**). ^{31}P-NMR ($CDCl_3$, 80.95 MHz): δ 10.97.

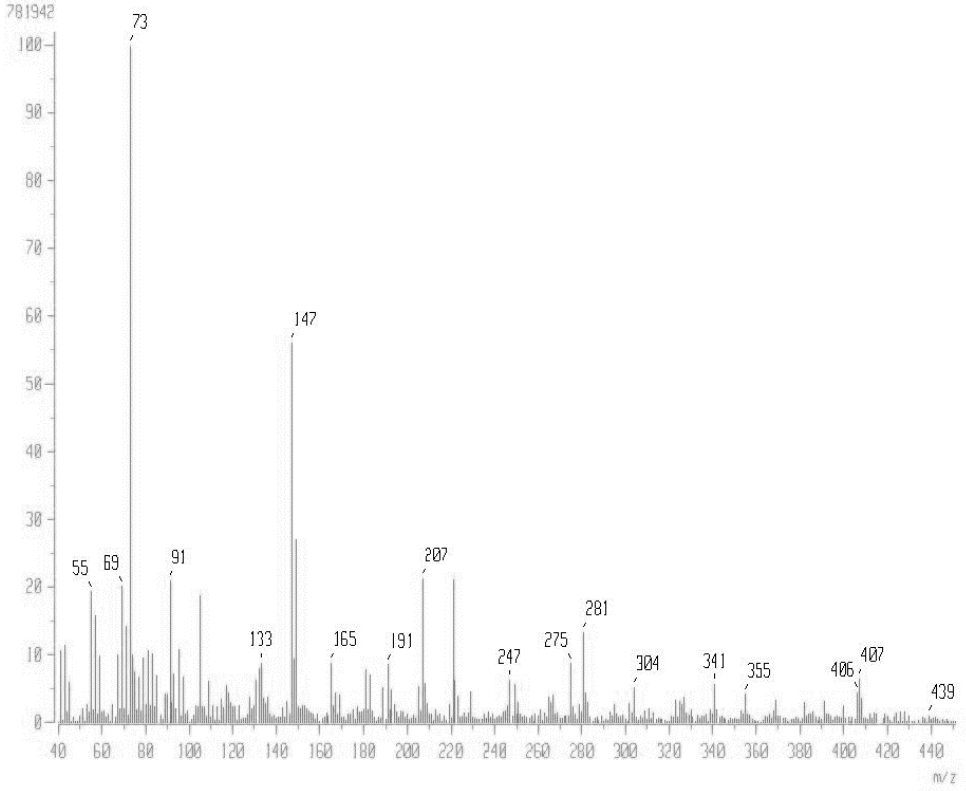

(2S)-2-Ethoxy-(5S)-5-phenyl-4-[(1'S)-1-phenylethyl]-2,3,4,5-tetrahydro-1,4,2-benzoxazaphosphepine 2-oxide (**13c**). HRMS (CI$^+$): found for $[M + H]^+$, *m*/*z* 408.1710.

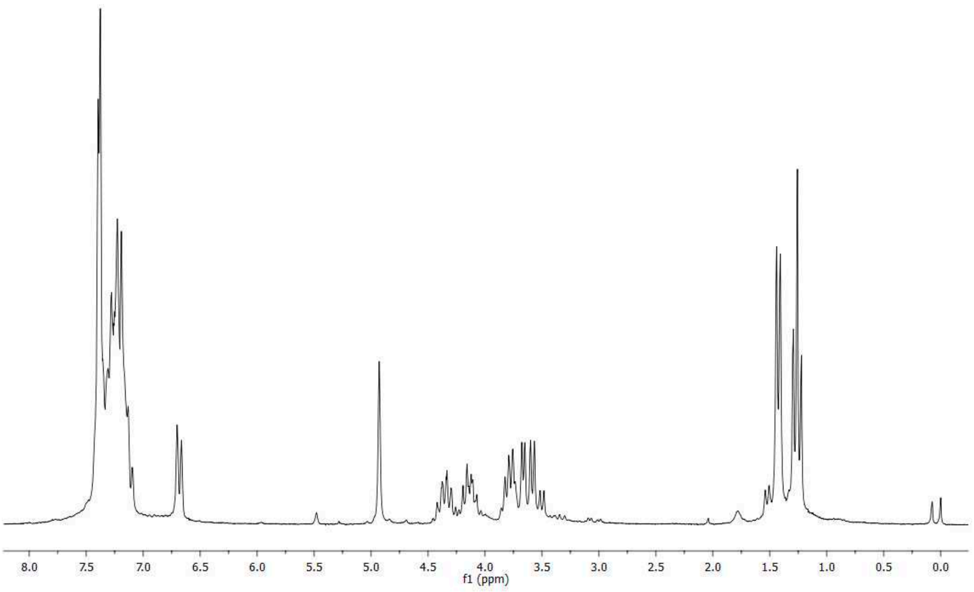

(2R)-2-Ethoxy-(5S)-5-phenyl-4-[(1'S)-1-phenylethyl]-2,3,4,5-tetrahydro-1,4,2-benzoxazaphosphepine 2-oxide (**14c**). ^{1}H-NMR ($CDCl_3$, 200 MHz): δ 1.25 (t, J = 7.0 Hz, 3H), 1.42 (d, J = 6.8 Hz, 3H), 3.54 (ABX system, $J_{H/P}$ = 16.8, 6.8 Hz, 1H), 3.70 (ABX system, $J_{H/P}$ = 16.0, 5.2 Hz, 1H), 3.80 (q, J = 6.8 Hz, 1H), 4.03–4.45 (m, 2H), 4.92 (s, 1H), 6.66–7.39 (m, 14H).

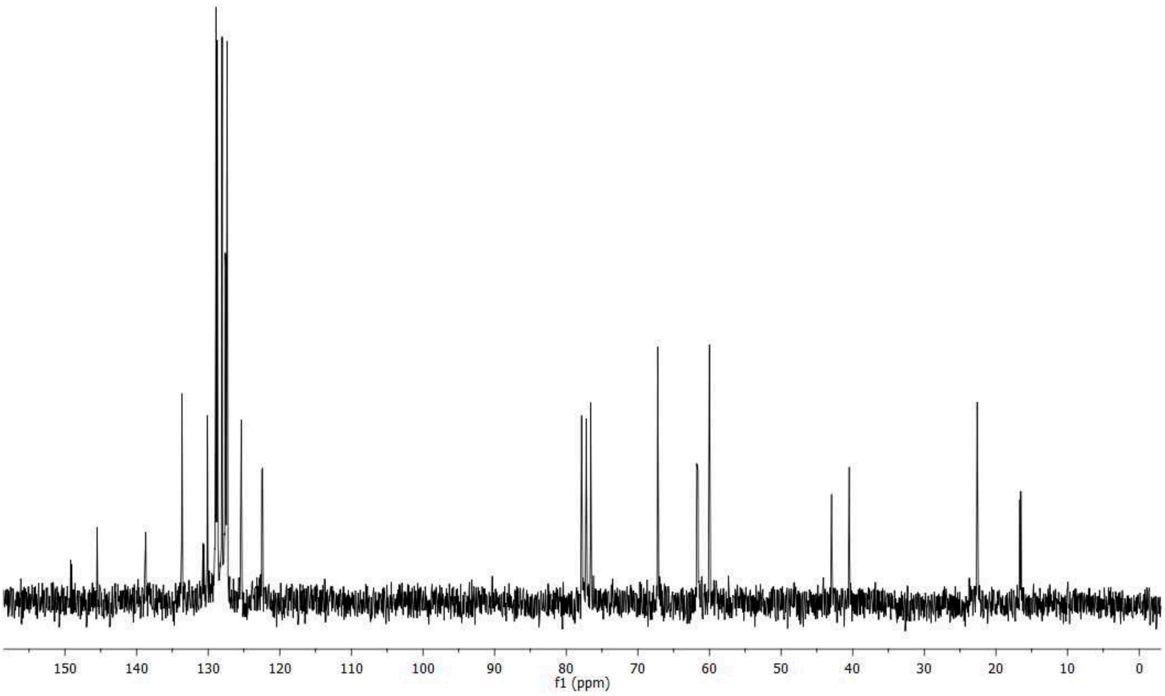

(2R)-2-Ethoxy-(5S)-5-phenyl-4-[(1'S)-1-phenylethyl]-2,3,4,5-tetrahydro-1,4,2-benzoxazaphosphepine 2-oxide (**14c**). ^{13}C-NMR ($CDCl_3$, 50 MHz): δ 16.5 (d, $J_{C/P}$ = 5.95 Hz), 22.6, 41.7 (d, $J_{C/P}$ = 123.3 Hz), 59.9, 61.6 (d, $J_{C/P}$ = 7.95 Hz), 67.2, 122.3, 122.4, 125.3, 127.3, 127.5, 127.6, 128.0, 128.7, 128.9, 130.0, 130.6, 130.7, 138.7, 145.4, 149.1 (d, $J_{C/P}$ = 7.2 Hz).

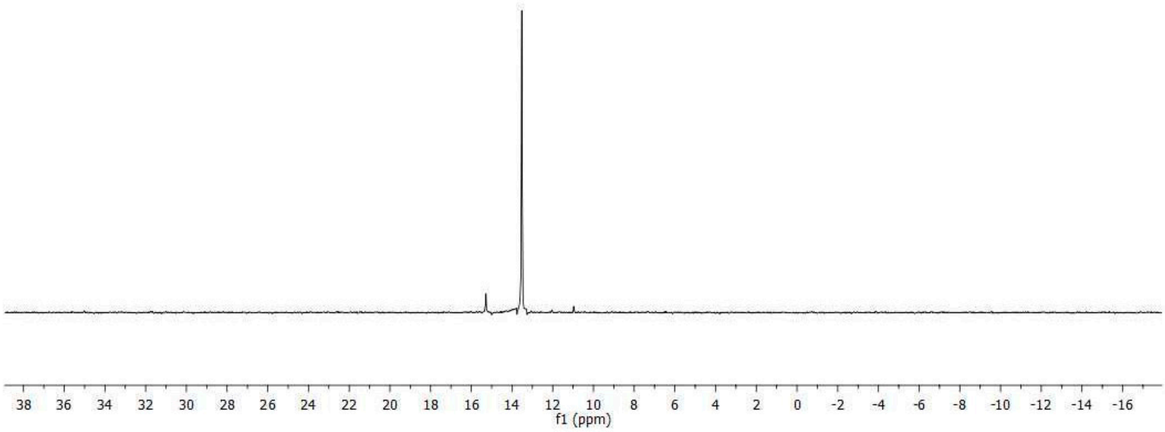

(2R)-2-Ethoxy-(5S)-5-phenyl-4-[(1'S)-1-phenylethyl]-2,3,4,5-tetrahydro-1,4,2-benzoxazaphosphepine 2-oxide (**14c**). ^{31}P-NMR ($CDCl_3$, 80.95 MHz): δ 13.52.

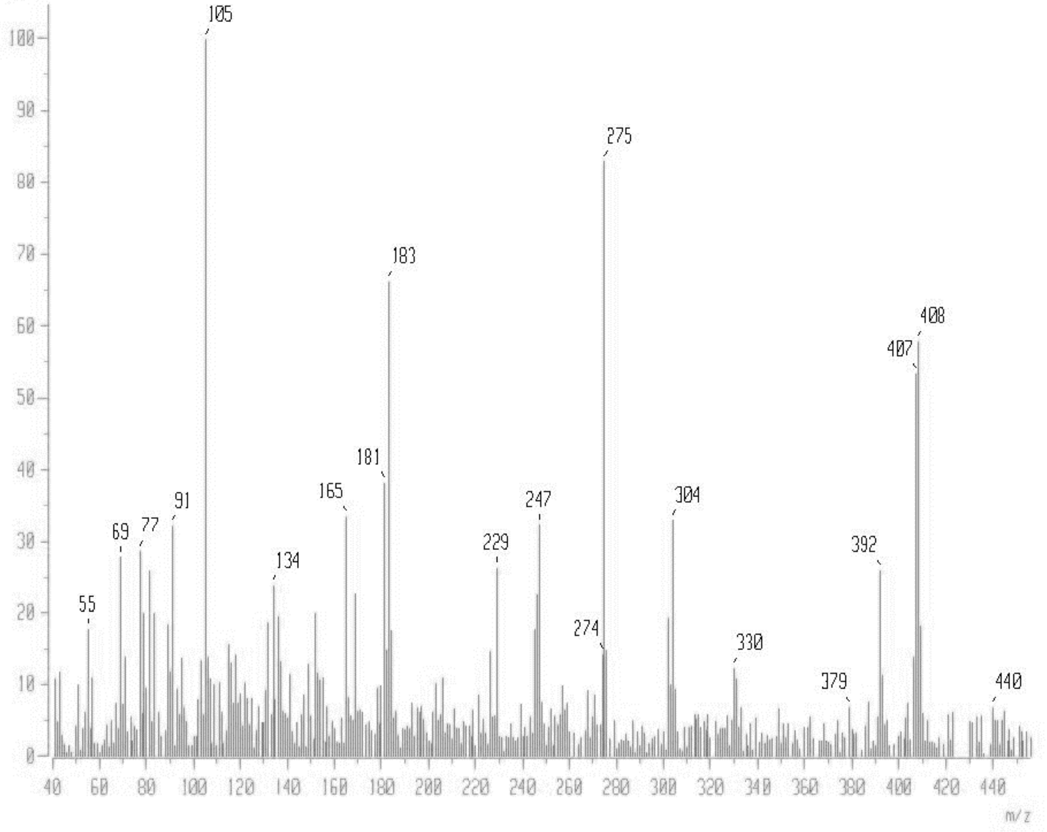

(2R)-2-Ethoxy-(5S)-5-phenyl-4-[(1'S)-1-phenylethyl]-2,3,4,5-tetrahydro-1,4,2-benzoxazaphosphepine 2-oxide (**14c**). HRMS (CI$^+$): found for $[M + H]^+$, *m/z* 408.1710.

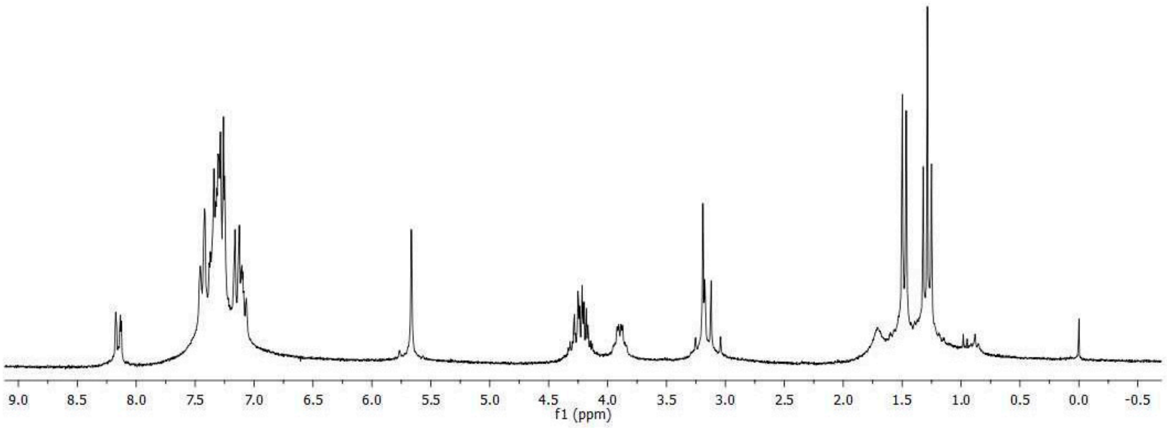

(2R,S)-2-Ethoxy-(5R)-5-(2-chlrophenyl)-4-[(1'S)-1-phenylethyl]-2,3,4,5-tetrahydro-1,4,2-benzoxazaphosphepine 2-oxide (**13d**). ^{1}H-NMR ($CDCl_3$, 400 MHz): δ 1.28 (t, J = 7.0 Hz, 3H), 1.48 (d, J = 6.8 Hz, 3H), 3.11 (AB system, J = 16.4 Hz, 1H), 3.20 (AB system, J = 15.6 Hz, 1H), 3.89 (dq, J = 6.6, 3.2 Hz, 1H), 4.22 (m, 2H), 5.66 (s, 1H), 7.07–7.44 (m, 13H).

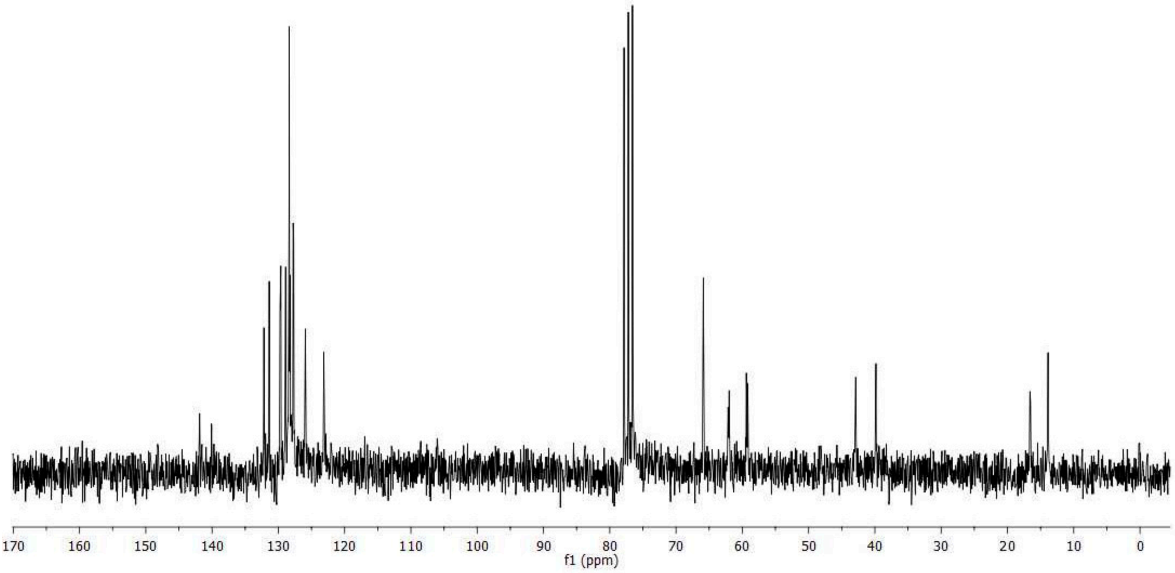

(2R,S)-2-Ethoxy-(5R)-5-(2-chlrophenyl)-4-[(1'S)-1-phenylethyl]-2,3,4,5-tetrahydro-1,4,2-benzoxazaphosphepine 2-oxide (**13d**). ^{13}C-NMR ($CDCl_3$, 100 MHz): δ 13.8, 16.5, 16.6, 41.3 (d, $J_{C/P}$ = 153.75 Hz), 59.3 (d, $J_{C/P}$ = 11.7 Hz), 62.0, 65.8, 105.2, 123.0, 123.1, 125.9, 127.7, 127.8, 128.2, 128.3, 128.9, 129.6, 129.7, 131.3, 131.8, 132.1, 140.0, 141.8.

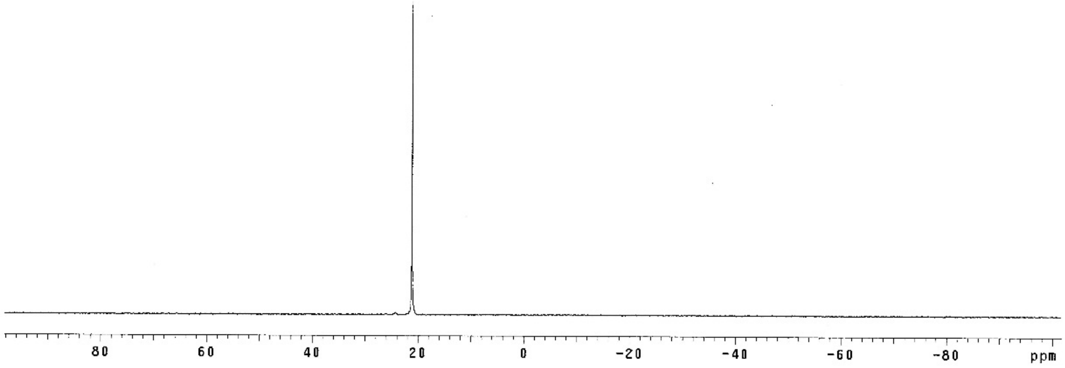

(2R,S)-2-Ethoxy-(5R)-5-(2-chlrophenyl)-4-[(1'S)-1-phenylethyl]-2,3,4,5-tetrahydro-1,4,2-benzoxazaphosphepine 2-oxide (**13d**). ^{31}P-NMR (CDCl$_3$, 80.95 MHz): δ 21.21.

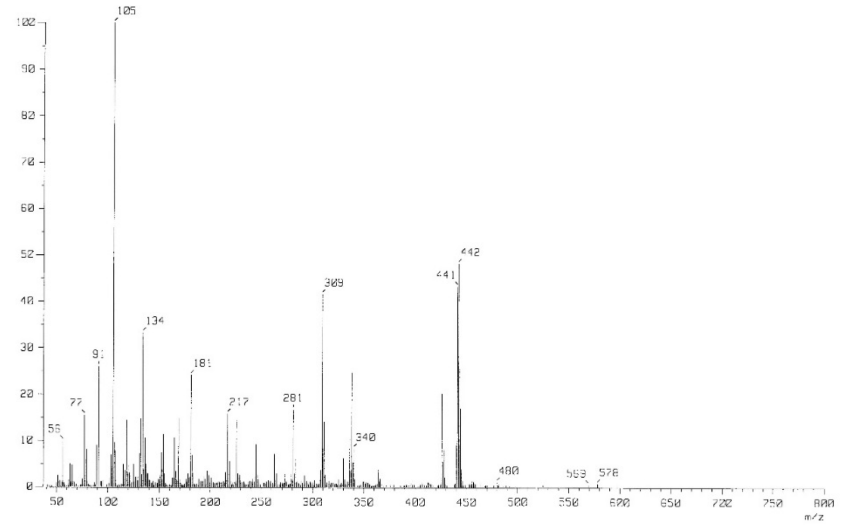

(2R,S)-2-Ethoxy-(5R)-5-(2-chlrophenyl)-4-[(1'S)-1-phenylethyl]-2,3,4,5-tetrahydro-1,4,2-benzoxazaphosphepine 2-oxide (**13d**). HRMS (CI$^+$): found for [M + H]$^+$, *m/z* 442.1361.

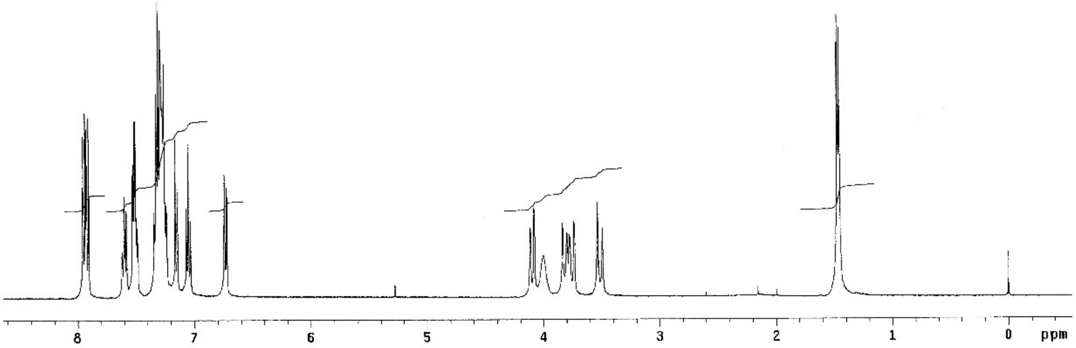

(2S)-2-Phenyl-4-[(1'S)-1-phenylethyl]-2,3,4,5-tetrahydro-1,4,2-benzoxazaphosphepine 2-oxide (**16a**). ^{1}H-NMR (CDCl$_3$, 400 MHz): δ 1.46 (d, *J* = 6.4 Hz, 3H), 3.51 (AB system, *J* = 15.2 Hz, 1H), 3.75 (ABX system, *J* = 15.2, 3.2 Hz, 1H), 3.81 (AB system, *J* = 14.4 Hz, 1H), 3.99 (dq, *J* = 7.0, 4.0 Hz, 1H), 4.09 (ABX system, *J* = 14.8, 1.6 Hz, 1H), 6.75–8.02 (m, 14H).

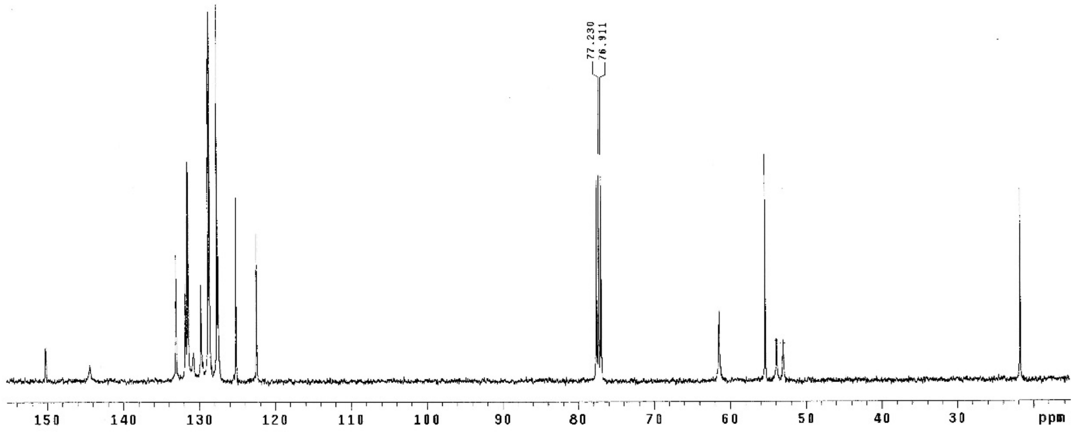

(2S)-2-Phenyl-4-[(1'S)-1-phenylethyl]-2,3,4,5-tetrahydro-1,4,2-benzoxazaphosphepine 2-oxide (**16a**). ^{13}C-NMR ($CDCl_3$, 100 MHz): δ 21.7, 53.4 (d, $J_{C/P}$ = 89.5 Hz), 55.3, 61.3, 122.4 (d, $J_{C/P}$ = 3.0 Hz), 125.1, 127.4, 127.6, 128.6, 128.7, 128.8, 129.7, 130.7, 131.4, 131.5, 131.7, 133.0, 144.3, 150.1 (d, $J_{C/P}$ = 6.1 Hz).

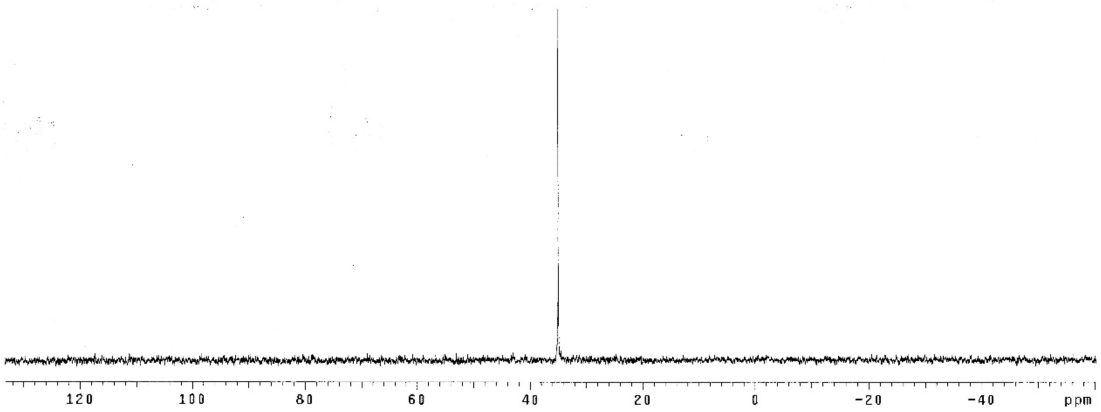

(2S)-2-Phenyl-4-[(1'S)-1-phenylethyl]-2,3,4,5-tetrahydro-1,4,2-benzoxazaphosphepine 2-oxide (**16a**). ^{31}P-NMR ($CDCl_3$, 80.95 MHz): δ 34.87.

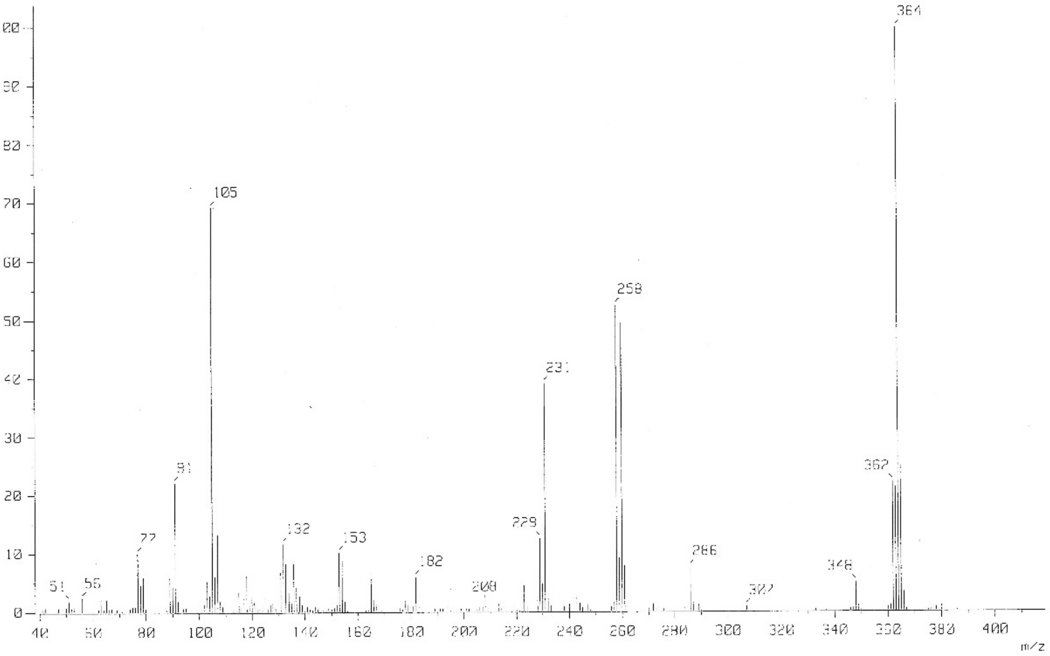

(2S)-2-Phenyl-4-[(1'S)-1-phenylethyl]-2,3,4,5-tetrahydro-1,4,2-benzoxazaphosphepine 2-oxide (**16a**). HRMS (CI^+): found for $[M + H]^+$, *m/z* 364.1454.

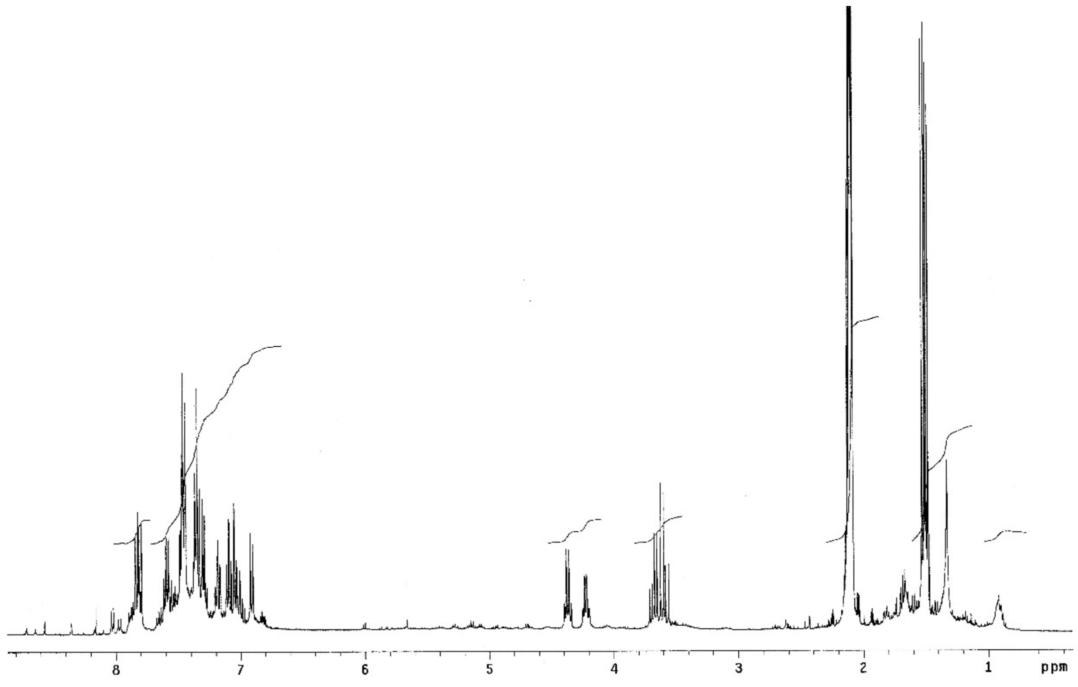

(2R)-2-Phenyl-(5S)-5-methyl-4-[(1'S)-1-phenylethyl]-2,3,4,5-tetrahydro-1,4,2-benzoxazaphosphepine 2-oxide (**15b**). ^{1}H-NMR ($CDCl_3$, 200 MHz): δ 1.48 (d, J = 6.8 Hz, 3H), 1.51 (d, J = 7.6 Hz, 3H), 3.58 (ABX system, J = 15.4, 10.6 Hz, 1H), 3.67 (ABX system, J = 15.8, 7.8 Hz, 1H), 4.22 (q, J = 6.8 Hz, 1H), 4.36 (q, J = 7.2 Hz, 1H), 6.90–7.84 (m, 14H).

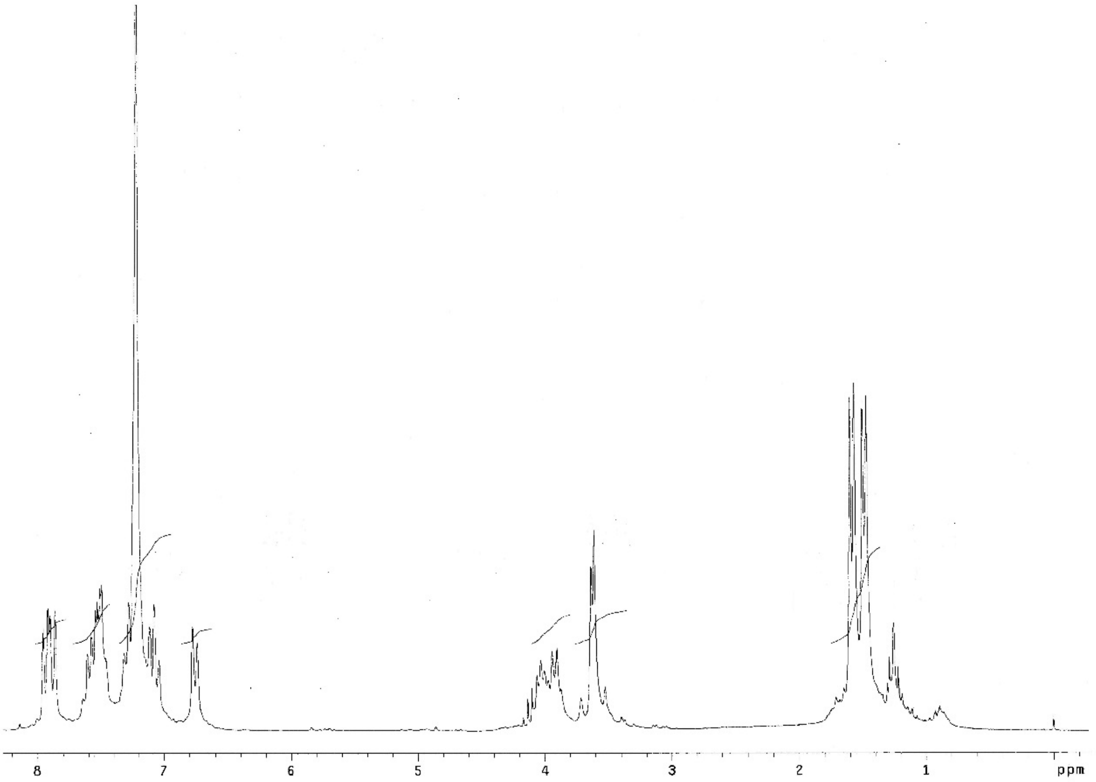

(2S)-2-Phenyl-(5S)-5-methyl-4-[(1'S)-1-phenylethyl]-2,3,4,5-tetrahydro-1,4,2-benzoxazaphosphepine 2-oxide (**16b**). ^{1}H-NMR ($CDCl_3$, 200 MHz): δ 1.48 (d, J = 6.6 Hz, 3H), 1.58 (d, J = 7.0 Hz, 3H), 3.56 (AB system, J = 16 Hz, 1H), 3.67 (AB system, J = 16.4 Hz, 1H), 3.91 (q, J = 6.9 Hz, 1H), 4.04 (q, J = 6.5 Hz, 1H), 6.8–7.94 (m, 14H).

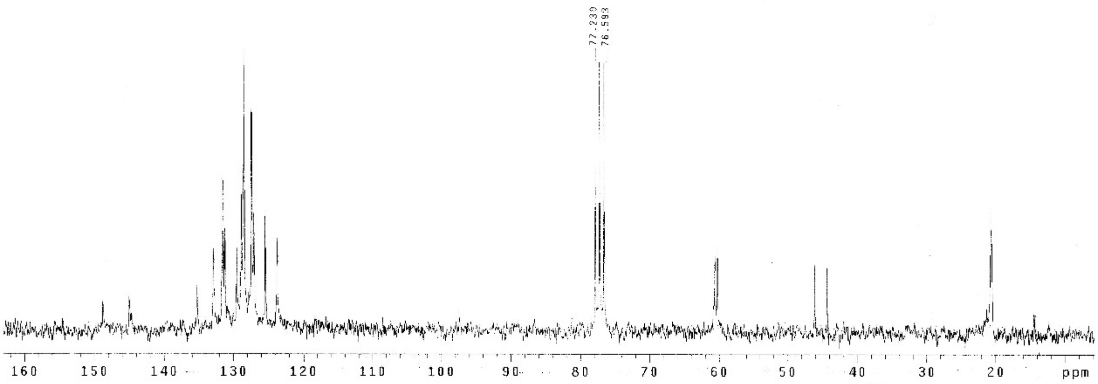

(2S)-2-Phenyl-(5S)-5-methyl-4-[(1'S)-1-phenylethyl]-2,3,4,5-tetrahydro-1,4,2-benzoxazaphosphepine 2-oxide (**16b**). ^{13}C-NMR ($CDCl_3$, 50 MHz): δ 20.5, 20.6, 45.1 (d, $J_{C/P}$ = 87.9 Hz), 60.2, 60.6, 123.6, 125.3, 127.0, 127.3, 128.4, 128.5, 128.8, 129.4, 131.1, 131.5 (d, $J_{C/P}$ = 9.1 Hz), 132.8, 135.2, 144.9, 148.7 (d, $J_{C/P}$ = 7.6 Hz).

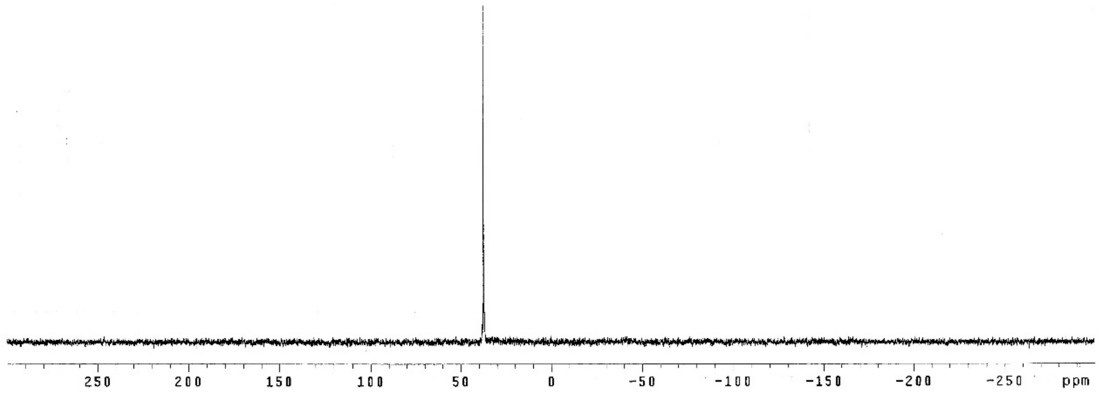

(2S)-2-Phenyl-(5S)-5-methyl-4-[(1'S)-1-phenylethyl]-2,3,4,5-tetrahydro-1,4,2-benzoxazaphosphepine 2-oxide (**16b**). ^{31}P-NMR ($CDCl_3$, 161.8 MHz): δ 37.23.

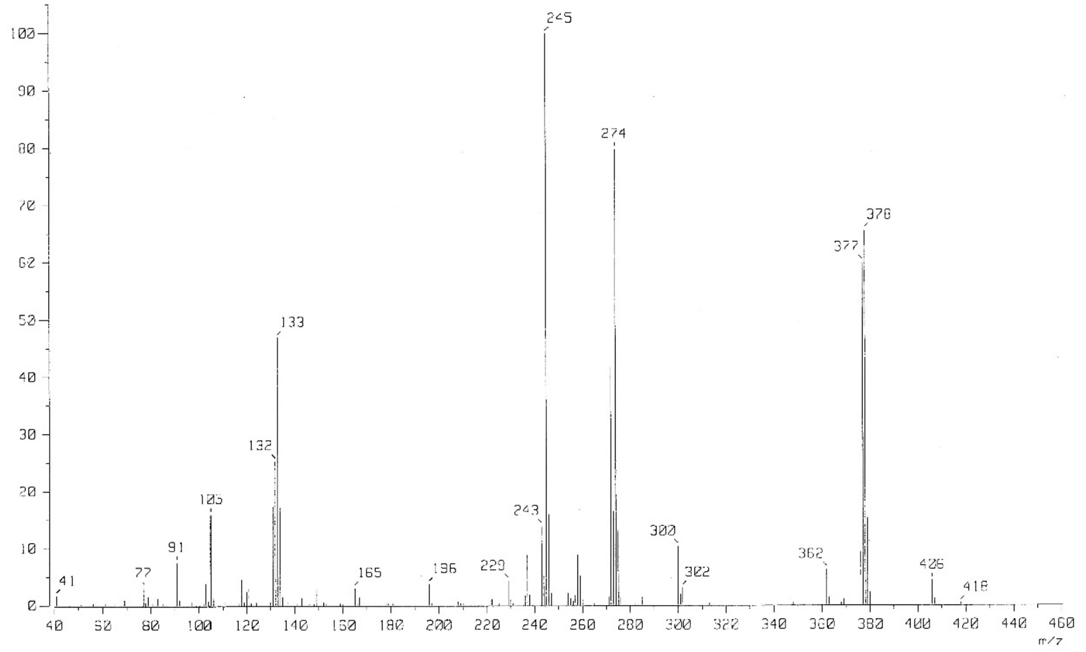

(2S)-2-Phenyl-(5S)-5-methyl-4-[(1'S)-1-phenylethyl]-2,3,4,5-tetrahydro-1,4,2-benzoxazaphosphepine 2-oxide (**16b**). HRMS (CI^+): found for $[M + H]^+$, *m/z* 378.1612.

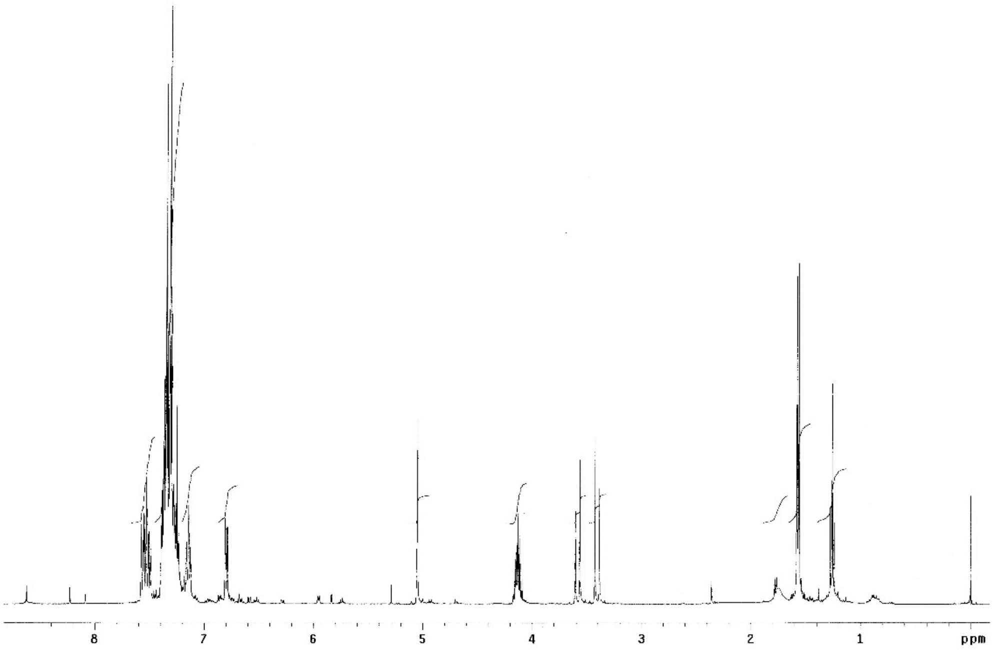

(2R,S)-2-phenyl-(5S)-5-phenyl-4-[(1'S)-1-phenylethyl]-2,3,4,5-tetrahydro-1,4,2-benzoxazaphosphepine 2-oxide (**15c**). ^{1}H-NMR ($CDCl_3$, 200 MHz): δ 1.29 (d, J = 6.8 Hz, 3H), 3.35 (ABX system, J = 16.4, 14.6 Hz, 1H), 3.49 (ABX system, J = 16.4, 6.4 Hz, 1H), 4.09 (dq, J = 6.8, 6.4 Hz, 1H), 5.29 (s, 1H), 6.70–7.59 (m, 19H).

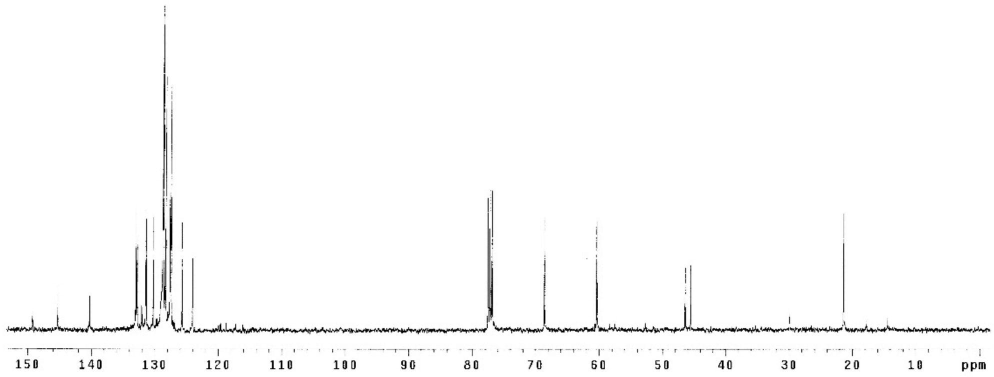

(2R,S)-2-phenyl-(5S)-5-phenyl-4-[(1'S)-1-phenylethyl]-2,3,4,5-tetrahydro-1,4,2-benzoxazaphosphepine 2-oxide (**15c**). ^{13}C-NMR ($CDCl_3$, 50 MHz): δ 15.9, 29.8, 45.3 (d, $J_{C/P}$ = 95.2), 60.0 (d, J = 7.3 Hz), 69.1, 123.6, 123.7, 125.8, 127.6, 127.7, 127.9, 128.5, 128.6, 128.7, 128.8, 129.4, 131.4, 131.5, 132.0, 132.8, 132.9, 142.4 (d, $J_{C/P}$ = 99.5 Hz).

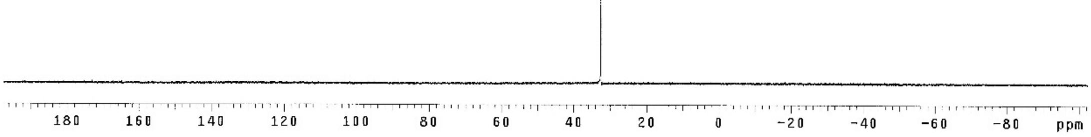

(2R,S)-2-phenyl-(5S)-5-phenyl-4-[(1'S)-1-phenylethyl]-2,3,4,5-tetrahydro-1,4,2-benzoxazaphosphepine 2-oxide (**15c**). ^{31}P-NMR ($CDCl_3$, 161.8 MHz): δ 36.74.

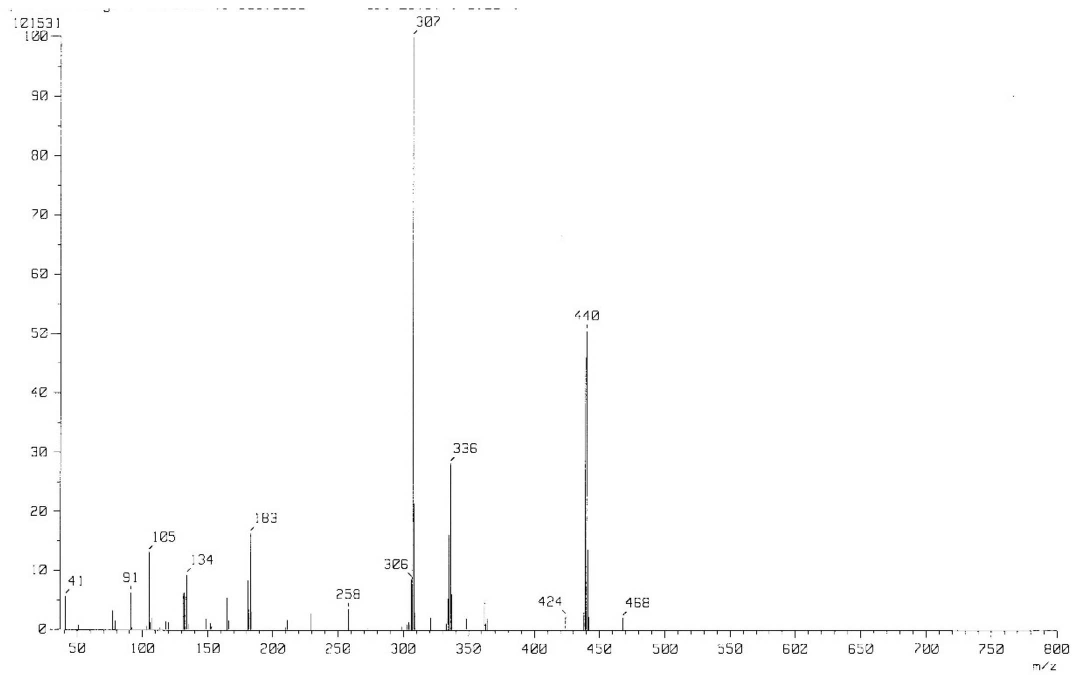

(2R,S)-2-phenyl-(5S)-5-phenyl-4-[(1'S)-1-phenylethyl]-2,3,4,5-tetrahydro-1,4,2-benzoxazaphosphepine 2-oxide (**15c**). HRMS (CI^+): found for $[M + H]^+$, *m*/*z* 440.1774.

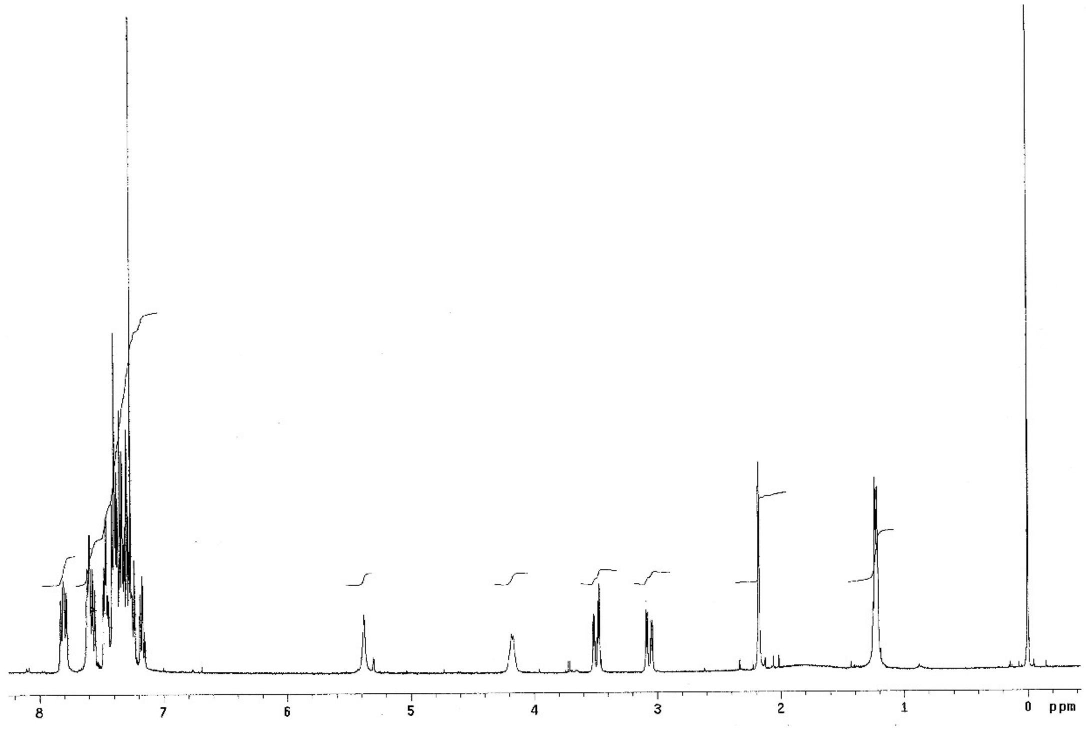

(2R,S)-2-Phenyl-(5S)-5-phenyl-4-[(1'S)-1-phenylethyl]-2,3,4,5-tetrahydro-1,4,2-benzoxazaphosphepine 2-oxide (**16c**). ^{1}H-NMR ($CDCl_3$, 200 MHz): δ 1.56 (d, *J* = 6.4 Hz, 3H), 3.40 (AB system, *J* = 16.4 Hz, 1H), 3.58 (AB system, *J* = 16.4 Hz, 1H), 4.14 (q, *J* = 6.0 Hz, 1H), 5.05 (s, 1H), 7.12–7.57 (m, 19H).

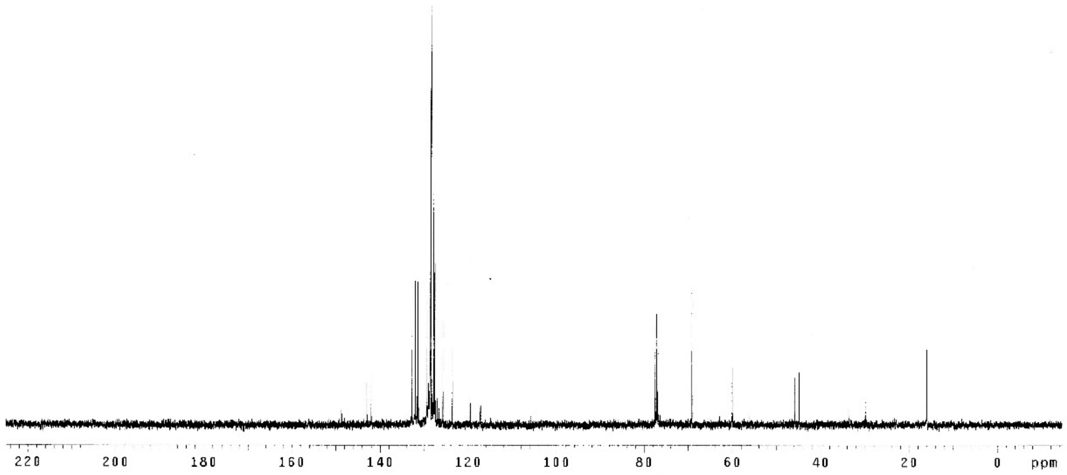

(2R,S)-2-Phenyl-(5S)-5-phenyl-4-[(1'S)-1-phenylethyl]-2,3,4,5-tetrahydro-1,4,2-benzoxazaphosphepine 2-oxide (**16c**). ^{13}C-NMR ($CDCl_3$, 50 MHz): δ 21.3, 20.6, 45.9 (d, $J_{C/P}$ = 84.9 Hz), 60.3, 68.5, 123.9, 125.6, 127.4, 127.5, 127.6, 128.2, 128.6, 128.7, 130.2, 131.3, 131.4, 132.7, 132.9, 149.22.

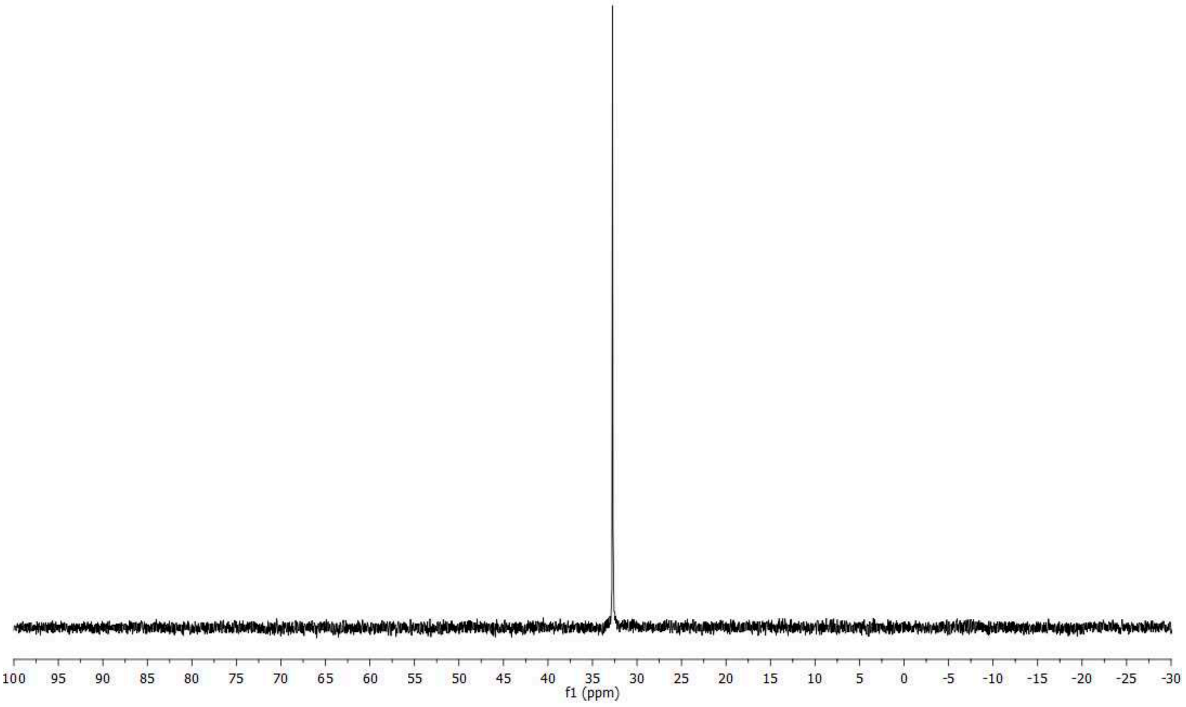

(2R,S)-2-Phenyl-(5S)-5-phenyl-4-[(1'S)-1-phenylethyl]-2,3,4,5-tetrahydro-1,4,2-benzoxazaphosphepine 2-oxide (**16c**). ^{31}P-NMR ($CDCl_3$, 161.8 MHz): δ 32.57.

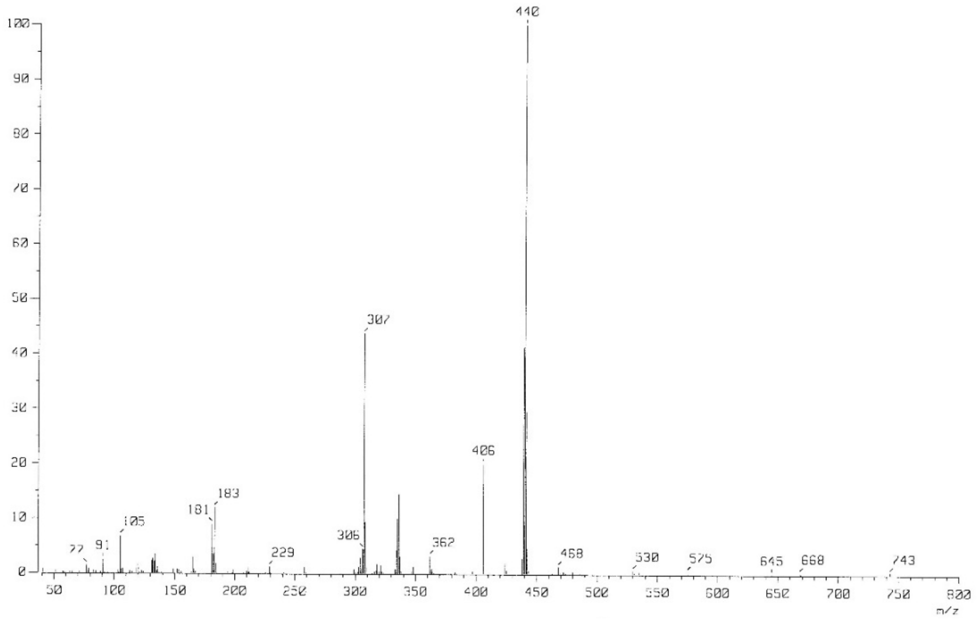

(2R,S)-2-Phenyl-(5S)-5-phenyl-4-[(1'S)-1-phenylethyl]-2,3,4,5-tetrahydro-1,4,2-benzoxazaphosphepine 2-oxide (**16c**). HRMS (CI^+): found for $[M + H]^+$, *m*/*z* 440.1783.

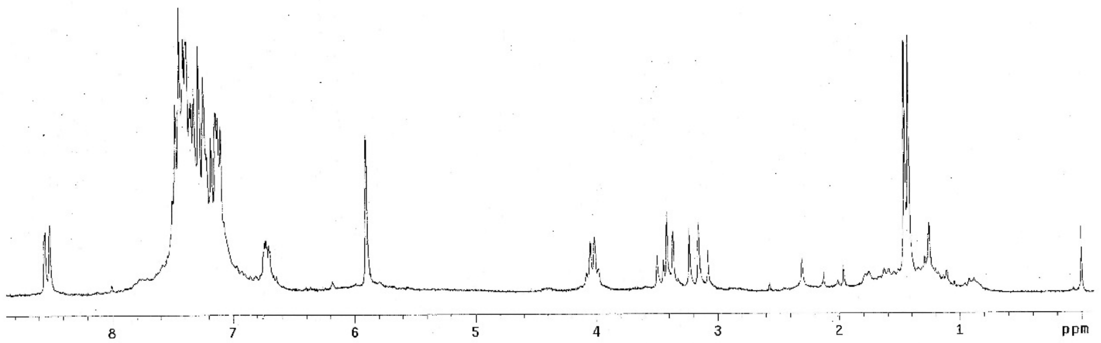

(2R)-2-Phenyl-(5R)-5-(2-chlorophenyl)-4-[(1'S)-1-phenylethyl]-2,3,4,5-tetrahydro-1,4,2-benzoxazaphosphepine 2-oxide (**15d**). ^{1}H-NMR ($CDCl_3$, 200 MHz): δ 1.44 (d, *J* = 7.0 Hz, 3H), 3.15 (ABX system, *J* = 15.2, 15.2 Hz, 1H), 3.43 (ABX system, *J* = 16.1, 9.9 Hz, 1H), 4.02 (q, *J* = 6.6 Hz, 1H), 5.90 (s, 1H), 6.70–8.54 (m, 18H).

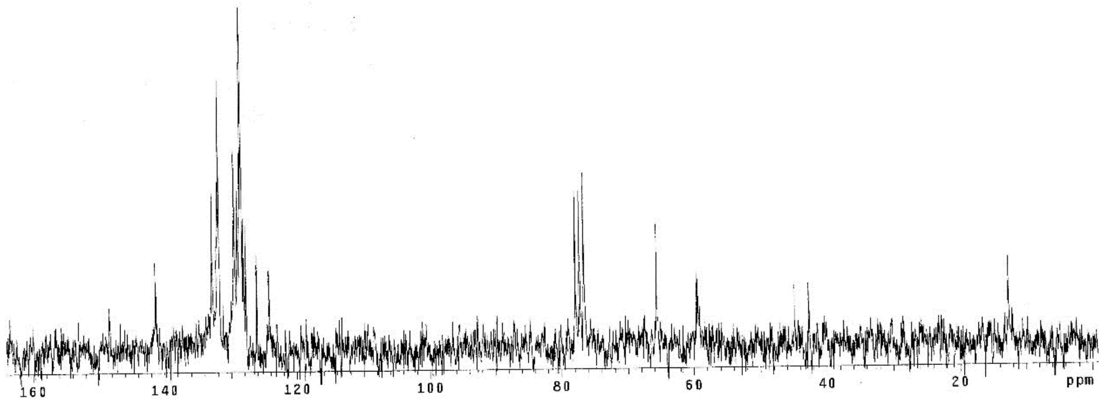

(2R)-2-Phenyl-(5R)-5-(2-chlorophenyl)-4-[(1'S)-1-phenylethyl]-2,3,4,5-tetrahydro-1,4,2-benzoxazaphosphepine 2-oxide (**15d**). ^{13}C-NMR ($CDCl_3$, 50 MHz): δ 12.6, 43.7 (d, $J_{C/P}$ = 104.9 Hz), 59.5, 65.5, 124.2, 126.0, 127.7, 128.0, 128.3, 128.5, 128.9, 129.3, 131.7, 131.9, 132.7, 141.2, 148.3.

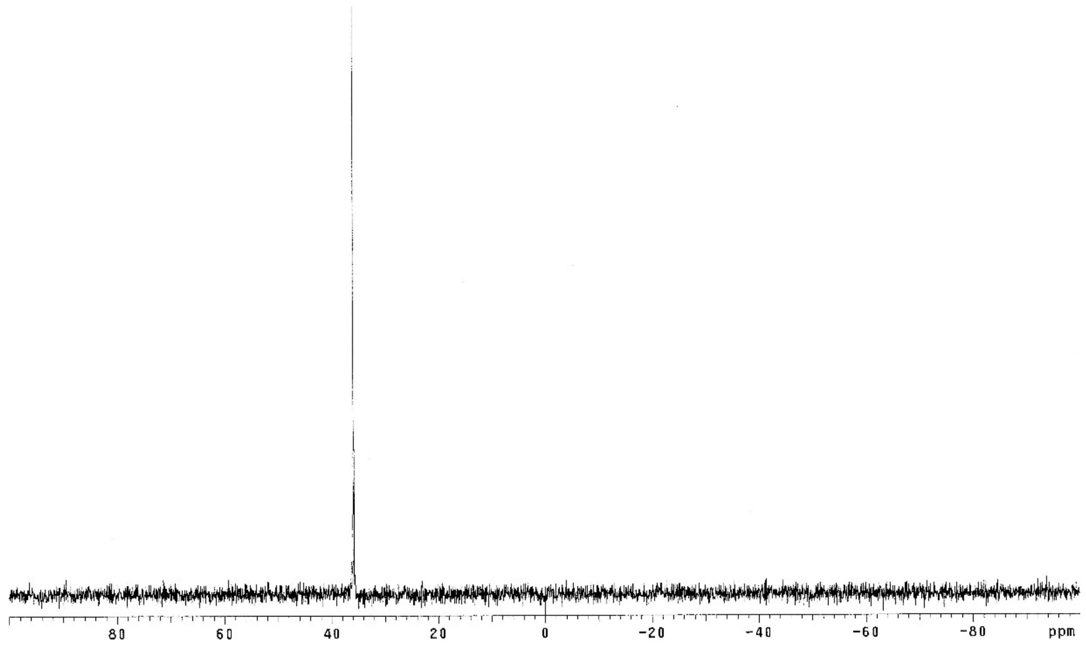

(2R)-2-Phenyl-(5R)-5-(2-chlorophenyl)-4-[(1'S)-1-phenylethyl]-2,3,4,5-tetrahydro-1,4,2-benzoxazaphosphepine 2-oxide (**15d**). ^{31}P-NMR ($CDCl_3$, 81 MHz): δ 38.60.

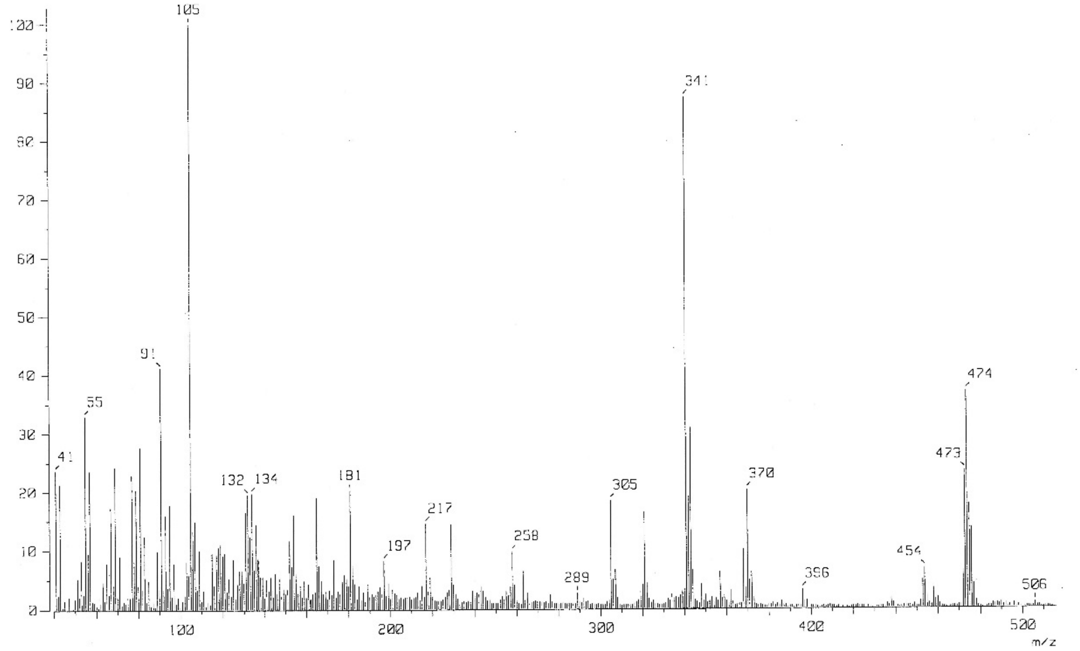

(2R)-2-Phenyl-(5R)-5-(2-chlorophenyl)-4-[(1'S)-1-phenylethyl]-2,3,4,5-tetrahydro-1,4,2-benzoxazaphosphepine 2-oxide (**15d**). HRMS (CI^+): found for $[M + H]^+$, *m*/*z* 474.1437.

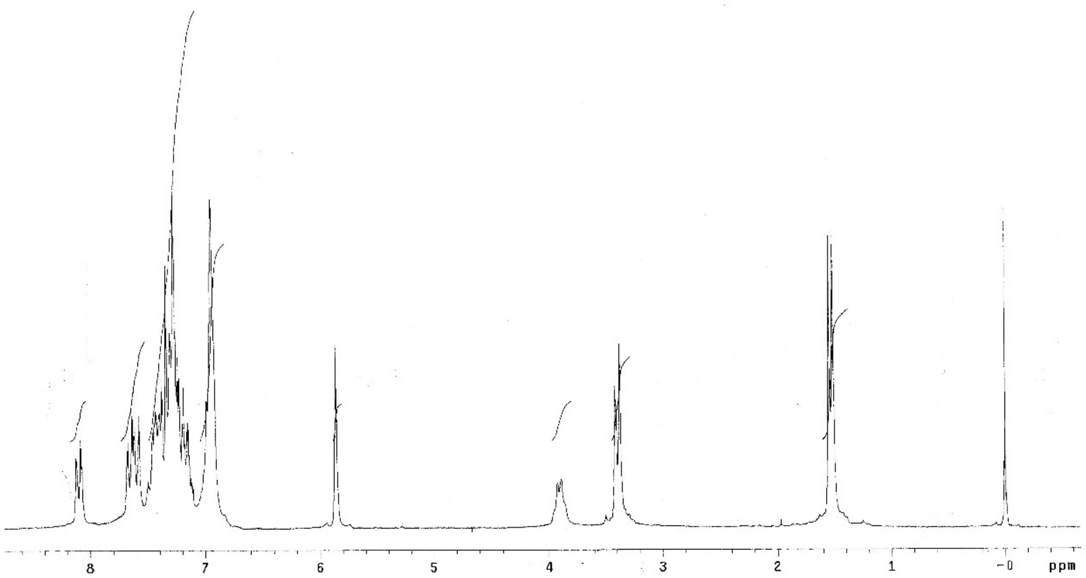

(2S)-2-Phenyl-(5R)-5-(2-chlorophenyl)-4-[(1'S)-1-phenylethyl]-2,3,4,5-tetrahydro-1,4,2-benzoxazaphosphepine 2-oxide (**16d**). ^{1}H-NMR ($CDCl_3$, 400 MHz): δ 1.54 (d, J = 6.8 Hz, 3H), 3.37 (ABX system, J = 16.0, 6.4 Hz, 1H), 3.42 (ABX system, J = 16.0, 8.4 Hz, 1H), 3.93 (bs, 1H), 5.85 (s, 1H), 6.94–8.11 (m, 18H).

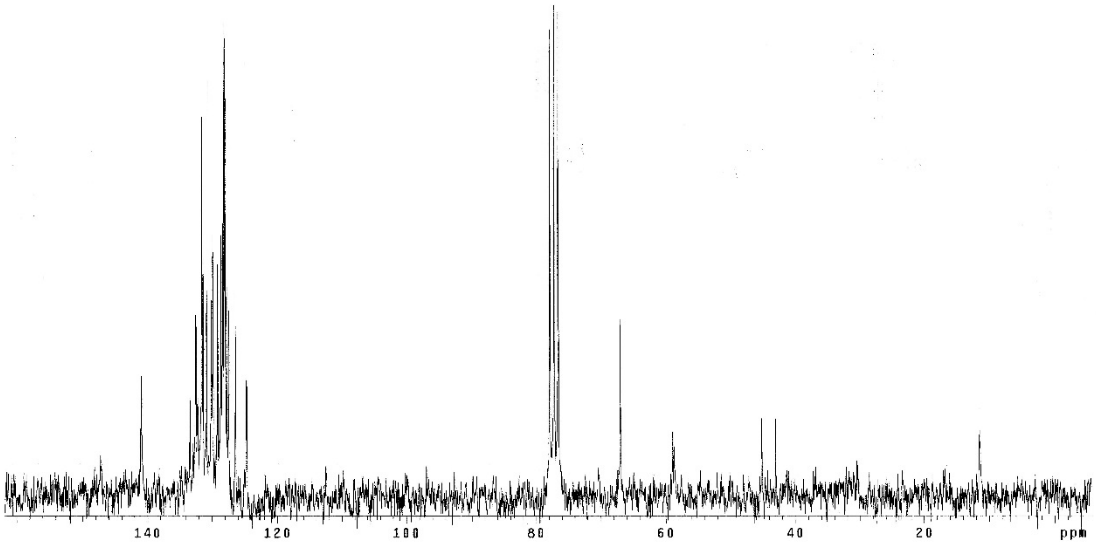

(2S)-2-Phenyl-(5R)-5-(2-chlorophenyl)-4-[(1'S)-1-phenylethyl]-2,3,4,5-tetrahydro-1,4,2-benzoxazaphosphepine 2-oxide (**16d**). ^{13}C-NMR ($CDCl_3$, 50 MHz): δ 11.4, 44.0 (d, $J_{C/P}$ = 107.9 Hz), 58.8, 66.9, 124.7, 126.3, 127.3, 127.7, 127.9, 128.1, 128.3, 128.5, 129.0, 129.8, 130.0, 130.7, 131.2, 131.4, 132.1, 132.4, 133.3, 140.9, 147.2.

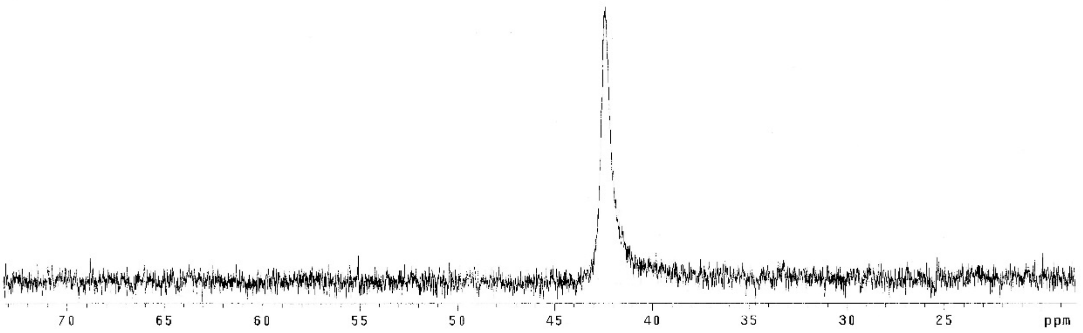

(2S)-2-Phenyl-(5R)-5-(2-chlorophenyl)-4-[(1'S)-1-phenylethyl]-2,3,4,5-tetrahydro-1,4,2-benzoxazaphosphepine 2-oxide (**16d**). ^{31}P-NMR ($CDCl_3$, 81 MHz): δ 42.36.

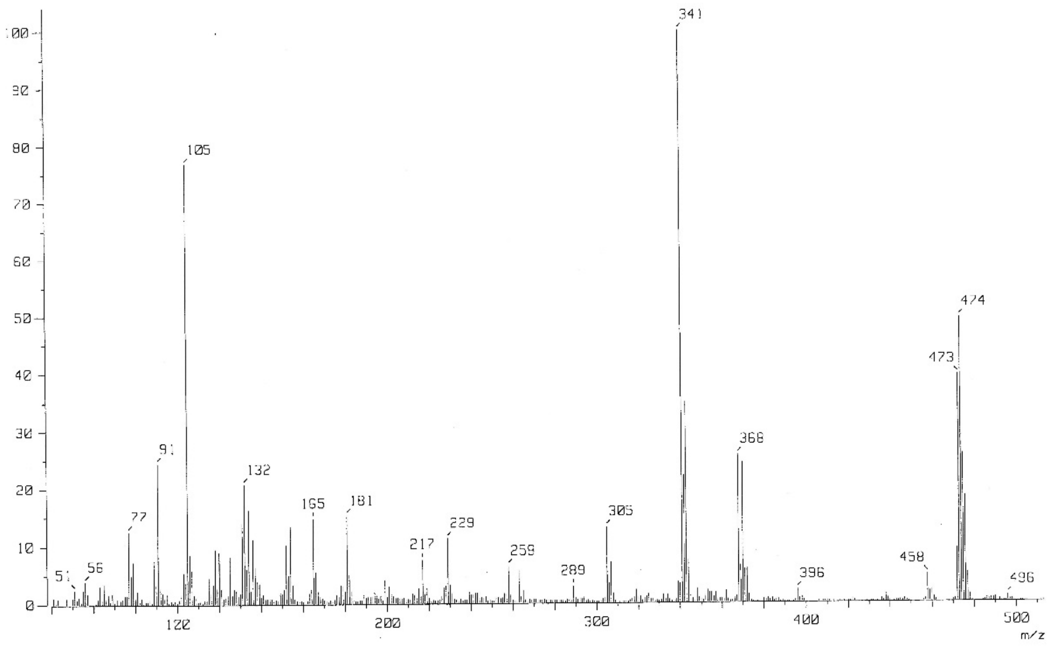

(2S)-2-Phenyl-(5R)-5-(2-chlorophenyl)-4-[(1'S)-1-phenylethyl]-2,3,4,5-tetrahydro-1,4,2-benzoxazaphosphepine 2-oxide (**16d**). HRMS (CI^+): found for $[M + H]^+$, *m*/*z* 474.1390.

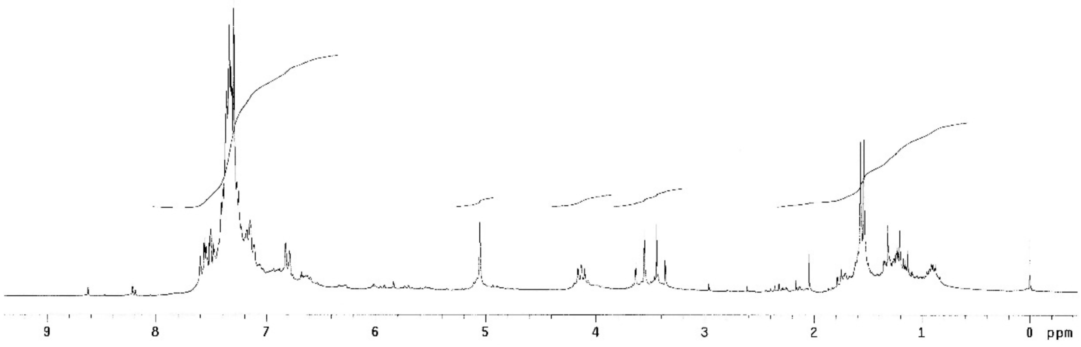

(2S)-2-Phenyl-(5R)-5-phenyl-4-[(1'S)-1-phenylethyl]-2,3,4,5-tetrahydro-1,4,2-benzoxazaphosphepine 2-oxide (**17c**). ^{1}H-NMR ($CDCl_3$, 200 MHz): δ 1.30 (d, *J* = 6.6 Hz, 3H), 3.36 (ABX system, *J* = 16.0, 12.2 Hz, 1H), 3.52 (ABX system, *J* = 16.1, 5.1 Hz, 1H), 4.11 (dq, *J* = 7.0, 2.2 Hz, 1H), 5.30 (s, 1H), 6.91–7.63 (m, 19H).

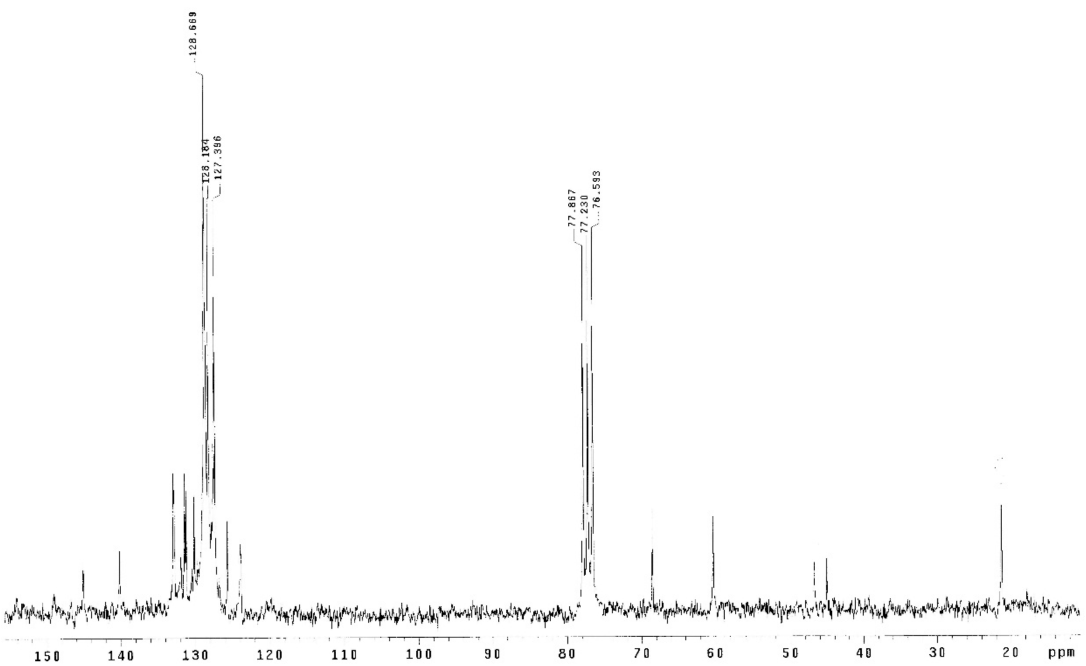

(2S)-2-Phenyl-(5R)-5-phenyl-4-[(1'S)-1-phenylethyl]-2,3,4,5-tetrahydro-1,4,2-benzoxazaphosphepine 2-oxide (**17c**). ^{13}C-NMR ($CDCl_3$, 50 MHz): δ 15.9, 45.3 (d, $J_{C/P}$ = 95.5 Hz), 59.9, 69.1, 123.5, 125.7, 127.6, 127.8, 128.4, 128.6, 128.6, 129.3, 131.3, 131.5, 132.0, 132.7, 141.8, 142.7.

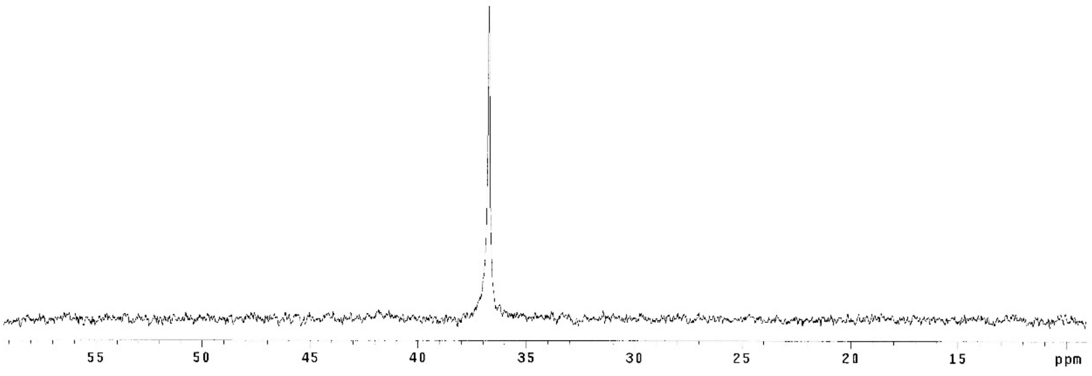

(2S)-2-Phenyl-(5R)-5-phenyl-4-[(1'S)-1-phenylethyl]-2,3,4,5-tetrahydro-1,4,2-benzoxazaphosphepine 2-oxide (**17c**). ^{31}P-NMR ($CDCl_3$, 80.95 MHz): δ 36.7.

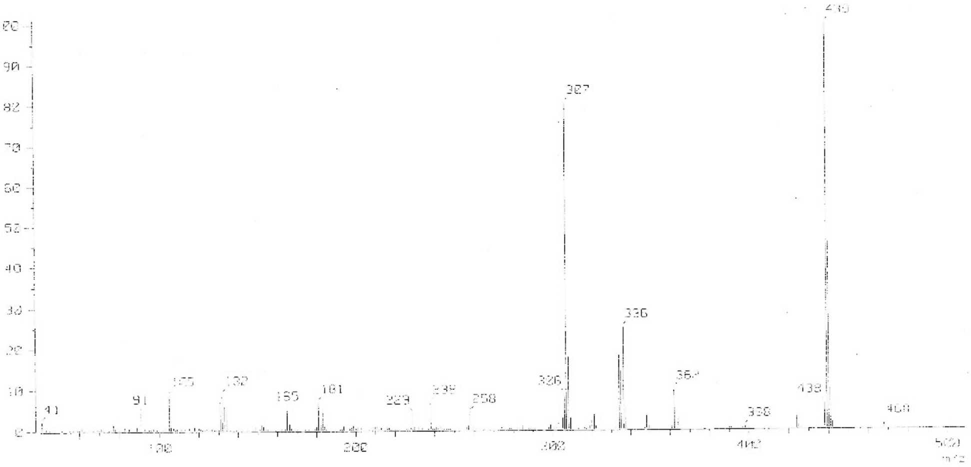

(2S)-2-Phenyl-(5R)-5-phenyl-4-[(1'S)-1-phenylethyl]-2,3,4,5-tetrahydro-1,4,2-benzoxazaphosphepine 2-oxide (**17c**). HRMS (CI^+): found for $[M]^+$, *m*/*z* 439.1772.

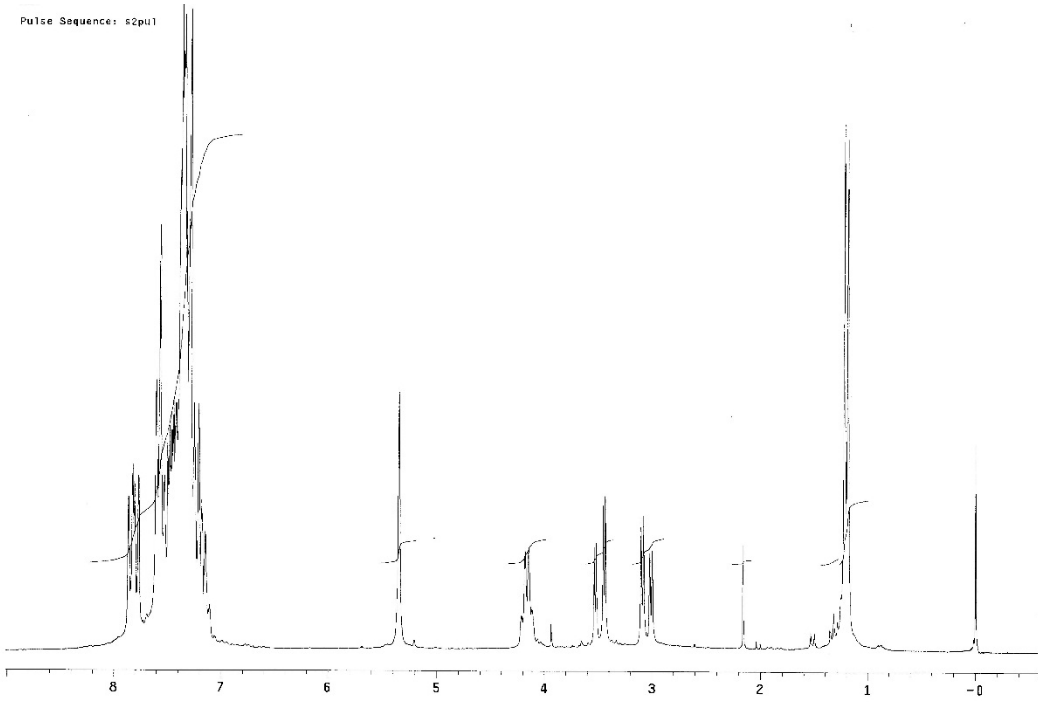

(2R)-2-Phenyl-(5R)-5-phenyl-4-[(1'S)-1-phenylethyl]-2,3,4,5-tetrahydro-1,4,2-benzoxazaphosphepine 2-oxide (**18c**). ^{1}H-NMR ($CDCl_3$, 400 MHz): δ 1.20 (d, *J* = 6.6 Hz, 3H), 3.04 (ABX system, *J* = 16.1, 4.7 Hz, 1H), 3.48 (ABX system, *J* = 16.1, 3.7 Hz, 1H), 4.16 (q, *J* = 7.0 Hz, 1H), 5.35 (s, 1H), 7.14–7.86 (m, 19H).

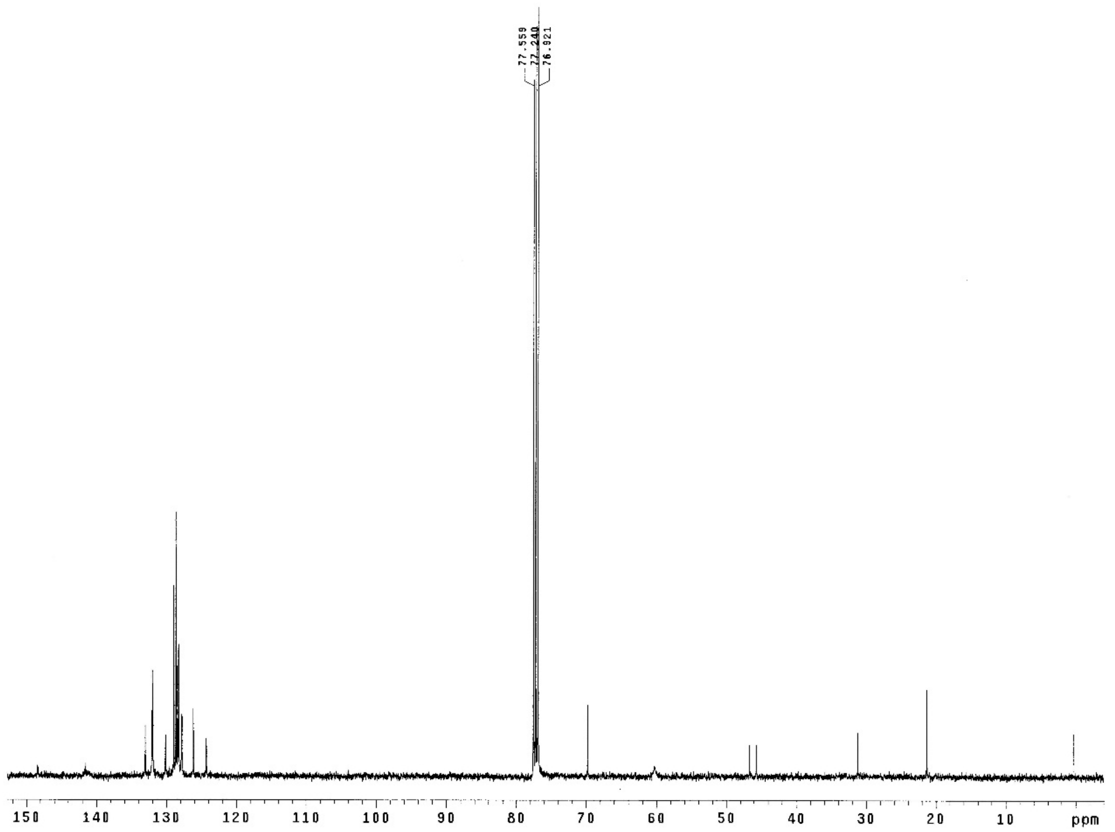

(2R)-2-Phenyl-(5R)-5-phenyl-4-[(1'S)-1-phenylethyl]-2,3,4,5-tetrahydro-1,4,2-benzoxazaphosphepine 2-oxide (**18c**). ^{13}C-NMR ($CDCl_3$, 50 MHz): δ 21.3, 46.1 (d, $J_{C/P}$ = 98.7 Hz), 60.3, 69.7, 124.3, 126.1, 127.7, 127.8, 128.2, 128.5, 128.6, 128.9, 130.2, 131.9, 132.0, 132.1, 133.0, 142.3, 148.0.

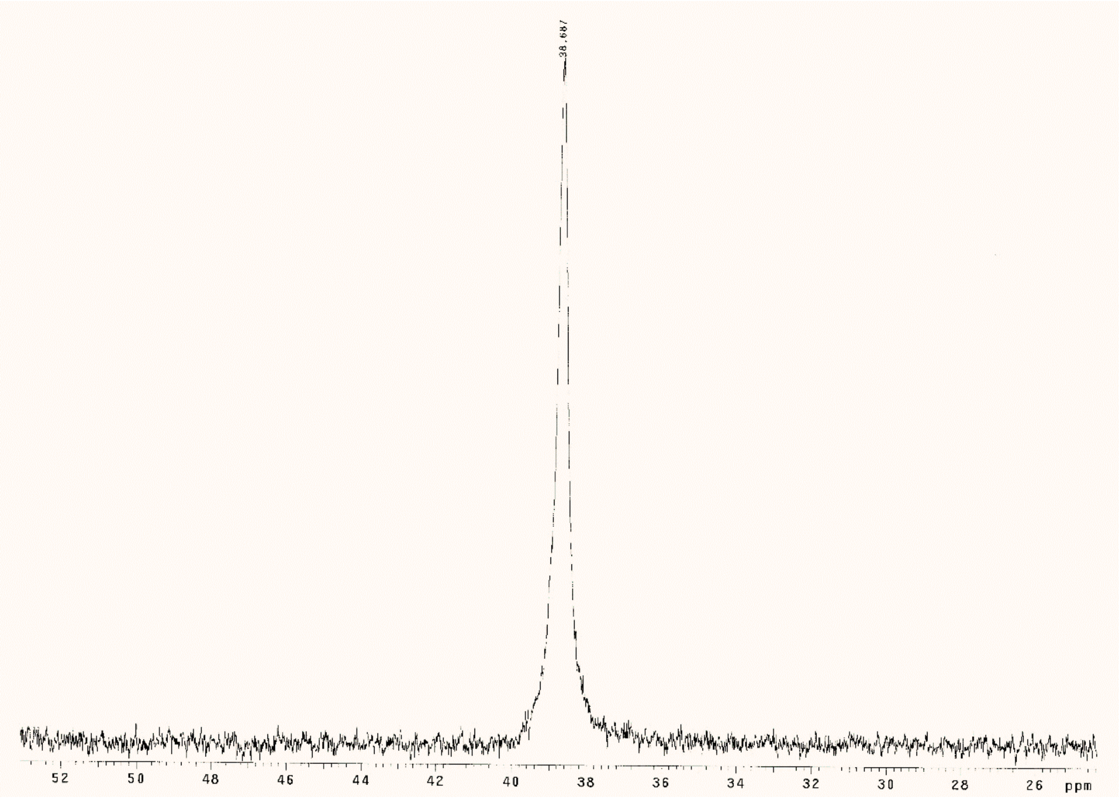

(2R)-2-Phenyl-(5R)-5-phenyl-4-[(1'S)-1-phenylethyl]-2,3,4,5-tetrahydro-1,4,2-benzoxazaphosphepine 2-oxide (**18c**). ^{31}P-NMR ($CDCl_3$, 161.8 MHz): δ 37.84.

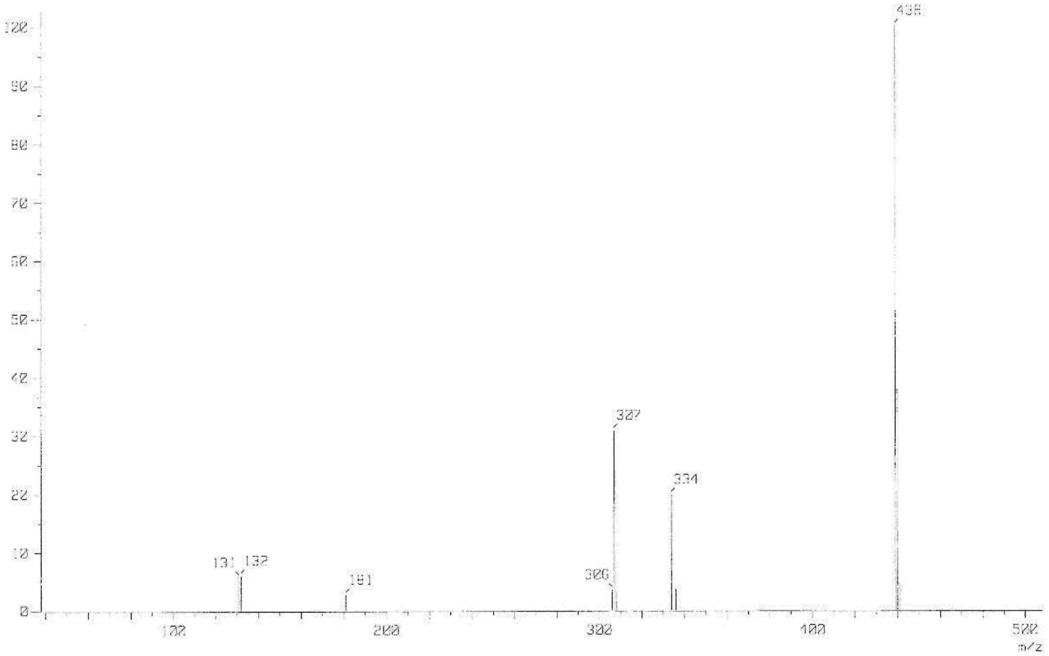

(2R)-2-Phenyl-(5R)-5-phenyl-4-[(1'S)-1-phenylethyl]-2,3,4,5-tetrahydro-1,4,2-benzoxazaphosphepine 2-oxide (**18c**). HRMS (CI$^+$): found for [M + H]$^+$, *m/z* 439.1639.

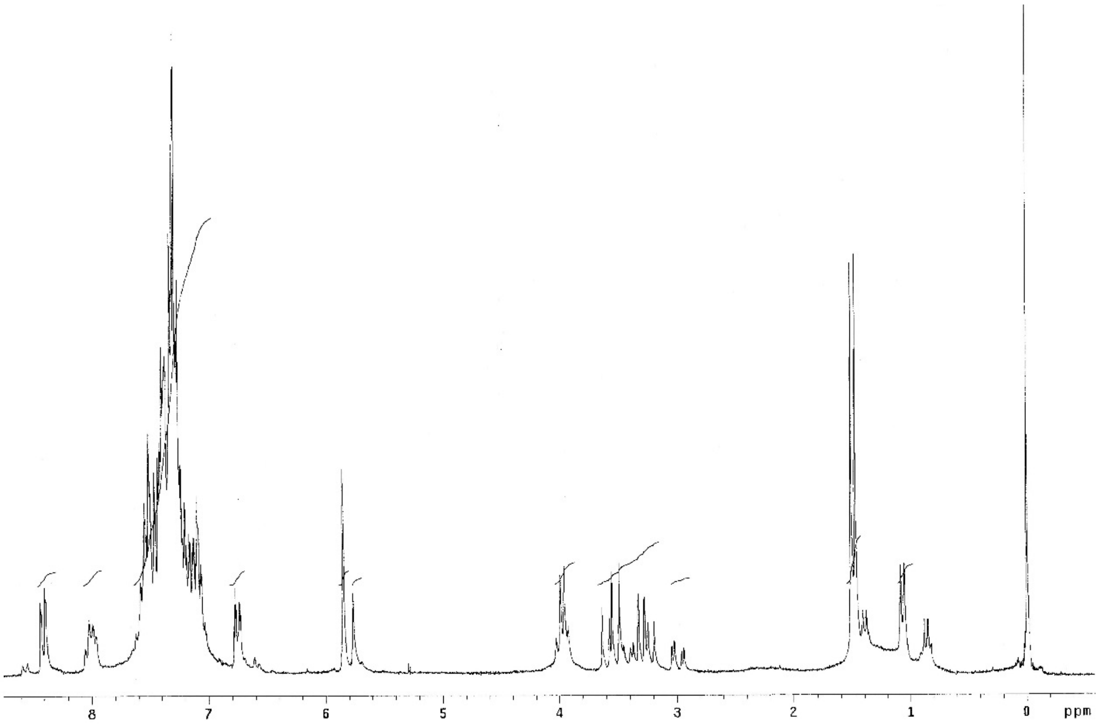

(2R,S)-2-Phenyl-(5S)-5-(2-chlorophenyl)-4-[(1'S)-1-phenylethyl]-2,3,4,5-tetrahydro-1,4,2-benzoxazaphosphepine 2-oxide (**17d**) and (**18d**). The asterisk denotes the minor diastereoisomer.^{1}H-NMR ($CDCl_3$, 200 MHz): δ 1.06* (d, *J* = 7.0 Hz, 3H), 1.49 (d, *J* = 7.0 Hz, 3H), 2.99* (ABX system, *J* = 16.2, 4.2 Hz, 1H), 3.25 (ABX system, *J* = 16.3, 5.8 Hz, 1H), 3.42* (ABX system, *J* = 16.4, 5.4 Hz, 1H), 3.56 (ABX system, *J* = 16.5, 4.4 Hz, 1H), 3.96* (q, *J* = 6.9 Hz, 1H), 3.97 (q, *J* = 6.9 Hz, 1H), 5.76* (s, 1H), 5.85 (s, 1H), 6.72–8.45 (m, 36H).

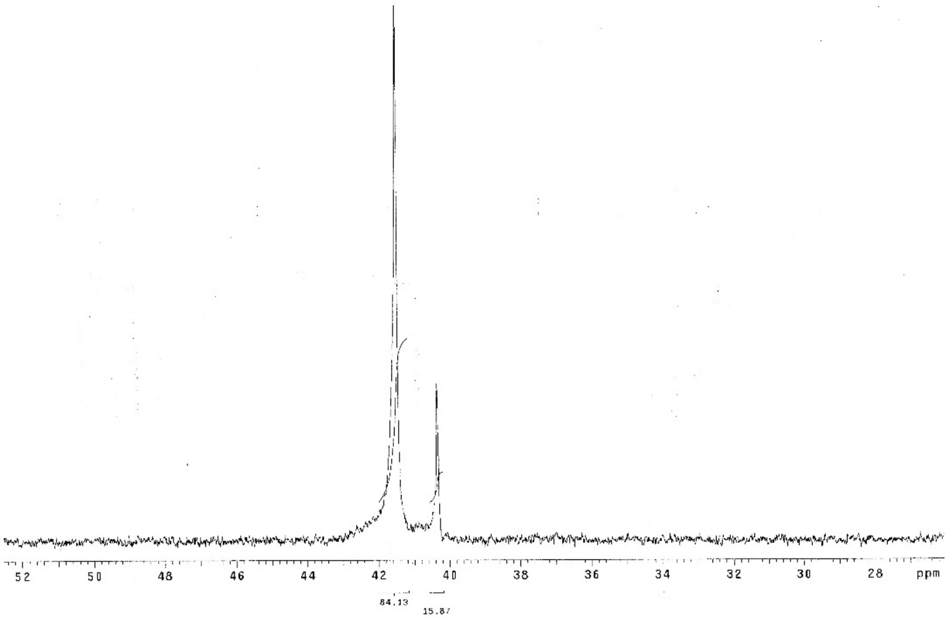

(2R,S)-2-Phenyl-(5S)-5-(2-chlorophenyl)-4-[(1'S)-1-phenylethyl]-2,3,4,5-tetrahydro-1,4,2-benzoxazaphosphepine 2-oxide (**17d**) and (**18d**). ^{31}P-NMR ($CDCl_3$, 81 MHz): δ 40.35*, 41.51.

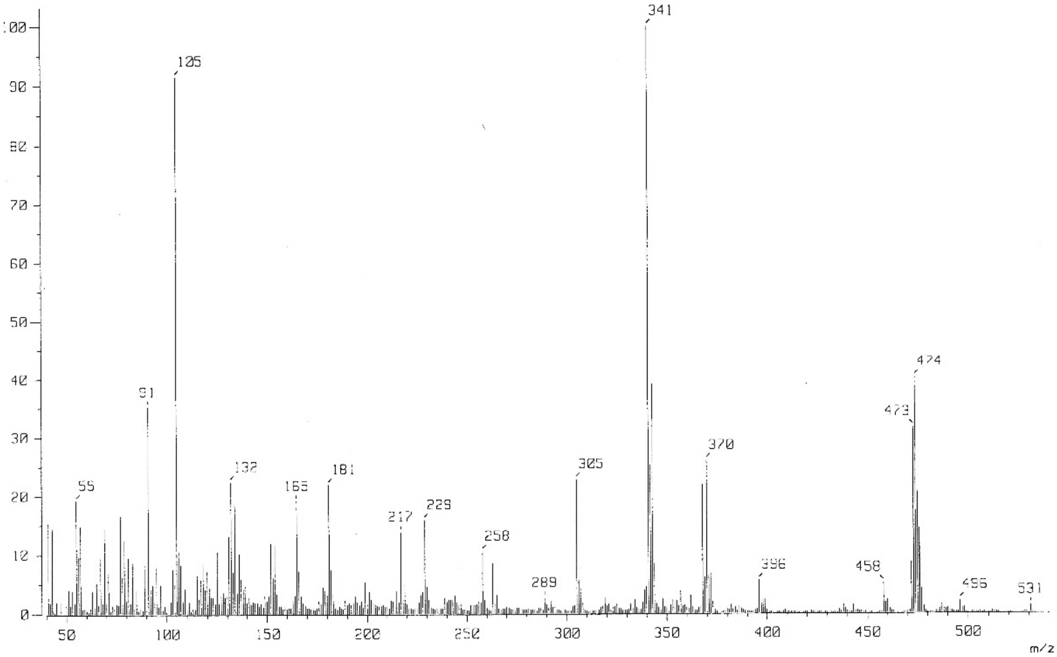

(2R,S)-2-Phenyl-(5S)-5-(2-chlorophenyl)-4-[(1'S)-1-phenylethyl]-2,3,4,5-tetrahydro-1,4,2-benzoxazaphosphepine 2-oxide (**17d**) and (**18d**). HRMS (FAB$^+$): found for $[M + H]^+$, *m*/*z* 474.1389.
